# Uterine infusion of bacteria alters the transcriptome of bovine oocytes

**DOI:** 10.1096/fba.2020-00029

**Published:** 2020-07-16

**Authors:** Rachel L. Piersanti, Jeremy Block, Zhengxin Ma, KwangCheol C. Jeong, José E. P. Santos, Fahong Yu, I. Martin Sheldon, John J. Bromfield

**Affiliations:** ^1^ Department of Animal Sciences University of Florida Gainesville FL USA; ^2^ Interdisciplinary Center for Biotechnology Research University of Florida Gainesville FL USA; ^3^ Institute of Life Science Swansea University Medical School Swansea UK

**Keywords:** endometritis, gene expression, infection, inflammation

## Abstract

Postpartum uterine infection reduces fertility in dairy cattle; however, the mechanisms of uterine infection‐mediated infertility are unknown. Paradoxically, infection‐induced infertility persists after the resolution of disease. Oocytes are a finite resource, which are present at various stages of development during uterine infection. It is likely that oocyte development is influenced by uterine infection‐induced changes to the follicular microenvironment. To better understand the impact of infection on oocyte quality we employed global transcriptomics of oocytes collected from heifers after receiving intrauterine infusion of pathogenic *Escherichia coli* and *Trueperella pyogenes*. We hypothesized that the oocyte transcriptome would be altered in response to intrauterine infection. A total of 452 differentially expressed genes were identified in oocytes collected from heifers 4 days after bacteria infusion compared to vehicle infusion, while 539 differentially expressed genes were identified in oocytes collected from heifers 60 days after bacteria infusion. Only 42 genes were differentially expressed in bacteria‐infused heifers at both Day 4 and Day 60. Interferon, HMGB1, ILK, IL‐6, and TGF‐beta signaling pathways were downregulated in oocytes collected at Day 4 from bacteria‐infused heifers, while interferon, ILK, and IL‐6 signaling were upregulated in oocytes collected at Day 60 from bacteria‐infused heifers. These data suggest that bacterial infusion alters the oocyte transcriptome differently at Day 4 and Day 60, suggesting different follicle stages are susceptible to damage. Characterizing the long‐term impacts of uterine infection on the oocyte transcriptome aids in our understanding of how infection causes infertility in dairy cattle.

## 1 INTRODUCTION

Infection of the postpartum uterus with bacteria often causes endometritis and reduces fertility in dairy cattle.^1^ Clinical endometritis occurs 21 days or more after calving and is characterized by the presence of purulent or mucopurulent uterine discharge, usually detected in the vagina.^1^ The incidence of clinical endometritis varies amongst herds but generally occurs in 20% of postpartum cows.^2^, ^3^ Endometritis increases the time from parturition to first insemination and delays conception, even after the resolution of clinical disease,^4^ and results in increased rates of culling for reproductive insufficiency.^2^ The mechanisms by which endometritis results in infertility are unclear, but likely include perturbations to the physiology of the uterus, hypothalamic‐pituitary axis and the ovary.^5^

Clinical endometritis is associated with several pathogenic bacteria including Gram‐negative *Escherichia coli*, *Prevotella* sp, *Fusobacterium necrophorum*, and *Fusobacterium nucleatum,* and Gram‐positive *Trueperella pyogenes*.^6^, ^7^ Two pathogens commonly associated with endometritis are *E coli* and *T pyogenes*, which in combination can induce clinical endometritis when administered intrauterine to virgin Holstein heifers.^8^ The cell wall component of Gram‐negative bacteria, lipopolysaccharide (LPS), is concentrated in the follicular fluid of cows with uterine infection and is positively correlated with the degree of endometrial inflammation,^9^ while LPS is still present in the follicular fluid of cows after the resolution of uterine infection.^10^ Cows with uterine infection have slower growing ovarian follicles and lower peripheral plasma estradiol concentrations than healthy cows.^7^, ^11^ In vitro, granulosa cells respond to LPS via the Toll‐like receptor 4 pathway by increasing the secretion of inflammatory mediators and reducing the synthesis of estradiol.^9^, ^12^

Uterine infection‐induced changes to the follicle microenvironment are evident 40 days after the clearance of infection, likely due to exposure of granulosa cells to bacterial components such as LPS.^10^ Therefore, it is important to consider the effects of an altered follicle microenvironment on the growth and development of the oocyte. Oocytes matured in the presence of LPS have increased meiotic failure and a reduced capacity to develop to a blastocyst after in vitro fertilization and embryo culture.^12^, ^13^ Similarly, mammary gland infection in cows also reduces the capacity of oocytes to develop to a blastocyst following in vitro fertilization and embryo culture.^14^ This raises the question, does uterine infection alter oocyte competence that leads to infertility?

The development of a follicle from the primordial stage to ovulation is estimated to take in excess of 100 days in the cow.^15^ However, it is unclear if oocytes within follicles of various stages of development are susceptible to the negative effects of uterine infection, or if these effects are persistent. To understand the effect of uterine disease on the oocyte, we induced uterine infection in virgin heifers by intrauterine infusion of pathogenic *E coli* and *T pyogenes*. Oocytes were collected by transvaginal‐ultrasound‐guided follicle aspiration at Day 4 and Day 60 after infusion and subjected to global transcriptome analysis. We hypothesized that the oocyte transcriptome would be altered in response to intrauterine bacterial infusion. These data will enable us to better understand the consequence of uterine infection on the oocyte, and potential mechanisms by which infection reduces fertility in cattle.

## 2 MATERIALS AND METHODS

### 2.1 Induction and clinical evaluation of endometritis in virgin holstein heifers

**FIGURE 1:**
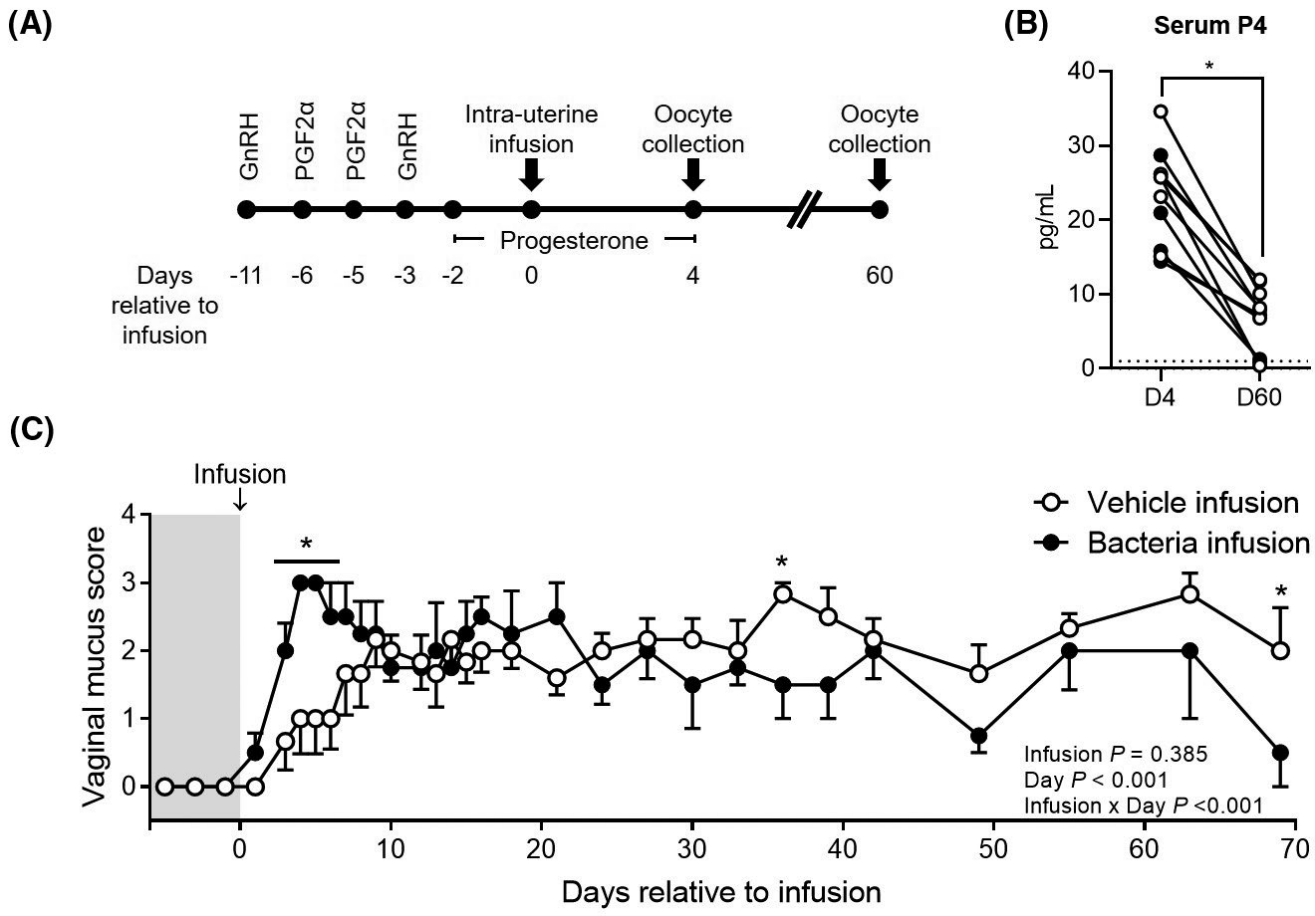
Induction and verification of experimental uterine infection. A, Estrous cycles of 10 virgin heifers were synchronized prior to intrauterine infusion of vehicle or pathogenic bacteria on Day 0. Exogenous supplementation of progesterone and endometrial scarification were performed prior to intrauterine infusion. Oocyte collection by transvaginal follicle aspiration occurred on Day 4 and Day 60 relative to intrauterine infusion. B, vaginal mucus score was recorded on the indicated days, and (C) serum progesterone was measured on Day 4 and Day 60 relative to intrauterine infusion: vehicle‐infused heifers (○, n = 6), bacteria‐infused heifers (●, n = 4). Vaginal mucus score is presented as mean ± SEM, with data analyzed by ANOVA; values differ between infusion group, within day, * *P* ≤ .05

All procedures performed on heifers were conducted from June to October 2017 at the University of Florida Dairy Research Unit. Heifers and procedures used to induce clinical endometritis in virgin Holstein heifers were previously described by Piersanti.^8^ Ten virgin Holstein heifers aged between 11 and 13 months were used for the experiment. Estrous cycles were synchronized by administering 100 μg im of GnRH (gonadorelin diacetate tetrahydrate; OvaCyst, Bayer HealthCare LLC) followed by 25 mg im of PGF2α (dinoprost tromethamine; ProstaMate, Bayer HealthCare LLC) administered 5 and 6 d later. Eight days following initial GnRH, heifers received a final dose of 100 μg of GnRH im starting on the day following the end of the synchronization protocol, heifers received 200 mg im of progesterone in corn oil (50 mg/mL; Sigma‐Aldrich) daily for 7 days. Heifers were randomly assigned to receive intrauterine infusion of vehicle control (n = 6) or live pathogenic bacteria (n = 4). Intrauterine infusion (Day 0) was performed 3 days after the end of the synchronization protocol (Figure 1A). Briefly, endometrial scarification preceded the intrauterine infusion of either vehicle medium (30 mL of sterile Luria‐Bertani (LB) broth) or pathogenic bacteria (10 mL of 4.64 × 10^7^ CFU/mL of *Escherichia coli* MS499, 10 mL of 3.38 × 10^7^ CFU/mL of *Trueperella pyogenes* MS249 followed by 10 mL of sterile LB broth).^16^, ^17^

Endometritis was repeatedly evaluated and scored using visual assessment of vaginal mucus collected using a clean Metricheck tool (Simcro) between Days 1 and 69. Vaginal mucus was assessed daily between Day 1 and 18, then every third day until Day 42 and then weekly until Day 69. Vaginal mucus was scored as grade 0, no mucus or clear or translucent mucus; grade 1, mucus containing flecks of white or off‐white pus; grade 2, mucus containing ≤ 50% white or off‐white mucopurulent material; and grade 3, mucus containing > 50% purulent material.^18^

Serum progesterone was measured on Day 4 and Day 60 using a commercial ELISA (DRG International, Inc) according to the manufacturer's instructions. The progesterone ELISA is designed for human blood and was validated for bovine plasma using spike‐in/recovery performance based on actual and expected recovery of progesterone supplied as standard with the kit. Intra‐assay CV was 6.5%, while recovery of spike‐in progesterone was 89.0% to 101.8% of expected progesterone.

### 2.2 Follicle aspiration and ovum pickup

Ovum pickup was performed using transvaginal ultrasound‐guided follicle aspiration performed on Days 4 and 60 (Figure 1A). Briefly, heifers received a caudal epidural injection of 60 mg of lidocaine hydrochloride 2% (Aspen Veterinary Resources), and the perineum and vulva were cleaned and disinfected with povidone followed by 70% ethanol. A vaginal lavage using 100 mL of 0.2% chlorhexidine in saline followed by two 100 mL lavages using sterile 0.9% saline was employed to wash the vagina. An ovum pickup handle including a 5 mHz convex ultrasound probe (Choice Medical Systems, Inc) was covered in a sanitary cover sleeve (TNB) and introduced into the vagina with sterile lubricant. Using rectal palpation, the ovary was placed toward the ultrasound probe and visualized using an Aloka ultrasound (Aloka SSD‐500, Hitachi Healthcare Americas). A 20 G aspiration needle attached to a vacuum pump was introduced into the ovum pickup handle. If a dominant follicle (>8 mm diameter) was present it was ablated, and the follicle content was discarded. Using ultrasound guidance all follicles less than 8 mm in diameter were aspirated into OPU collection medium (Medium 199 with Earle's Salts, 0.5% BSA, 20mM HEPES, 2mM sodium pyruvate, 10 IU/mL of heparin, 100 U/mL of penicillin and 100 µg/mL of streptomycin; all Fisher Scientific). The follicular aspirate was filtered using an oocyte filter (WTA), washed with 30 to 50 mL of OPU collection medium and placed into a gridded petri dish (Fisher Scientific) for oocyte searching under a stereo microscope. Aspirated cumulus oocyte complexes (COCs) were washed twice in fresh Dulbecco phosphate‐buffered saline (DBPS) containing 0.1% polyvinylpyrrolidone. Removal of cumulus cells was achieved by placement of COCs in 1000 U/mL of hyaluronidase in HEPES‐Tyrode albumin lactate pyruvate (TALP) for 4 minutes and oocytes were manually denuded using a Stripper pipette (Cooper Surgical). Zona pellucida of denuded oocytes was subsequently removed using 0.1% protease from *Streptococcus griseus* (Sigma‐Aldrich), and washed three times in fresh DPBS‐PVP. Zona‐free oocytes were pooled from a single heifer, snap frozen and stored at −80°C until further processing. One vehicle‐infused heifer at Day 4, and one bacteria‐infused heifer at both Day 4 and Day 60 did not undergo ovum pickup due to limitations of body size and trauma to the rectum.

### 2.3 RNA extraction and sequencing of oocyte transcriptome

Total RNA was extracted from oocytes collected from each individual heifer infused with vehicle (n = 5 heifers, 28 oocytes) or bacteria (n = 3 heifers, 15 oocytes) at Day 4, and vehicle (n = 6 heifers, 11 oocytes) and bacteria (n = 3 heifers, 9 oocytes) at Day 60. Oocytes from each heifer were maintained as single, unique replicates throughout processing and analysis. Oocytes were thawed and suspended in 350 μL of RLT buffer for the extraction of total RNA using the RNeasy Micro kit (QIAGEN Inc) according to the manufacturer's instructions. Total RNA concentration was determined using a Qubit 2.0 Fluorometer (Thermo Fisher Scientific Inc) and RNA quality was assessed using an Agilent 2100 Bioanalyzer (Agilent Technologies). To produce RNAseq libraries for sequencing, 200 pg of total RNA was used for library construction using SMARTer Universal Low input RNA kit (Takara Bio USA, Inc), combined with Illumina Nextera DNA Library Preparation Kit (Illumina Inc), according to the manufacturer's instructions. Briefly, first strand cDNA was primed by a modified N6 primer (the SMART N6 CDS primer), then base‐paired with additional nucleotides, creating an extended template. The reverse transcriptase then switches templates and continues transcribing to the end of the oligonucleotide, resulting in single‐stranded cDNA containing sequences that are complementary to the SMARTer oligonucleotide. The SMARTer anchor sequence and the N6 sequence then served as universal priming sites for DNA amplification by PCR for 10 cycles. A total of 125 pg of cDNA was then fragmented by a tagementation reaction and adapter sequences were added to the template cDNA by PCR amplification. Libraries were quantitated by Bioanalyzer and qPCR (Kapa Biosystems, Inc). Individual libraries were pooled at equal molar concentration and a total of 17 samples were divided and run in two lanes on an Illumina HiSeq3000 (Illumina). RNA library construction and sequencing were performed at the University of Florida Interdisciplinary Center for Biotechnology Research. Data collected here have been deposited in the NCBI’s Gene Expression Omnibus and are accessible through GEO Series accession number GSE141307.

### 2.4 Read mapping and gene expression analysis

Reads acquired from the sequencing platform were cleaned with the Cutadapt program^19^ to trim off sequencing adaptors, low quality bases, and potential errors introduced during sequencing or library preparation. Reads with a quality Phred‐like score < 20 and read length < 40 bases were excluded from RNAseq analysis.

The NCBI Genome database transcripts of *Bos taurus* (80 896 sequences) were used as a reference for RNAseq analysis. The cleaned reads of each sample were mapped individually to the reference sequences using the bowtie2 mapper (v. 2.2.3) with a “3 mismatches a read” allowance.^20^ The mapping results were processed with the samtools and scripts developed in‐house at the University of Florida Interdisciplinary Center for Biotechnology Research to remove potential PCR duplicates and choose uniquely mapped reads for gene expression analysis. Differentially expressed genes (DEGs) in bacteria‐infused heifers (compared to vehicle‐infused heifers) were identified at each time point (Day 4 or Day 60) by counting the number of mapped reads for each transcript.^21^ Differentially expressed genes were defined using the *P*‐value and log_2_ fold change (FC) ≥ 2 or ≤ −2. Adjusted *P*‐values ≤ 0.1 were used initially in addition to non‐adjusted *P*‐values ≤ .05.

### 2.5 Ingenuity pathway analysis of differentially expressed genes

Pathway analysis was performed using Ingenuity Pathway Analysis (Qiagen). Differentially expressed genes with the criteria of log_2_ FC ≥ 2 or ≤ −2, and a non‐adjusted *P*‐value ≤ .05 were used for analysis. These criteria were applied in order to maintain uniformity throughout the investigation due to the disparity in the number of DEGs following false discovery rate analysis using the adjusted *P*‐value. Represented canonical pathways with a ‐log *P*‐value > 1.3 were determined with corresponding *z*‐scores to describe predicted activation status. Represented gene networks were determined by assessing the number of DEGs in each gene network. Upstream regulators of specific gene networks and upstream regulators of DEGs were predicted using Ingenuity Pathway Analysis algorithms. Predicted upstream regulators of DEGs were assigned an activation z‐score to predict either upregulation or down regulation of various downstream DEGs. A z‐score ≥ 2 or ≤ −2 was assumed to be a significant prediction of activation or inhibition, respectively.

### 2.6 Statistical analysis

Vaginal mucus score was analyzed using SAS v. 9.4 (SAS Institute). Vaginal mucus scores were analyzed using the GLIMMIX procedure of SAS. The model included the fixed effects of treatment and day, and heifer nested within treatment as a random effect, in a first order autoregressive covariance structure. Vaginal mucus scores are reported as means ± SEM, and *P* ≤ .05 was considered statistically significant. For all RNAseq data the heifer were considered as the statistical unit, with all oocytes from a single collection procedure (one heifer, at one time point) pooled for library preparation. Heatmaps were generated based on complete linkage clustering and Pearson correlation for distance using the online tool Heatmapper.^22^ Principal component analysis plots were performed using online ClustVis tools.^23^

## 3 RESULTS

### 3.1 Vaginal mucus score

Heifers in both groups had a mean vaginal mucus score of 0 prior to intrauterine infusion (Day 0). Overall vaginal mucus score did not differ between infusion groups during the 69‐day experimental period (*P* = .385). However, bacteria‐infused heifers had an increased (*P* ≤ .05) vaginal mucus score on Days 3, 4, 5, and 6 compared to vehicle‐infused heifers (Figure 1B). Serum progesterone was measured at Day 4 and Day 60. Serum progesterone (Figure 1C) was assessed first by the fixed effect of treatment and was not effected overall by bacterial infusion (16.6 ± 1.5 ng/mL in vehicle‐infused heifers versus 12.1 ± 1.9 ng/mL in bacteria‐infused heifers); however, overall there was a significant (*P* < .001) reduction in serum progesterone on Day 60 compared to Day 4 (6.25 ± 1.72 ng/mL and 22.51 ± 1.76 ng/mL, respectively). At Day 4 serum progesterone was measured between 14.41 ng/mL and 34.58 ng/mL, while at Day 60 serum progesterone ranged from 0.38 ng/mL to 11.94 ng/mL, with one heifer in each treatment group having progesterone below 1 ng/mL.

### 3.2 Differentially expressed genes in oocytes during active disease and following disease resolution

At Day 4, an average of 5.6 ± 2.5 oocytes were collected from vehicle‐infused heifers (28 oocytes total), and an average of 5.0 ± 1.7 oocytes from bacteria‐infused heifers (15 oocytes total). At Day 60 an average of 1.8 ± 1.0 oocytes were collected from vehicle‐infused heifers (11 oocytes total), and an average of 3.0 ± 1.0 oocytes were collected from bacteria‐infused heifers (9 oocytes total). Following RNAseq and read processing, a total of 1 224 781 053 high quality reads were used for analysis (Table S1). An average of 35.8 ± 0.7% high quality reads were aligned to the reference genome, resulting in an average of 17,270 ± 450 transcripts mapped to the reference. Of the 10 genes of greatest transcript abundance detected in all oocytes (Table 1), oocyte‐specific genes including *ZP3*, *ZP4* and *BMP15* were highly abundant. Principal component analysis of all RNA transcripts obtained from RNAseq describe cross‐over between the various comparisons (Figure S1). Indeed, there was no distinct separation of treatment profiles between bacteria‐infused heifers vehicle‐infused heifers at either Day 4 or Day 60, nor was there a separation of profiles when comparing day of collection within treatment groups.

**TABLE 1:** Highest expressed genes in aspirated oocytes from all treatments and time points

Gene	Gene name	Mean reads^a^	Accession number
*ACCSL*	1‐aminocyclopropane‐1‐carboxylate synthase (inactive)‐like (predicted)	431355	XM_002693559.4
*ZP3*	Zona pellucida glycoprotein 3	359291	NM_173974.3
*ZP4*	Zona pellucida glycoprotein 4	342743	NM_173975.2
*BTG4*	BTG family member 4 (predicted)	256482	XM_010812381.2
*GSTM3*	Glutathione S‐transferase mu 3	230038	NM_001046560.1
*HMGN2*	High mobility group nucleosomal binding domain 2	163548	NM_001098945.1
*BMP15*	Bone morphogenetic protein 15 (predicted)	149463	XM_010821881.2
*GSTA3*	Glutathione S‐transferase alpha 3	125780	NM_001078149.1
*ACTB*	Actin beta	122733	NM_173979.3
*KPNA7*	Karyopherin subunit alpha 7	114589	NM_001163947.1

^a^ Base mean values determined by RNAseq read number.

**FIGURE 2:**
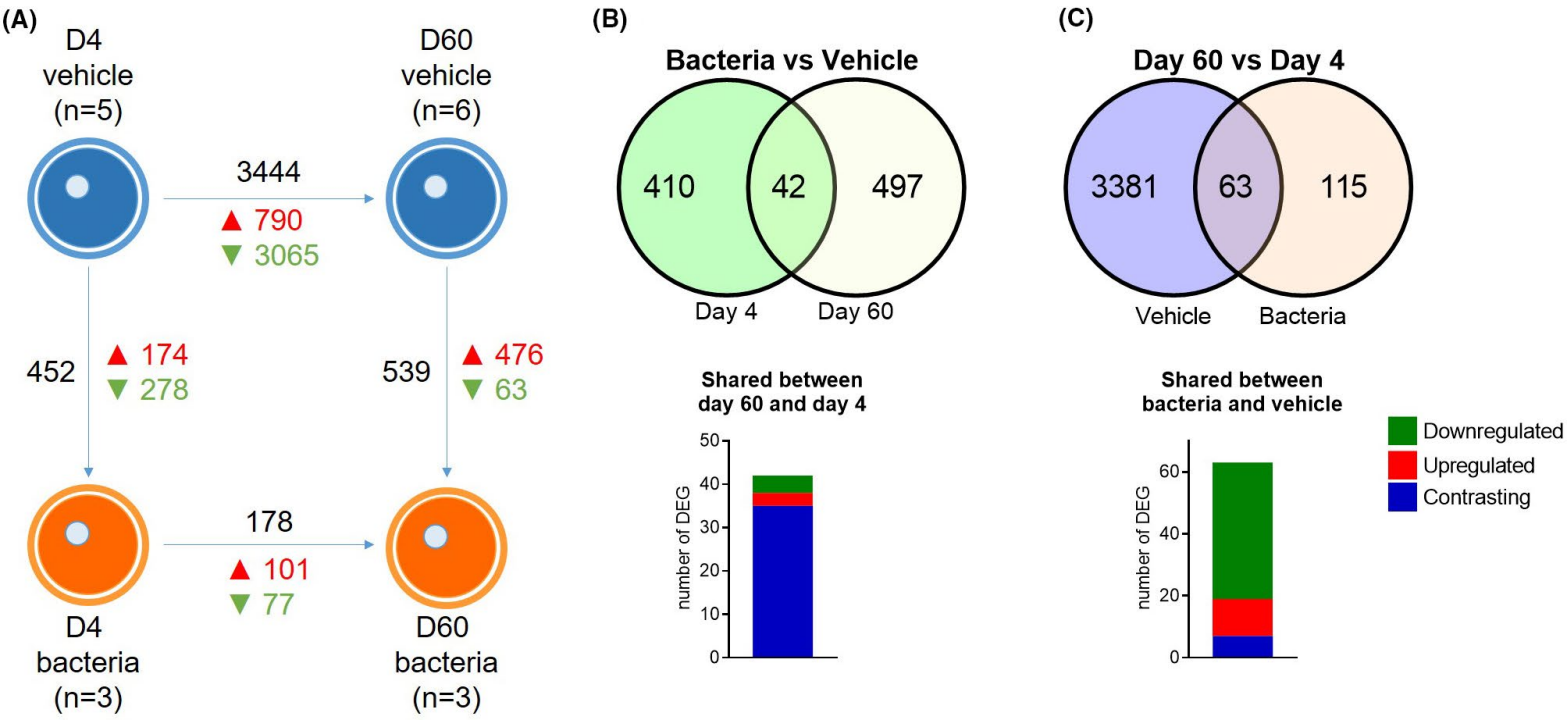
Differentially expressed genes in oocytes collected after intrauterine infusion. Following RNAseq analysis, DEGs (A) were identified in oocytes collected at Day 4 or Day 60 from heifers infused with either vehicle medium (blue, n = 5 heifers at Day 4, and n = 6 heifers at Day 60) or pathogenic bacteria (orange, n = 3 heifers at both days). Arrows indicate comparisons between samples. Total number of DEGs (black), upregulated genes (red) and downregulated genes (green) are shown based on the criteria of a log_2_ FC ≥ 2 or ≤ −2, and *P* ≤ .05. Venn diagrams show the number of DEGs in oocytes collected from bacteria‐infused heifers compared to vehicle‐infused heifers at Day 4 and Day 60 (B), and DEGs in oocytes collected at Day 4 and Day 60 within infusion groups (C). The number of DEGs that was upregulated (red), downregulated (green), or contrasting (blue) in expression between comparisons are shown

**FIGURE 3:**
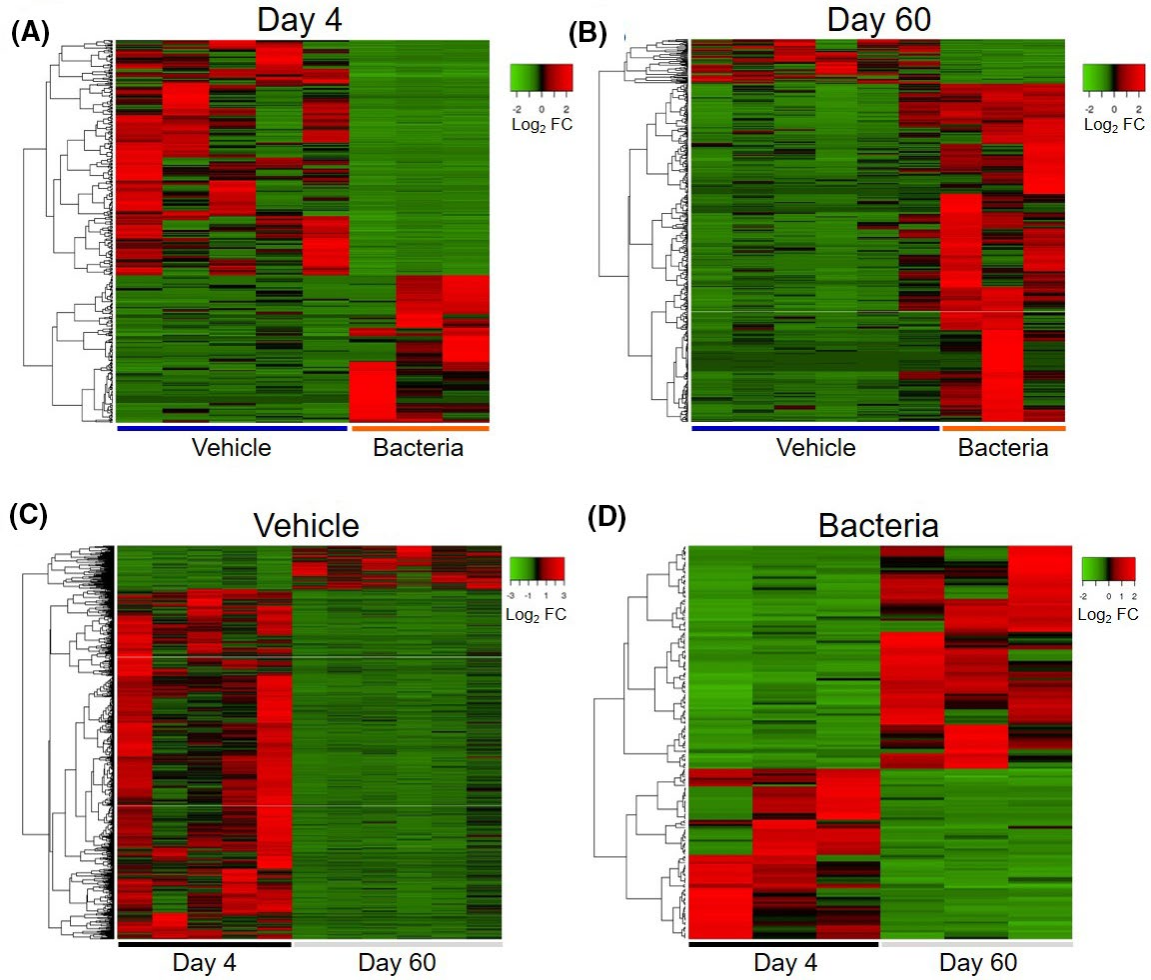
Heatmaps of differentially expressed genes in oocytes following intrauterine infusion. Heatmaps were generated for DEGs identified in oocytes collected at Day 4 (A) or Day 60 (B) from heifers infused with vehicle medium (blue) or pathogenic bacteria (orange). Additional heatmaps were generated to compare DEGs of oocytes collected from vehicle‐infused (C) or bacteria‐infused (D) heifers at Day 4 (black) and Day 60 (grey). Heatmaps indicate hierarchical clustering based on complete linkage and Pearson correlation of genes with expression log_2_ fold change (FC) ≥ 2 or ≤ −2 and, *P* ≤ .05

Using an adjusted *P*‐value ≤ 0.1, 10 DEGs were observed in oocytes of bacteria‐infused heifers compared to vehicle‐infused heifers on Day 4, and 47 DEGs at Day 60 (Tables S2 and S3). Using less stringent criteria of log_2_ FC ≥ 2 or ≤ −2, and a non‐adjusted *P* ≤ .05, 452 DEGs were observed in oocytes of bacteria‐infused heifers compared to vehicle‐infused heifers on Day 4, and 539 DEGs at Day 60 (Figure 2A and Table 2). Of the 452 DEGs at Day 4, 174 genes were upregulated, and 278 genes were downregulated (Table S4). Of the 539 DEGs at Day 60, 476 were upregulated, and 63 were downregulated (Table S5). Interestingly, only 42 DEGs were identified in oocytes at both Day 4 and Day 60. Of these 42 DEG, only 4 genes were upregulated on both days and 3 genes were downregulated on both days, with the remaining 35 genes downregulated at Day 4 and then upregulated at Day 60 (Figure 2B). Heatmaps with hierarchical clustering of DEGs show the expression profiles of oocytes from control and bacteria‐infused heifers at Day 4 and Day 60 (Figure 3A,B).

**TABLE 2:** Greatest differentially expressed genes in oocyte from bacteria infused heifers compared to vehicle infused heifers at Day 4 and Day 60

Gene	Gene name	Log_2_ FC^a^	*P*‐value
Day 4			
*LOC530102*	Collagen alpha‐6 (VI) chain	11.11	<.001
*SMLR1*	Small leucine‐rich protein 1	10.61	.013
*POLR2K*	RNA polymerase II subunit K	10.17	<.001
*LOC104969009*	Uncharacterized LOC104969009, ncRNA	10.09	.005
*KANSL3*	KAT8 regulatory NSL complex subunit 3	9.32	.022
*CCL2*	Chemokine (C‐C motif) ligand 2	−12.25	.011
*TNFAIP6*	TNF alpha induced protein 6	−11.08	.018
*SNAI2*	Snail family transcriptional repressor 2	−10.90	<.001
*SDC1*	Syndecan 1	−10.25	<.001
*CSRP3*	Cysteine and glycine rich protein 3	−9.79	<.001
Day 60			
*TNFAIP6*	TNF alpha induced protein 6	12.23247	.003
*GPR50*	G protein‐coupled receptor 50	10.99542	.006
*CSRP3*	Cysteine and glycine rich protein 3	10.06804	.008
*LOC104972545*	Uncharacterized LOC104972545, ncRNA	9.214424	.014
*PALMD*	Palmdelphin	9.202837	.014
*ADAM22*	ADAM metallopeptidase domain 22	−8.19946	<.001
*CALHM4*	Calcium homeostasis modulator family member 4	−6.08507	.024
*CPNE4*	Copine IV, partial mRNA	−6.01578	.014
*DNER*	Delta/notch like EGF repeat containing	−5.96741	.006
*LOC101907813*	Uncharacterized LOC101907813, ncRNA	−5.79885	.031

Note

Genes are differentially expressed (log_2_ fold change ≥ 2 or ≤ −2 and a *P* ≤ .05) in oocytes collected from bacteria infused heifers compared to heifers infused with vehicle medium at Day 4 or Day 60 relative to infusion.

^a^ FC, fold change.

When assessing gene expression of oocytes collected from vehicle‐infused heifers, 941 DEGs were identified at Day 60 compared to Day 4 using an adjusted *P*‐value ≤ 0.1, and 3444 DEGs using a less stringent criteria of log_2_ FC ≥ 2 or ≤ −2, and a non‐adjusted *P* ≤ .05 (Figure 2C). Among the 3444 DEGs in vehicle oocytes collected at Day 60, 790 were upregulated and 3065 were downregulated compared to vehicle oocytes collected at Day 4 (Figure 2A). When comparing gene expression between Day 60 and Day 4 for oocytes collected from bacteria‐infused heifers, no DEGs were identified in oocytes from Day 60 using the adjusted *P*‐value ≤ 0.1, while 178 DEGs were identified in Day 60 oocytes of bacteria‐infused heifers using a less stringent criteria of log_2_ FC ≥ 2 and a non‐adjusted *P* ≤ .05 (Figure 2C). Of the 178 DEGs in oocytes of bacteria‐infused heifers, 101 genes were upregulated, and 77 genes were downregulated at Day 60 compared to Day 4 (Figure 2C). Heatmaps for comparisons between Day 4 and Day 60, within treatment groups are shown in Figure 3C,D.

### 3.3 Pathway analysis of differentially expressed genes between treatments during active disease and following disease resolution

**FIGURE 4:**
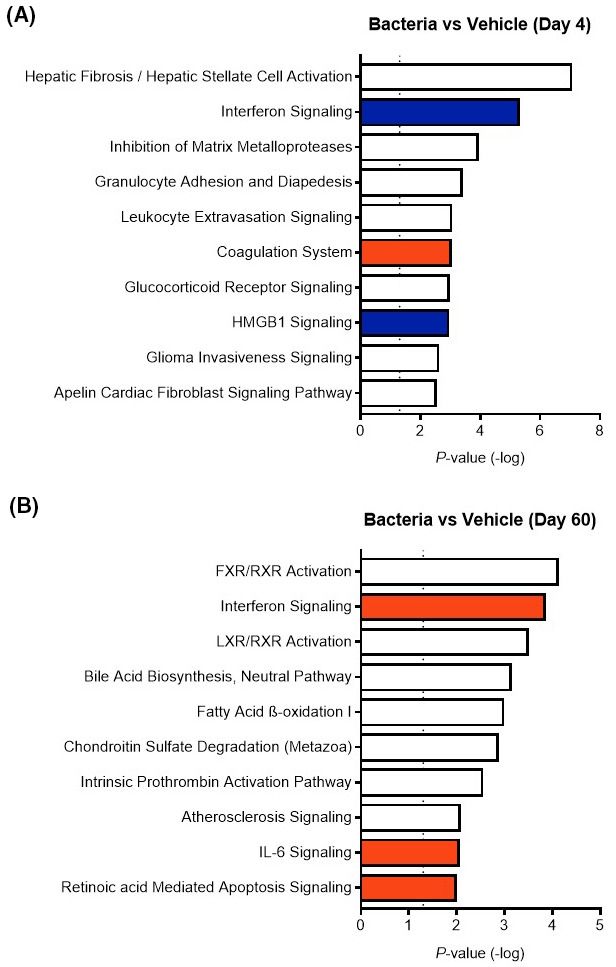
Overrepresented canonical pathways in oocytes following intrauterine infusion. Canonical pathways were identified based on DEGs in oocytes collected from bacteria‐infused heifers compared to vehicle‐infused heifers, at both Day 4 (A) and Day 60 (B). The top 10 overrepresented canonical pathways are shown here. Each canonical pathway was assigned a *z*‐score to predict the pathway activation state as either downregulated (blue), upregulated (orange), or not determined (white). A *z*‐score ≥ 2 or ≤ −2 predicts significant effects based on a ‐log *P* value ≥ 1.3. A complete list of affected canonical pathways can be found in Tables S5 and S6

Analysis of DEGs (log_2_ FC ≥ 2 or ≤ −2, and non‐adjusted *P* ≤ .05) using Ingenuity Pathway Analysis was carried out to estimate effects of DEGs on canonical pathways involved in cellular metabolism and signaling, gene networks and upstream regulators of DEGs. A total of 69 differentially regulated canonical pathways were identified at Day 4 in oocytes collected from bacteria‐infused heifers compared to vehicle‐infused heifers (Table S6). A total of 26 canonical pathways were identified at Day 60 in oocytes collected from bacteria‐infused heifers compared to vehicle‐infused heifers (Table S7). Canonical pathways affected in oocytes obtained from bacteria‐infused heifers at Day 4 include Hepatic Fibrosis/ Hepatic Stellate Cell Activation, Interferon Signaling, Inhibition of Matrix Metalloproteases and, Granulocyte Adhesion and Diapedesis (Figure 4A). Canonical pathways affected in oocytes obtained from bacteria‐infused heifers at Day 60 include FXR/RXR Activation, Interferon Signaling, LXR/RXR Activation, and Bile Acid Biosynthesis, Neutral Pathway (Figure 4B). Canonical pathways identified in oocytes within a treatment group compared between days of collection are shown in Tables S8 and S9.

**FIGURE 5:**
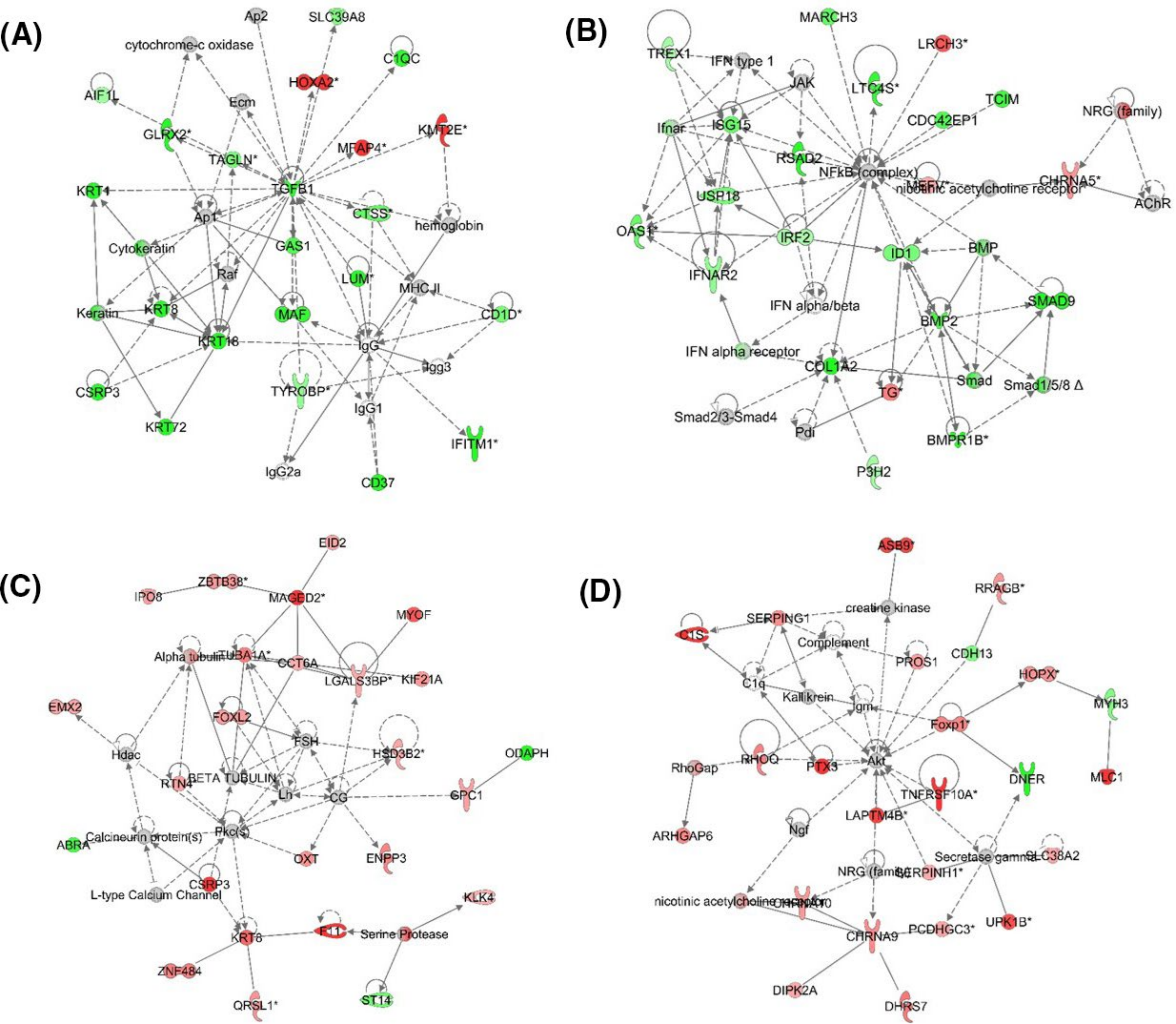
Overrepresented gene networks in oocytes following intrauterine infusion. Differentially expressed genes in oocytes collected from bacteria‐infused heifers compared to vehicle‐infused heifers at Day 4 (A‐B) are associated with cell morphology, cell to cell signaling and interaction, connective tissue development and function (A), and anti‐microbial response, connective tissue development and function, inflammatory response (B). Differentially expressed genes in oocytes collected from bacteria‐infused heifers compared to vehicle‐infused heifers at Day 60 (C‐D) are associated with dental disease, developmental disorder, gastrointestinal disease (C) and cell morphology, cellular function, and maintenance, reproductive system development and function (D). Differentially expressed genes are highlighted in red when upregulated or in green when downregulated in oocytes of bacteria‐infused heifers compared to vehicle‐infused heifers

Gene network analysis of oocytes collected at Day 4 predicted an enrichment of 23 gene networks among DEGs in bacteria‐infused heifers compared to vehicle‐infused heifers (Table 3 and Table S10), including (a) Cell Morphology, Cell‐To‐Cell Signaling and Interaction, Connective Tissue Development and Function (Figure 5A), (b) Antimicrobial Response, Connective Tissue Development and Function, Inflammatory Response (Figure 5B), and (c) Cellular Development, Cellular Movement, Neurological Disease. Enrichment of 25 gene networks among DEGs was identified in oocytes collected at Day 60 from bacteria‐infused heifers compared to vehicle‐infused heifers (Table 3 and Table S11), including (a) Dental Disease, Developmental Disorder, Gastrointestinal Disease (Figure 5C), (b) Cell Morphology, Cellular Function and Maintenance, Reproductive System Development and Function (Figure 5D), and (c) Cell Morphology, Cellular Development, Cellular Movement. Enriched gene networks identified in oocytes within a treatment group comparing days of collection are shown in Tables S12 and S13.

**TABLE 3:** Gene networks enriched among differentially expressed genes of oocytes collected from bacteria infused heifers compared to vehicle infused heifers at Day 4 and Day 60

Gene network ^a^	Network Score^b^	Differentially expressed genes^c^	
		Upregulated	Downregulated
Day 4			
Cell Morphology, Cell‐To‐Cell Signaling and Interaction, Connective Tissue Development and Function	36	*HOXA2*, *KMT2E*, *MFAP4*,	*AIF1L*, *C1QC*, *CD1D*, *CD37*, *CSRP3*, *CTSS*, *GAS1*, *GLRX2*, *IFITM1*, *KRT1*, *KRT18*, *KRT72*, *KRT8*, *LUM*, *MAF*, *SLC39A8*, *TAGLN*, *TGFB1*, *TYROBP*
Antimicrobial Response, Connective Tissue Development and Function, Inflammatory Response	34	*CHRNA5*, *LRCH3*, *MEFV*, *TG*	*BMP2*, *BMPR1B*, *CDC42EP1*, *COL1A2*, *ID1*, *IFNAR2*, *IRF2*, *ISG15*, *LTC4S*, *MARCH3*, *OAS1*, *P3H2*, *RSAD2*, *SMAD9*, *TCIM*, *TREX1*, *USP18*
Cellular Development, Cellular Movement, Neurological Disease	32	*AKT1S1*, *ARHGAP35*, *C8B*, *DLC1*, *ST8SIA2*, *STX17*	*AHSG*, *AMIGO2*, *ANKRD1*, *DNER*, *GPNMB*, *NDNF*, *NTS*, *RND3*, *RNF125*, *SIRPA*, *ST8SIA4*, *TLR6*, *TNFAIP6*, *YOD1*
Cellular Movement, Hematological System Development and Function, Immune Cell Trafficking	28	*APLF*, *CERS5*, *CHD1*, *MET*, *POLR2K*, *WDR75*	*ANXA2*, *C5AR1*, *CCL2*, *CCR5*, *GPRC5B*, *INO80B*, *NDP*, *PALMD*, *PCDHGC3*, *RAC2*, *S100A4*, *SPTA1*
Developmental Disorder, Hereditary Disorder, Neurological Disease	26	*AMOTL2*, *COL6A6*, *MSLN*, *NID1*, *OLR1*, *SIM2*	*CCN1*, *CCN2*, *CD68*, *COL1A1*, *IFI44L*, *PLD2*, *POSTN*, *SERPINE1*, *SNAI2*, *TMEM204*, *VCAN*
**Day 60**			
Dental Disease, Developmental Disorder, Gastrointestinal Disease	42	*CCT6A*, *CSRP3*, *EID2*, *EMX2*, *ENPP3*, *F11*, *FOXL2*, *GPC1*, *HSD3B2*, *IPO8*, *KIF21A*, *KLK4*, *KRT8*, *LGALS3BP*, *MAGED2*, *MYOF*, *OXT*, *QRSL1*, *RTN4*, *TUBA1A*, *ZBTB38*, *ZNF484*	*ABRA*, *ODAPH*, *ST14*
Cell Morphology, Cellular Function and Maintenance, Reproductive System Development and Function	40	*ARHGAP6*, *ASB9*, *C1S*, *CHRNA10*, *CHRNA9*, *DHRS7*, *DIPK2A*, *FOXP1*, *HOPX*, *LAPTM4B*, *MLC1*, *PCDHGC3*, *PROS1*, *PTX3*, *RHOQ*, *RRAGB*, *SERPING1*, *SERPINH1*, *SLC38A2*, *TNFRSF10A*, *UPK1B*	*CDH13*, *DNER*, *MYH3*
Cell Morphology, Cellular Development, Cellular Movement	35	*BCAM*, *CD151*, *COL1A1*, *DEPDC1*, *FBN2*, *IL27RA*, *INSL3*, *ITGA2*, *JAM2*, *MATN2*, *MFGE8*, *MKX*, *PEPD*, *PLEK*, *PLPP3*, *POSTN*, *RASGRP3*, *SH3BGRL*, *TNS1*, *VEGFD*, *VNN1*, *WDR75*	
Cell Signaling, Dermatological Diseases and Conditions, Immunological Disease	35	*ARHGAP45*, *CD99*, *G0S2*, *GLMP*, *HIVEP2*, *HSD17B7*, *IFI27*, *IFI6*, *IFNW1*, *IRF9*, *ISG15*, *MX1*, *OAS1*, *RNASEL*, *RSAD2*, *TCIM*, *TNFSF18*, *TRANK1*, *UBA7*, *USP18*, *VSNL1*	*IL2RG*
Cell‐To‐Cell Signaling and Interaction, Connective Tissue Development and Function, Skeletal and Muscular System Development and Function	33	*CCNT2*, *FADS2*, *FAM20C*, *FRK*, *H2AFY2*, *H2AFZ*, *HMGN5*, *HPSE*, *IFIT5*, *NMI*, *PARP10*, *PLOD2*, *SDC4*, *SPARCL1*, *STRA6*, *TDG*, *TGM2*, *THBS2*, *TNFAIP6*, *TTC27*, *ZNF189*	

^a^ Enriched gene networks determined by Ingenuity Pathway Analysis using differentially expressed genes only.

^b^ Network score is derived from a *P* value and indicates the likelihood of the genes in a network being found together due to random chance. A network score of 2 or greater gives a 99% confidence the network and genes not being generated by random chance alone.

^c^ Genes are differentially expressed (log_2_ fold change ≥ 2 or ≤ −2 and a *P* ≤ .05) in oocytes collected from bacteria infused heifers compared to control infused heifers at Day 4 or Day 60 relative to infusion.

Analysis of oocytes from bacteria‐infused heifers at Day 4 compared to vehicle‐infused heifers predicted a total of 232 possible upstream regulators of DEGs with a significant z‐score ≥ 2 or ≤ −2 (Table S14). Predicted upstream regulators of DEGs include LPS, TGFβ1, STAT3, and PRL (Table 4). A total of 122 predicted upstream regulators of DEGs were identified in oocytes collected from bacteria‐infused heifers compared to vehicle‐infused heifers at Day 60 (Table S15). Predicted upstream regulators of DEGs include chorionic gonadotropin, PRL, and IFNα2 (Table 4). A total of 858 predicted upstream regulators of DEGs were identified in oocytes collected from vehicle‐infused heifers at Day 60 compare to Day 4 (Table S16), including beta‐estradiol, FSH, LH dihydrotestosterone, prostaglandin E2, and progesterone. Only 3 predicted upstream regulators of DEGs were identified in oocytes collected from bacteria‐infused heifers at Day 60 compared to Day 4, including cytokine signaling, camptothecin, and genistein.

**TABLE 4:** Predicted upstream regulators of differentially expressed genes in oocytes collected from bacteria infused heifers compared to vehicle infused heifers at Day 4 and Day 60

Upstream regulator	*Z*‐score	Target DEGs
Day 4		
Lipopolysaccharide	−4.993	*ACTA2, ANGPTL4, APOE, ATF3, BMP2, C5AR1, CAVIN1, CCL2, CCN1, CCN2, CCNE1, CCR5, CD14, CD1D, CD37, CD68, CD9, CEBPD, CITED2, CLDN5, COL1A1, COL1A2, CSPG4, CXCL16, EGR1, FOSB, GAS1, GMFG, GSN, IFI44L, IFIT1, IFIT5, IFITM1, IGFBP5, IL9, IRF2, ISG15, JUNB, LITAF, LTC4S, MAF, MAOA, MEFV, MET, MX1, NID1, NTS, OAS1, OLR1, PARP10, PLAT, PLG, RGCC, RND3, RSAD2, SDC1, SERPINC1, SERPINE1, SIRPA, SLC39A8, TCIM, TGFB1, THBS2, TIMP3, TLR6, TNFAIP3, TNFAIP6, TNFRSF1B, TP63, TREX1, TYROBP, USP18, VCAN*
TGFB1	−4.692	*ACTA2, AIF1L, AMOTL2, ANGPTL4, ANKRD1, ANXA2, APOE, ARHGAP35, BMP2, C1QC, C5AR1, CCL2, CCN1, CCN2, CCNE1, CCR5, CD14, CD68, CITED2, COL1A1, COL1A2, CSPG4, CTSH, CTSS, EGR1, ELF4, FCGR1A, FOSB, GAS1, GLRX2, GPRC5B, GSN, HNRNPDL, HOXA2, ID1, IGFBP5, IL9, JUNB, KMT2E, KRT18, KRT8, LITAF, LTC4S, MAF, MAOA, MEFV, MET, MFAP4, NEDD9, OLR1, PLAT, POSTN, RAD1, RGCC, RSAD2, S100A4, SDC1, SERPINE1, SLC39A8, SNAI2, TAGLN, TG, TGFB1, THBS2, TIMP3, TNFAIP3, TNFAIP6, TNFRSF12A, VCAN*
PRL	−3.818	*ANXA2, CCL2, CEBPD, CLDN5, COL1A1, COL1A2, CTSH, CTSS, EGR1, GPNMB, HERC6, ID1, IFI44L, IFIT1, IFIT5, IFITM1, IGFBP5, ISG15, KRT72, OAS1, PARP10, PLAT, RSAD2, SDC1, TGFB1, TP63, USP18*
STAT3	−2.292	*ACTA2, AHSG, ANGPTL4, C5AR1, CCL2, CCN2, CCNE1, CCR5, CD9, CEBPD, COL1A1, COL1A2, EGR1, FCGR1A, HERC6, ID1, IFIT1, IFIT5, IFITM1, IGFBP5, IL9, ISG15, JUNB, MAF, MAP2, MX1, OAS1, RSAD2, SERPINE1, SMAD9, SNAI2, TAGLN, TGFB1, TNFRSF1B, USP18, VCAN*
IFNG	−3.683	*ACTA2, AIF1L, ANGPTL4, ATF3, C1QC, C5AR1, CCL2, CCN2, CCR5, CD14, CD1D, CD68, CEBPD, COL1A1, COL1A2, CTSH, CTSS, CXCL16, EGR1, FCGR1A, FOSB, GPRC5B, HERC6, ID1, IFI44L, IFIT1, IFIT5, IFITM1, IL9, IRF2, ISG15, JUNB,MEFV, MX1, NEDD9, OAS1, RAC2, RGCC, RSAD2, SDC1, SERPINE1, SNAI2, TG, TGFB1, TIMP3, TLR6, TNFAIP6, TNFRSF12A, TNFRSF1B, TP63, TYROBP, UBA2, USP18*
Day 60		
Chorionic gonadotropin complex	2.993	*BTC, CCND2, CLU, CYP19A1, EGR1, ENPP3, FOS, G0S2, GPC1, HPSE, HSD17B7, HSD3B2, IL33, LDLR, LGALS3BP, OXT, OXTR, PLPP3, PTX3, SDC4, ST8SIA4, TNFAIP6*
PRL	3.893	*CLU, COL1A1, EGR1, ERBB3, FOS, HERC6, HSD17B7, IFI6, IFIT5, IRF9, ISG15, MFGE8, OAS1, OXT, PARP10, PDK4, RSAD2, SCP2, USP18*
OSM	2.708	*BTC, C1S, CCND2, COL1A1, CYP19A1, DHRS7, DHX9, EGR1, ERBB3, FOS, HLA‐B, IL33, IRF9, KLF10, KRT8, LDLR, MAGED2, MAOA, MX1, OAS1, PEPD, PTGES, SAA1, SERPING1, TGM2, TPBG, TUBA1A, UAP1*
IFNL1	3.26	*HERC6, HLA‐B, IFI6, IFIT5, IRF9, ISG15, LGALS3BP, MX1, OAS1, RSAD2, USP18*
IFNA2	3.58	*C1S, HERC6, HLA‐B, IFI6, IFIT5, IRF9, ISG15, LAPTM4B, LGALS3BP, MX1, OAS1, RSAD2, TGM2, THEMIS2, TNFRSF1A, UBA7, USP18*

Note

A complete list of predicted upstream regulators can be found in Table S13 and S14.

## 4 DISCUSSION

Uterine infections, including endometritis, are common amongst postpartum dairy cattle, and subsequent infertility persists after the resolution of clinical symptoms.^24^ The mechanisms by which infertility persists in cattle following infection are unclear, but likely encompass perturbation in endocrine signaling, uterine health, and potentially oocyte quality. Oocytes collected from cows with spontaneous or induced mastitis have lower developmental competence compared to healthy herd mates.^14^, ^25^ Approximately 60 days following the resolution of spontaneous metritis, granulosa cell transcriptome is altered, suggesting a persistent effect of infection on the follicle which likely mediates changes in oocyte quality.^10^ In parallel, oocytes collected from cows with induced endometritis (using the model described here) have a reduced capacity to reach the morula stage of development following in vitro fertilization and embryo culture.^26^ Specifically, oocytes collected from cows infused with bacteria had a 32.5% reduction in the rate in which cleaved oocytes developed to morulae compared to control cows. Interestingly, developmental capacity of oocytes was negatively correlated to endometrial expression of *IL6* following intrauterine infusion. The study presented herein aimed to evaluate the impact of induced endometritis on oocyte transcriptome in virgin dairy heifers to better understand the underlying causes of uterine infection‐associated infertility. We found changes in the transcriptome of oocytes collected 4 and 60 days after intrauterine bacterial infusion. Notably, we found 452 DEGs at Day 4 and 539 DEGs at Day 60 in bacteria‐infused heifers. Interestingly, the changes to oocyte transcriptome shared only 42 DEGs at both Day 4 and Day 60, the majority of which displayed contrasting expression patterns. Predicted upstream regulators of DEGs in bacteria‐infused heifers at Day 4 included LPS, signal transducer, and activator of transcription 3 (STAT3) and interferon gamma (IFNγ), whereas predicted upstream regulators at Day 60 were different from those predicted at Day 4 and included chorionic gonadotropin (hCG) and prolactin (PRL). To our surprise 3,444 DEGs were identified in oocytes collected at Day 60 compared to Day 4 in vehicle‐infused heifers, suggesting considerable modification of oocyte transcriptome during the experimental period. This large difference in DEG is likely a reflection of the considerable change in serum progesterone observed between Day 4 and 60. This difference in serum progesterone between Day 4 and Day 60 is due to the use of exogenous progesterone required for disease induction at the beginning of the experiment when oocyte was collected at Day 4, and the absence of estrous synchronization when oocytes were collected at Day 60. Heatmaps (Figure 3) helped illustrate the clear separation of DEGs for oocytes between both infusion status, and day of collection. Interestingly, we did not observe an effect of bacterial infusion on serum progesterone, and only one heifer in each treatment group had serum progesterone below 1 ng/mL at Day 60, indicative of either estrus or anestrus.^27^ However, this degree of transcriptional change observed in oocyte collected from vehicle‐infused heifers over time was not evident in oocytes collected from bacteria‐infused heifers, with only 178 DEGs identified, which suggests that bacterial infusion hinders normal transcriptional modulation of growing oocytes.

We used virgin Holstein heifers to separate the effects of uterine infection from other potential confounding factors associated with lactation, negative energy balance or postpartum diseases, which are all common in dairy cows.^28^, ^29^ Based on the presence of pus in the uterus and vaginal mucus, and increased bacterial load in the vagina, intrauterine infusion of pathogenic bacteria induced clinical endometritis within 1 week of treatment, as reported previously.^8^ One week after infusion of bacteria, both treatment groups had comparable vaginal mucus scores, however, it is worth noting that vehicle‐infused heifers began to display elevated vaginal mucus scores that may be associated with the multiple and continued manipulation of the reproductive tract and likely reflects vaginitis or cervicitis, and not endometritis. A strength of this experimental model is the use of specific pathogenic strains of *E coli* and *T pyogenes* isolated from cows with uterine infection.^16^, ^17^ Thereby, these heifers represent a novel model to study the impact of endometritis on fertility, specifically the oocyte, in the absence of other confounding conditions common to the postpartum dairy cow.

All oocytes were collected from 2 to 8 mm diameter antral follicles. Development from the primordial to preovulatory follicle takes approximately 120 to 180 days in cattle, with the antrum forming during the last 42 days.^15^ Therefore, on Day 4 after bacterial infusion, the follicles would contain oocytes that had completed their initial growth and arrested at prophase of meiosis I at the time of intrauterine infusion. However, on Day 60, these follicles would have been secondary follicles containing growing oocytes at the time of intrauterine infusion. Additionally, oocytes collected at Day 4 were under the influence of exogenous progesterone and developed under the regulation of an estrus synchronization protocol using prostaglandin and GnRH, which likely have an effect on the capacity of the follicle to grow and mature under physiological concentrations of gonadotropins, and simultaneously impacting oocyte quality.^30^ Conversely, follicles aspirated at Day 60 developed without the use of exogenous hormones. The difference in hormonal exposure of follicles aspirated at Day 4 and Day 60 may be the cause of the large transcriptional differences observed between oocytes of vehicle‐infused heifers collected at these two time points. However, the number of DEGs observed in oocytes of vehicle‐infused heifers was much greater in oocytes collected from bacteria‐infused heifers, which suggests that bacterial infusion may inhibit transcription of growing oocytes in secondary follicles from Day 4 to Day 60. As the oocyte grows within the pre‐antral follicle, transcription and translation activity accumulates maternal mRNAs and proteins that are required for early embryonic development, prior to activation of the newly formed embryonic genome (reviewed in Ref. [31]). Once oocyte growth is completed during the early antral phase, and the oocyte reaches a size of approximately 120 μm, transcriptional activity ceases.^32^ Metaphase II oocytes have a lack of transcriptional activity, increased RNA instability, and increased RNA utilization and degradation, which results in reduced transcript abundance when compared to prophase oocytes.^33^, ^34^, ^35^ The increased transcription of the growing oocyte suggests a window of susceptibility of the pre‐antral follicle to stressors, including uterine infection, while oocytes that have completed transcription may be susceptible to increased transcript degradation. This may be in part the reason that so few DEG were similar in oocytes of bacteria‐infused cows at Day 4 and Day 60 compared to vehicle‐infused cows. It could be postulated that oocytes of various developmental stages (antral versus secondary in this case) are differentially susceptible to the effects of uterine infection; perhaps active transcription is modulated in growing oocytes, while transcript degradation is affected in full‐grown oocytes? In addition, fertility of cattle that are subjected to the stress of temporary feed restriction for 50 days display long‐term consequences on fertility.^36^, ^37^ Immediately following feed restriction and the resumption of normal caloric intake there is no difference in the fertility of cows, however, 43 days following resumption of normal feeding, feed restricted cows have higher fertility than their control counterparts. This implies that specific stages of follicle development are susceptible to stressors, and that the negative consequences of these perturbations are not observed until later.

It is remarkable that infusion of bacteria into the uterus alters the transcriptome of oocytes in ovarian follicles. However, LPS accumulates in the follicular fluid of cows with uterine infection, with concentrations higher than peripheral blood, and follicular LPS concentrations correlate with the degree of uterine inflammation.^9^ In addition, inflammatory mediators including LPS and IL‐8, are increased in the follicular fluid of cows with resolved metritis, and the transcriptome of granulosa cells is altered compared to healthy herd mates after the resolution of disease.^10^ These phenomena likely alter the follicle microenvironment and may compromise oocyte quality. Experiments using in vitro culture highlight the ability of granulosa cells to increase production of inflammatory mediators in response to bacterial components via the Toll‐like receptor pathway.^12^ Many of the inflammatory mediators increased in granulosa cells following exposure to bacterial components also have a role in the normal physiological processes of the ovary, including follicle maturation, ovulation, and the communication between cumulus cells and oocyte.^38^, ^39^, ^40^ Altering the normal expression of these key inflammatory molecules required for maturation of the follicle and oocyte may alter the quality of the oocyte or physiology of the ovary.

Ingenuity Pathway Analysis identified several canonical pathways in oocytes affected by intrauterine bacterial infusion. Canonical pathways are generalized pathways, common across organisms, tissues, and cells. At Day 4, bacterial infusion inhibited oocyte IFN signaling due to the downregulation of *IFIT1*, *IFITM1*, *IFNAR2*, *ISG15*, *MX1*, and *OAS1*. The genes *ISG15*, *MX1* and *OAS1* are IFN stimulated genes, which are upregulated in peripheral blood of cattle that are pregnant in response to IFN produced by the conceptus.^41^ Conversely, these three genes were upregulated at Day 60 in oocytes from bacteria‐infused heifers. The relevance of differential expression of these genes here in the oocyte is unclear as the these cows were not pregnant and the conceptus produces IFN‐tau at approximately Day 15 of pregnancy resulting in the increased expression of these genes in peripheral blood cells by Day 18 of pregnancy.^42^ Perhaps exposure to bacterial infection alters the future capacity of the conceptus to produce IFN‐tau, which is a requirement for corpus luteum maintenance in cattle? Perturbing IFN‐tau production by the conceptus would have significant impacts on the ability to maintain pregnancy. However, the concentration of uterine fluid IFN‐tau or expression of *IFNT* by the embryo do not differ on Day 15 of pregnancy between bacteria‐infused and control cows 145 days after uterine infusion.^26^ The canonical pathway TEK‐kinase signaling was increased in oocytes of bacteria‐infused heifers at Day 60. TEK‐kinases are a large family of non‐receptor tyrosine kinases that have a central role in phospholipase‐C activation and Ca^2+^ mobilization,^43^ processes key to fertilization and early embryonic development.^44^ Due to the large number of molecules involved in TEK signaling it is unclear if modulating this pathway in oocytes would have a major defect in the capacity of oocyte to undergo fertilization and embryonic development, however, SRC‐kinases which are recruited by TEK‐kinases are integral in fertilization, as such alterations to TEK‐kinase activity may have significant consequences on fertility.^45^

A total of 232 upstream regulators were predicted for the 452 DEGs of oocytes collected from bacteria‐infused heifers at Day 4. The majority of these 232 upstream regulators were predicted to be inactivated (only 40 were predicted to be activated). We had previously hypothesized that inflammatory mediators would be upregulated in oocytes collected from bacteria‐infused heifers on Day 4; however, upstream regulators including LPS, STAT3, IL‐1β, IL‐6, and TLR4 were predicted to be inactivated in oocytes of bacteria‐infused heifers at Day 4. Conversely, upstream regulators including TNF, LPS, interferon, IL‐1β, and IL‐6 were activated in oocytes of bacteria‐infused heifers at Day 60 long after the resolution of uterine infection. This was a surprising finding to us and may imply that oocytes of antral follicles at Day 4 that were exposed to active infection may attempt to minimize the effects of bacterial infection by downregulating inflammatory responses. Conversely, oocytes of smaller, growing follicles that would be harvested at Day 60 may be more susceptible to the negative effects of uterine infection and increase expression of inflammatory mediators, as observed; however, this hypothesis will require further investigation. Upstream regulators of the 3,444 DEGs in oocytes collected from control heifers at Day 4 and Day 60 primarily included endocrine signaling molecules such as estradiol, estrogen receptor (ESR2), hCG, LH, FSH, IGF‐I, dihydrotestosterone, progesterone, EGFR, and prostaglandin E_2_. This fits with our hypothesis that oocytes at Day 4 and Day 60 alter gene expression due to the endocrine environment in which they develop, with oocytes at Day 60 developing under natural endocrine signals, and oocytes at Day 4 developing in the presence of exogenous hormones. Conversely, only three upstream regulators of DEGs were predicted for oocytes collected from bacteria‐infused heifers when comparing Day 4 to Day 60. Both camptothecin and genistein, identified as upstream regulators in bacteria‐infused heifers, inhibit DNA topoisomerase and likely reduce transcription and cell division.^46^, ^47^ This reiterates to us that bacterial infusion prevents normal growth and development of oocytes, regardless of the endocrine environment to which they are exposed and may suggest that little could be done to remedy the negative consequences of bacterial infection on oocyte transcriptome.

In conclusion, we found that intrauterine bacterial infusion altered the oocyte transcriptome both in the short term, 4 days, and over a longer period of 60 days. Interestingly, genes and pathways associated with inflammation were downregulated in oocytes collected 4 days after bacterial infusion, while many of the same markers and pathways were activated in oocytes collected on Day 60. In addition, exposure of different follicle stages to uterine infection resulted in unique changes to the oocyte transcriptome, while bacterial infusion prevented changes to the oocyte transcriptome associated with the use of exogenous progesterone in our model. Together the findings imply that there are effects on the transcriptome of oocytes, whether that infection is during the early or antral stages of follicle development. The changes observed in the transcriptome of oocytes may be indicative of the reduced developmental competence of oocytes following uterine infusion of bacteria. Future work is required to further explore how these infection‐induced changes to the transcriptome of oocytes cause infertility in cattle.

## CONFLICT OF INTEREST

The authors have nothing to declare.

## AUTHOR CONTRIBUTIONS

J. J. Bromfield and I. M. Sheldon designed research; R. L. Piersanti, J. Block, Z. Ma, K. C. Jeong, J. E. P. Santos, I. M. Sheldon, and J. J. Bromfield performed research; R. L. Piersanti, F. Yu, I. M. Sheldon, and J. J. Bromfield analyzed data; R. L. Piersanti, J. J. Bromfield, and I. M. Sheldon wrote the paper.

## ETHICAL STATEMENT

All animal procedures were approved by the University of Florida Institutional Animal Care and Use Committee (protocol 201508 884).

## Supporting information

Supplementary Material

Supplementary Material

## References

[1] Sheldon IM , Cronin JG , Bromfield JJ . Tolerance and innate immunity shape the development of postpartum uterine disease and the impact of endometritis in dairy cattle. Annu Rev Anim Biosci. 2019;7:361‐384. 30359085 10.1146/annurev-animal-020518-115227 PMC6450715

[2] LeBlanc SJ , Duffield TF , Leslie KE , et al. Defining and diagnosing postpartum clinical endometritis and its impact on reproductive performance in dairy cows. J Dairy Sci. 2002;85:2223‐2236. 12362455 10.3168/jds.S0022-0302(02)74302-6

[3] Gilbert RO , Shin ST , Guard CL , Erb HN , Frajblat M . Prevalence of endometritis and its effects on reproductive performance of dairy cows. Theriogenology. 2005;64:1879‐1888. 15961149 10.1016/j.theriogenology.2005.04.022

[4] Borsberry S , Dobson H . Periparturient diseases and their effect on reproductive performance in five dairy herds. Vet Rec. 1989;124:217‐219. 2929110 10.1136/vr.124.9.217

[5] Bromfield JJ , Santos JE , Block J , Williams RS , Sheldon IM . Physiology and endocrinology symposium: uterine infection: linking infection and innate immunity with infertility in the high‐producing dairy cow. J Anim Sci. 2015;93:2021‐2033. 26020298 10.2527/jas.2014-8496

[6] Griffin JF , Hartigan PJ , Nunn WR . Non‐specific uterine infection and bovine fertility. I. Infection patterns and endometritis during the first seven weeks post‐partum. Theriogenology. 1974;1:91‐106. 4619812 10.1016/0093-691x(74)90052-1

[7] Sheldon IM , Noakes DE , Rycroft AN , Pfeiffer DU , Dobson H . Influence of uterine bacterial contamination after parturition on ovarian dominant follicle selection and follicle growth and function in cattle. Reproduction. 2002;123:837‐845. 12052238

[8] Piersanti RL , Zimpel R , Molinari PCC , et al. A model of clinical endometritis in Holstein heifers using pathogenic Escherichia coli and Trueperella pyogenes. J Dairy Sci. 2019;102:2686‐2697. 30692014 10.3168/jds.2018-15595 PMC6445275

[9] Herath S , Williams EJ , Lilly ST , et al. Ovarian follicular cells have innate immune capabilities that modulate their endocrine function. Reproduction. 2007;134:683‐693. 17965259 10.1530/REP-07-0229 PMC2735812

[10] Piersanti RL , Horlock AD , Block J , Santos JEP , Sheldon IM , Bromfield JJ . Persistent effects on bovine granulosa cell transcriptome after resolution of uterine disease. Reproduction. 2019;158:35‐46. 30933928 10.1530/REP-19-0037 PMC6773536

[11] Williams EJ , Fischer DP , Noakes DE , et al. The relationship between uterine pathogen growth density and ovarian function in the postpartum dairy cow. Theriogenology. 2007;68:549‐559. 17574659 10.1016/j.theriogenology.2007.04.056 PMC2702080

[12] Bromfield JJ , Sheldon IM . Lipopolysaccharide initiates inflammation in bovine granulosa cells via the TLR4 pathway and perturbs oocyte meiotic progression in vitro. Endocrinology. 2011;152:5029‐5040. 21990308 10.1210/en.2011-1124 PMC3428914

[13] Soto P , Natzke RP , Hansen PJ . Identification of possible mediators of embryonic mortality caused by mastitis: actions of lipopolysaccharide, prostaglandin F2alpha, and the nitric oxide generator, sodium nitroprusside dihydrate, on oocyte maturation and embryonic development in cattle. Am J Reprod Immunol. 2003;50:263‐272. 14629032 10.1034/j.1600-0897.2003.00085.x

[14] Roth Z , Dvir A , Kalo D , et al. Naturally occurring mastitis disrupts developmental competence of bovine oocytes. J Dairy Sci. 2013;96:6499‐6505. 23957998 10.3168/jds.2013-6903

[15] Lussier JG , Matton P , Dufour JJ . Growth rates of follicles in the ovary of the cow. J Reprod Fertil. 1987;81:301‐307. 3430454 10.1530/jrf.0.0810301

[16] Goldstone RJ , Amos M , Talbot R , et al. Draft genome sequence of Trueperella pyogenes, isolated from the infected uterus of a postpartum cow with metritis. Genome Announcements. 2014;2:e00194‐00114. 24762932 10.1128/genomeA.00194-14 PMC3999489

[17] Goldstone RJ , Talbot R , Schuberth HJ , Sandra O , Sheldon IM , Smith DG . Draft genome sequence of Escherichia coli MS499, isolated from the infected uterus of a postpartum cow with metritis. Genome Announcements. 2014;2:e00217‐00214. 24994791 10.1128/genomeA.00217-14 PMC4081991

[18] Sheldon IM , Cronin J , Goetze L , Donofrio G , Schuberth HJ . Defining postpartum uterine disease and the mechanisms of infection and immunity in the female reproductive tract in cattle. Biol Reprod. 2009;81:1025‐1032. 19439727 10.1095/biolreprod.109.077370 PMC2784443

[19] Martin M . Cutadapt removes adapter sequences from high‐throughput sequencing reads. EMBnet J. 2011;17:10‐12.

[20] Langmead B , Salzberg SL . Fast gapped‐read alignment with Bowtie 2. Nat. Methods. 2012;9:357‐359. 22388286 10.1038/nmeth.1923 PMC3322381

[21] Yao JQ , Yu F . DEB: a web interface for RNA‐seq digital gene expression analysis. Bioinformation. 2011;7:44‐45. 21904439 10.6026/97320630007044 PMC3163933

[22] Babicki S , Arndt D , Marcu A , et al. Heatmapper: web‐enabled heat mapping for all. Nucleic Acids Res. 2016;44:W147‐153. 27190236 10.1093/nar/gkw419 PMC4987948

[23] Metsalu T , Vilo J . ClustVis: a web tool for visualizing clustering of multivariate data using Principal Component Analysis and heatmap. Nucleic Acids Res. 2015;43:W566‐570. 25969447 10.1093/nar/gkv468 PMC4489295

[24] Ribeiro ES , Gomes G , Greco LF , et al. Carryover effect of postpartum inflammatory diseases on developmental biology and fertility in lactating dairy cows. J. Dairy Sci. 2016;99:2201‐2220. 26723113 10.3168/jds.2015-10337

[25] Asaf S , Leitner G , Furman O , et al. Effects of Escherichia coli‐ and Staphylococcus aureus‐induced mastitis in lactating cows on oocyte developmental competence. Reproduction. 2014;147:33‐43. 24129150 10.1530/REP-13-0383

[26] Dickson MJ , Piersanti RL , Ramirez‐Hernandez R , et al. Experimentally induced endometritis impairs the developmental capacity of bovine oocytes†. Biol Reprod. 2020 10.1093/biolre/ioaa069. [Epub ahead of print]. PMC7442778 32401311

[27] Stevenson JS , Pursley JR , Garverick HA , et al. Treatment of cycling and noncycling lactating dairy cows with progesterone during Ovsynch. J Dairy Sci. 2006;89:2567‐2578. 16772576 10.3168/jds.S0022-0302(06)72333-5

[28] Girard A , Dufort I , Sirard MA . The effect of energy balance on the transcriptome of bovine granulosa cells at 60 days postpartum. Theriogenology. 2015;84(1350–1361):e1356. 10.1016/j.theriogenology.2015.07.015 26316219

[29] Wathes DC , Cheng Z , Chowdhury W , et al. Negative energy balance alters global gene expression and immune responses in the uterus of postpartum dairy cows. Physiol Genomics. 2009;39:1‐13. 19567787 10.1152/physiolgenomics.00064.2009 PMC2747344

[30] Fricke PM , Carvalho PD , Lucy MC , et al. Effect of manipulating progesterone before timed artificial insemination on reproductive and endocrine parameters in seasonal‐calving, pasture‐based Holstein‐Friesian cows. J Dairy Sci. 2016;99:6780‐6792. 27320671 10.3168/jds.2016-11229

[31] Hyttel P , Viuff D , Fair T , et al. Ribosomal RNA gene expression and chromosome aberrations in bovine oocytes and preimplantation embryos. Reproduction. 2001;122:21‐30. 11425326

[32] Fair T , Hyttel P , Greve T . Bovine oocyte diameter in relation to maturational competence and transcriptional activity. Mol Reprod Dev. 1995;42:437‐442. 8607973 10.1002/mrd.1080420410

[33] Reyes JM , Chitwood JL , Ross PJ . RNA‐Seq profiling of single bovine oocyte transcript abundance and its modulation by cytoplasmic polyadenylation. Mol Reprod Dev. 2015;82:103‐114. 25560149 10.1002/mrd.22445 PMC4651626

[34] Vassalli JD , Huarte J , Belin D , et al. Regulated polyadenylation controls mRNA translation during meiotic maturation of mouse oocytes. Genes Dev. 1989;3:2163‐2171. 2483395 10.1101/gad.3.12b.2163

[35] Fair T , Carter F , Park S , Evans AC , Lonergan P . Global gene expression analysis during bovine oocyte in vitro maturation. Theriogenology. 2007;68(Suppl 1):S91‐97. 17512044 10.1016/j.theriogenology.2007.04.018

[36] Parr MH , Crowe MA , Lonergan P , Evans AC , Fair T , Diskin MG . The concurrent and carry over effects of long term changes in energy intake before insemination on pregnancy per artificial insemination in heifers. Anim Reprod Sci. 2015;157:87‐94. 25899522 10.1016/j.anireprosci.2015.03.019

[37] Britt JH , Cushman RA , Dechow CD , et al. Invited review: learning from the future‐A vision for dairy farms and cows in 2067. J Dairy Sci. 2018;101:3722‐3741. 29501340 10.3168/jds.2017-14025

[38] Richards JS , Russell DL , Ochsner S , et al. Novel signaling pathways that control ovarian follicular development, ovulation, and luteinization. Recent Prog Horm Res. 2002;57:195‐220. 12017544 10.1210/rp.57.1.195

[39] Shimada M , Hernandez‐Gonzalez I , Gonzalez‐Robanya I , Richards JS . Induced expression of pattern recognition receptors in cumulus oocyte complexes: novel evidence for innate immune‐like functions during ovulation. Mol Endocrinol. 2006;20:3228‐3239. 16931571 10.1210/me.2006-0194

[40] Liu Z , de Matos DG , Fan HY , Shimada M , Palmer S , Richards JS . Interleukin‐6: an autocrine regulator of the mouse cumulus cell‐oocyte complex expansion process. Endocrinology. 2009;150:3360‐3368. 19299453 10.1210/en.2008-1532 PMC2703543

[41] Pugliesi G , Miagawa BT , Paiva YN , Franca MR , Silva LA , Binelli M . Conceptus‐induced changes in the gene expression of blood immune cells and the ultrasound‐accessed luteal function in beef cattle: how early can we detect pregnancy? Biol Reprod. 2014;91:95. 25210129 10.1095/biolreprod.114.121525

[42] Gifford CA , Racicot K , Clark DS , et al. Regulation of interferon‐stimulated genes in peripheral blood leukocytes in pregnant and bred, nonpregnant dairy cows. J Dairy Sci. 2007;90:274‐280. 17183095 10.3168/jds.S0022-0302(07)72628-0

[43] Lin J , Weiss A . T cell receptor signalling. J Cell Sci. 2001;114:243‐244. 11148124 10.1242/jcs.114.2.243

[44] Whitaker M . Calcium at fertilization and in early development. Physiol Rev. 2006;86:25‐88. 16371595 10.1152/physrev.00023.2005 PMC3299562

[45] McGinnis LK , Carroll DJ , Kinsey WH . Protein tyrosine kinase signaling during oocyte maturation and fertilization. Mol Reprod Dev. 2011;78:831‐845. 21681843 10.1002/mrd.21326 PMC3186829

[46] Markovits J , Linassier C , Fosse P , et al. Inhibitory effects of the tyrosine kinase inhibitor genistein on mammalian DNA topoisomerase II. Cancer Res. 1989;49:5111‐5117. 2548712

[47] Pommier Y . Topoisomerase I inhibitors: camptothecins and beyond. Nat Rev Cancer. 2006;6:789‐802. 16990856 10.1038/nrc1977

