# Supplementary material for: Uterine infusion of bacteria alters the transcriptome of bovine oocytes
Source: FASEB Bioadv. 2020 Jul 16;2(8):506–20. doi: 10.1096/fba.2020-00029 (PMC7429353; doi:10.1096/fba.2020-00029)
Supplement: Supplementary file 1 — Supplementary Material [file FBA2-2-506-s001.pdf]

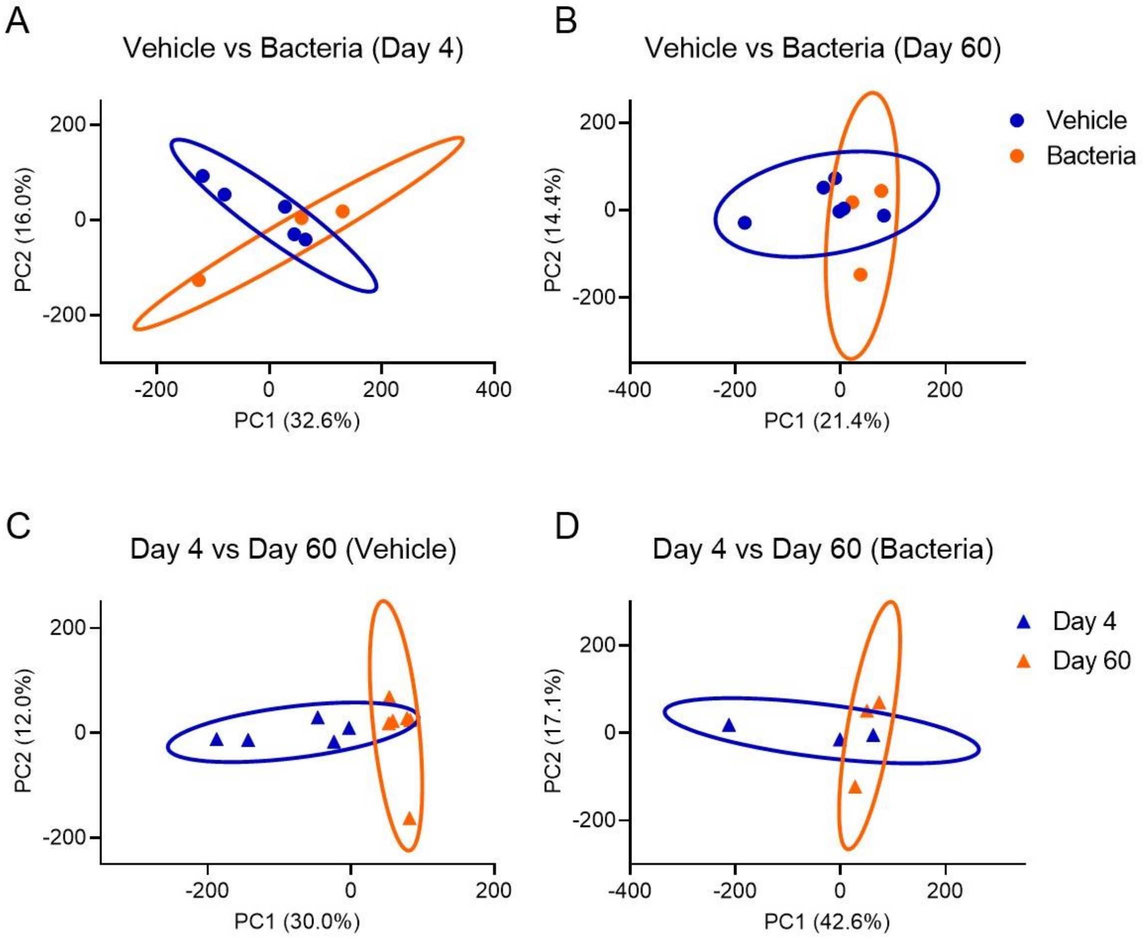

Supplemental Figure 1. Principal component analysis of oocyte transcripts identified following RNAseq.

Principal component analysis comparing RNAseq data of oocytes of vehicle-infused heifers (blue circle), and bacteria-infused heifers (orange circle) at day 4 (A) or day 60 (B). Analysis was also performed comparing oocytes collected at day 4 (blue triangle) to day 60 (orange triangle) from vehicle-infused heifers (C) or bacteria-infused heifers (D). Blue or orange lines denote the predicted clustering of samples dependent on treatment or day of collection, while axes describe the percent of variability associated with each sample; principal component (PC) 1 and PC2.
