# Supplementary material for: Uterine infusion of bacteria alters the transcriptome of bovine oocytes
Source: FASEB Bioadv. 2020 Jul 16;2(8):506–20. doi: 10.1096/fba.2020-00029 (PMC7429353; doi:10.1096/fba.2020-00029)
Supplement: Supplementary file 2 — Supplementary Material [file FBA2-2-506-s002.pdf]

Supplemental Table 1. Read mapping summary from RNAseq.

Day	Trt	Oocytes	RNA conc (pg/µL)	Raw reads	Cleaned reads	Mapped reads	Mapped reads (%)	Mapped transcripts
5	Veh	8	8022	68,339,022	68,339,021	26,610,968	38.9	16,080
5	Veh	2	233	65,241,582	65,241,582	19,835,333	30.4	16,464
5	Veh	7	149	67,451,076	67,451,075	21,668,946	32.1	16,638
5	Veh	7	409	72,540,014	72,540,013	25,773,241	35.5	17,848
5	Veh	4	5722	74,939,070	74,939,065	29,819,377	39.8	16,054
5	Bac	7	908	80,568,878	80,568,862	22,081,940	27.4	11,341
5	Bac	4	95	60,228,266	60,228,265	19,710,668	32.7	18,519
5	Bac	4	102	76,023,292	76,023,291	23,719,613	31.2	17,800
61	Veh	1	44	77,460,136	77,460,129	23,692,041	30.6	15,627
61	Veh	3	32	66,547,886	66,547,885	20,853,353	31.3	18,321
61	Veh	2	37	77,062,458	77,062,456	24,429,909	31.7	16,597
61	Veh	1	50	74,278,946	74,278,940	23,013,202	31.0	13,966
61	Veh	3	31	68,200,330	68,200,330	21,075,477	30.9	17,958
61	Veh	1	25	77,027,406	77,027,406	23,619,409	30.7	18,436
61	Bac	4	82	78,644,926	78,644,926	25,059,799	31.9	18,485
61	Bac	2	25	68,166,968	68,166,967	21,269,855	31.2	17,435
61	Bac	3	141	72,060,840	72,060,840	22,799,718	31.6	16,743
Avg		3.7	947	72,045,947	72,045,944	23,237,226	32.0	16,724
SEM		0.6	97	1,363,623	1,363,623	63,2280	0.8	450

Supplemental Table 2. High stringency differentially expressed genes in oocytes of bacteria infused heifers at day 4.

**Gene symbol**	**Accession number**	**$\mathbf{Log_2}$ fold change**	**Adjusted *P*-value**
ADAM9	XM_005210987.3	-7.20	0.067
C2H2orf88	NM_001046090.2	-6.98	0.006
FBN3	NM_174595.2	-7.91	0.016
KIAA1324	NM_001102204.1	-3.64	0.121
LOC104970629	XM_002687246.2	-5.88	0.064
NID1	NM_001205786.1	-3.58	0.073
PPP2R1B	NM_001303535.1	10.17	< 0.001
ST7L	NM_001113284.1	-6.44	0.012
STARD10	NM_001046157.1	-8.55	0.021
ZC3H12C	NM_001046118.2	-4.87	0.036

Genes are differentially expressed ($\log_2$ fold change ≥ 2 or ≤ -2 and an adjusted $P \leq 0.1$) in oocytes collected from bacteria infused heifers compared to heifers infused with vehicle medium at day 4 relative to infusion.

Supplemental Table 3. High stringency differentially expressed genes in oocytes of bacteria infused heifers at day 60.

Gene symbol	Accession number	Log_2 fold change	Adjusted *P*-value
ADAM22	NM_001191131.1	-8.20	0.019
AKAP2	NM_001193034.2	2.16	0.060
ARHGAP6	NM_001191354.1	3.05	0.123
BCDIN3D	NM_001075216.1	4.84	0.070
CCT6A	NM_001034542.2	2.08	0.001
CD58	XM_010803137.1	3.18	0.009
COL5A1	XM_015473738.1	7.00	< 0.001
CTHRC1	NM_001081536.2	7.93	0.033
CUX1	XM_010819623.1	3.45	0.009
CYP19A1	NM_174305.1	4.52	0.016
ETF1	XM_005209423.3	2.68	0.039
FXYD3	NM_001079646.1	7.26	0.148
GATA6	XM_002697727.2	2.95	0.005
H2AFZ	XM_010805947.2	2.50	0.005
HPSE	NM_174082.2	2.31	0.061
IL27RA	NM_001098028.1	2.34	0.025
INSL3	NM_174365.2	2.85	< 0.001
IPO8	NM_001206120.1	2.47	0.032
ISG20L2	NM_001046217.2	2.29	0.047
JAM2	NM_001083736.1	2.97	0.009
KLF10	NM_001168462.1	2.74	< 0.001
LAPTM4B	NM_205802.1	2.19	0.005
LOC101903564	XR_238481.3	7.98	0.079
LOC101907782	XR_233800.3	3.81	0.025
LOC104975788	XR_001501437.1	7.68	0.028
LOC616942	NM_001206459.1	5.00	< 0.001
LPL	NM_001075120.1	3.51	0.005
MAOA	NM_181014.2	3.50	0.070
MBTPS2	XM_010822145.2	3.24	0.068
MSANTD3	NM_001076019.1	-2.26	0.112
NAP1L5	NM_001077878.2	4.17	0.025
PCDHGA2	NM_001099368.2	4.27	0.119
PLOD2	NM_001101149.1	2.37	0.040
PPP1R3B	NM_001103247.1	3.92	0.006
PROS1	NM_174438.1	2.49	0.009
PSMA2	NM_001034662.2	2.19	0.025
RGS3	XM_015472689.1	4.03	0.116
RHOQ	NM_001205498.1	3.26	0.128

SERPINH1	NM_001046063.1	2.18	0.032
SMOC2	NM_001098134.1	2.10	0.026
TMEM246	NM_001102342.2	2.52	0.029
TPBG	XM_002690047.4	2.39	0.119
TTC31	XM_015473382.1	5.46	0.146
TYW3	NM_001015620.1	3.03	0.097
UPK1B	NM_174482.2	3.26	< 0.001
VSNL1	NM_174490.3	3.41	0.097
ZNF484	NM_001192027.1	3.28	0.005

Genes are differentially expressed ($\log_2$ fold change ≥ 2 or ≤ -2 and an adjusted $P \leq 0.1$) in oocytes collected from bacteria infused heifers compared to heifers infused with vehicle medium at day 60 relative to infusion.

Supplemental Table 4. Differentially expressed genes in oocytes of bacteria infused heifers at day 4.

**Gene symbol**	**Accession number**	**Log$_2$ fold change**	***P*-value**
AAGAB	NM_001130748.1	-3.41	< 0.001
ABCC5	NM_001033615.2	-4.09	0.052
ABHD6	NM_001102276.2	-6.74	0.003
ACSL6	XM_005228139.2	-2.42	0.055
ADAM9	XM_005210987.3	-7.20	< 0.001
ADORA1	NM_174473.4	-2.93	0.004
AFF3	XR_807235.2	-5.93	0.008
AIFM1	NM_001206040.1	-5.26	0.038
AKAP1	NM_001099145.2	-2.59	0.023
AKT1S1	XM_015473931.1	-3.03	0.022
AMOTL2	XM_010808773.2	4.19	0.027
ANAPC10	XM_015460185.1	4.63	0.047
ANGPTL1	XM_003586581.4	2.23	0.031
ANK3	XM_005202465.3	4.21	0.009
ANPEP	XR_001494405.1	5.50	0.029
AOX1	NM_001205858.1	-2.79	0.029
APAF1	XR_001494504.1	-5.19	0.030
APBB2	XM_005203868.3	-3.68	0.038
ARHGAP21	XM_015474132.1	4.43	0.031
ARL13B	XR_001494887.1	-3.38	0.031
ARPP19	NM_174030.2	-4.23	0.007
ASB12	XM_002694364.5	-5.41	0.007
ATG10	NM_001014926.1	-5.12	< 0.001
ATXN2	XR_001494914.1	-5.29	0.019
B4GALT2	NM_001099141.1	-4.98	0.008
BAI3	XM_003585994.3	7.85	0.024
BLCAP	XR_001494696.1	3.57	0.009
BMP4	XM_010816561.2	-4.41	0.040
BMP8A	XM_015458647.1	3.42	0.002
BOLA-DQA1	XR_001494179.1	5.48	0.050
BOLA-DRB2	XM_005215409.3	7.11	0.053
BSND	NM_001205724.1	4.60	0.049
C10H14orf39	XR_001494480.1	-3.40	0.031
C11H2orf73	XM_005206423.3	2.86	0.049
C14H8orf88	XM_015471688.1	4.55	0.040
C18H19orf73	XR_234819.3	-3.73	0.051
C19H17orf80	XM_010809076.1	-3.65	0.038
C23H6orf201	XM_015475560.1	3.27	0.024

C24H18orf42	NM_001034537.1	-2.85	0.034
C25H16orf45	XR_237843.2	-2.52	0.023
C2H2orf88	NM_001046090.2	-6.98	< 0.001
C3	XM_015460598.1	-3.36	0.015
C3H1orf226	XM_002698685.5	-3.45	0.042
CALML5	NM_001205716.1	-2.70	0.004
CASC3	NM_001192776.1	-2.29	0.022
CATSPER2	XM_010813121.2	-7.00	< 0.001
CATSPERD	NM_174514.2	2.51	0.013
CBY3	NM_001192095.1	-7.72	0.029
CCDC189	NM_001075944.1	-6.33	0.003
CCDC63	NM_001075819.1	-2.18	0.007
CCDC79	XM_010809248.2	2.78	0.017
CCP110	NM_001102248.1	-4.44	0.016
CCPG1	NM_001040479.1	-5.40	0.001
CCT6B	NM_001024493.1	-4.37	0.039
CDC27	XM_005219148.3	-3.33	0.020
CENPC	XM_002684584.4	-3.33	0.037
CENPE	XM_002690458.4	-2.11	0.025
CERS5	NM_001102085.1	4.32	0.019
CES4A	XM_005208172.3	-3.53	0.032
CHD1	XM_010803244.1	4.60	0.024
CHGB	NM_001075983.1	-4.37	0.031
CHL1	NM_174060.1	-4.52	< 0.001
CNTN5	NM_001102493.1	6.17	< 0.001
COPS7A	XM_010801462.2	5.15	0.010
COQ6	XR_814683.2	-7.21	0.032
CPEB4	XM_010819756.2	-5.13	0.054
CPXM2	NM_173984.3	-3.34	0.015
CRIP3	XR_807522.2	4.49	0.021
CTRB1	XR_001495407.1	4.23	0.046
CXCL13	NM_001080217.1	4.94	0.042
DAP3	NM_001076377.1	-4.82	0.011
DDX4	XM_015472696.1	5.83	0.043
DIABLO	XR_808071.2	10.09	0.005
DLGAP4	NM_001011672.2	-3.02	0.042
DNAH17	NM_001035094.2	-5.24	0.009
DNAH5	XR_236025.3	5.90	0.015
DNASE1L3	XM_002689290.4	2.10	0.042
DOCK8	XM_005228277.3	-2.78	0.035
DOPEY2	XM_005226412.2	-4.33	0.032

DRC7	XR_805447.2	-6.08	0.020
DTWD1	NM_001192782.1	-4.12	0.002
DUS4L	NM_001025325.2	-5.19	0.012
DUSP10	XM_002691722.4	-5.35	0.015
DYRK4	XR_815742.2	5.06	0.040
EEF1AKMT1	NM_001014907.1	-2.80	0.022
EFNA4	XM_005209116.3	4.92	0.019
ELAVL3	XM_010818385.2	3.25	0.052
ELP	XM_015461195.1	6.56	0.003
EML2	NM_176669.3	-3.35	0.043
ENOX1	NM_176872.1	-6.47	< 0.001
ERMARD	XM_005207046.3	-6.62	0.032
ERMARD	XR_001501437.1	-3.26	0.040
ERN2	NM_001075495.1	-4.61	0.004
EVC	XM_005210728.3	-4.01	0.034
EVI2B	XR_812676.2	-3.90	0.026
EXOC3L4	XM_002699804.4	2.44	0.031
EXPH5	NM_001166068.1	-5.61	0.033
EYA2	XM_002691937.4	-6.28	0.024
FAM13C	XM_015472358.1	-4.39	0.029
FAM163B	NM_174146.3	-6.63	0.002
FAM177A1	XM_005210684.3	-4.30	0.022
FAM186B	XM_003585774.2	-3.16	0.014
FAM19A3	XM_010803061.2	2.09	0.007
FAM234A	NM_001024689.3	-9.79	< 0.001
FAM71E1	XM_015459087.1	-4.83	0.021
FAM83H	NM_001040523.2	-5.74	0.027
FANCD2OS	XR_001501884.1	-2.54	0.033
FBN3	NM_174595.2	-7.91	< 0.001
FBXO16	XM_015459494.1	8.01	0.034
FDX1L	XM_003587989.3	-2.60	0.034
FGF12	XM_002697505.4	2.28	0.040
FNDC3B	XM_010803671.2	-6.51	0.014
FNDC8	XR_810452.2	3.96	0.014
FOXN3	NM_174475.3	-2.34	0.014
FOXN4	NM_001034385.2	-4.77	0.002
FOXP1	NM_001040490.2	-3.23	0.026
GABA	NM_175792.2	-5.17	< 0.001
GABRD	NM_001046162.1	-3.61	0.035
GAD1	NM_001046252.2	-3.09	0.003
GANC	XM_010814979.1	-4.07	0.008

GAS8	XR_806569.2	2.18	0.033
GAT	XM_010817529.2	2.96	0.021
GBP2	NM_001038050.2	-3.96	0.006
GDF11	XR_139401.4	-6.29	0.006
GPATCH2L	NM_001046141.3	2.28	0.041
GPKOW	XM_015470800.1	-3.10	0.048
GPR146	NM_001033610.1	-6.36	0.005
GPR157	XM_002686581.4	-3.56	0.046
GPR61	XM_015460321.1	-3.59	0.025
GPR84	XM_005214758.3	2.62	0.027
GRIN3A	XR_237996.2	-3.47	0.050
GSTT2	XM_005205614.3	-7.35	0.013
GTF3C2	NM_001075185.1	2.81	0.005
HEATR4	XR_234113.3	-5.19	0.014
HNRNPM	XM_015472791.1	2.59	0.033
HOMEZ	XR_001501190.1	2.39	0.017
HOXA2	XM_015459284.1	5.48	0.031
HOXA3	NM_174366.1	-4.04	0.049
HOXB1	XR_001494726.1	2.20	0.006
HPS1	NM_001046098.2	-5.35	0.025
HPS1	XM_015474381.1	-5.29	0.021
Hsp40	NM_001191158.3	-5.45	< 0.001
Hsp40	XM_005220403.2	-3.74	0.009
HUWE1	NM_001206164.1	4.37	0.004
IFT122	XM_015458461.1	3.55	0.015
IGFN1	NM_001034378.2	-7.18	0.026
IGIP	XM_005221765.3	4.59	0.043
IL13RA1	XR_808941.1	3.62	0.004
KANSL3	NM_001078119.1	8.35	0.029
KANSL3	XM_005212357.3	9.32	0.022
KCNA7	XM_015471222.1	-5.22	0.027
KCNE2	NM_001034502.1	-7.58	0.022
KCNJ10	NM_174267.2	-2.14	0.038
KCNJ14	XM_010819766.2	-4.20	0.042
KCTD10	XM_015471466.1	-5.32	0.015
KIAA0825	NM_001045875.1	-2.40	< 0.001
KIAA1210	NM_001046222.2	-2.07	0.049
KIAA1324	NM_001102204.1	-3.64	< 0.001
KIDINS220	XM_015458460.1	3.35	0.045
KIF5C	XM_005216768.3	-3.20	0.015
KLF12	XM_010815133.1	-2.83	0.024

KLF7	NM_001046011.2	-6.07	0.001
KLHL38	XM_015473263.1	4.14	0.041
KLKB1	NM_001034538.2	-10.90	< 0.001
KMT2E	NM_001206638.1	5.34	0.037
LACE1	NM_001293133.1	-4.80	0.014
LAYN	NM_001001159.1	-2.66	0.023
LCN2	XM_005205749.1	-2.91	0.047
LECT1	XM_002693773.4	6.33	0.002
LEF1	XR_810634.1	2.50	0.040
LMOD3	NM_001078079.1	-2.48	0.015
LOC100196901	XM_005210211.3	4.55	0.033
LOC100298530	XR_811550.2	4.03	0.020
LOC100301024	XR_233523.3	2.39	0.045
LOC100847236	XM_010802178.2	5.05	0.039
LOC100847951	NM_001206865.1	-4.05	0.022
LOC100848598	XM_010802317.1	-7.21	< 0.001
LOC101902372	NM_181014.2	-3.20	0.003
LOC101902884	XR_233810.3	4.59	0.040
LOC101903138	XM_015475013.1	-6.33	0.021
LOC101903165	XM_005214191.3	-3.09	0.041
LOC101903356	XM_015461798.1	-7.12	0.042
LOC101903540	XR_806210.1	-5.23	0.013
LOC101903694	XM_005227228.3	-4.01	0.031
LOC101903773	NM_001046420.1	-3.15	0.010
LOC101903838	XR_083578.5	-4.75	0.003
LOC101904103	XR_240706.2	-3.01	0.008
LOC101904136	XM_002692205.5	3.34	0.035
LOC101904492	NM_001078142.1	-3.15	0.032
LOC101904723	XM_003586580.4	6.25	0.006
LOC101905074	NM_001077847.1	-2.72	0.010
LOC101905455	NM_001078144.1	-4.37	0.007
LOC101906410	XM_010815399.2	2.97	0.047
LOC101906731	NM_001007813.2	-11.08	0.018
LOC101907132	NM_174128.3	6.80	0.009
LOC101907397	XR_237845.2	-2.95	0.015
LOC101907566	XM_005211286.2	2.07	0.007
LOC101907577	XM_010818297.2	7.38	0.049
LOC101907959	XM_005212845.3	3.29	0.011
LOC101908758	XR_001501779.1	2.57	0.043
LOC104968527	XR_001502166.1	6.17	0.006
LOC104968935	NM_001100345.2	-2.43	0.041

LOC104969062	NM_001206327.1	-4.50	0.001
LOC104969064	XR_001501940.1	2.28	0.032
LOC104969451	XM_005208871.2	4.75	0.053
LOC104969535	XM_003587273.4	-3.00	0.039
LOC104969884	XM_015458981.1	3.33	0.016
LOC104970554	XM_015458338.1	3.53	0.024
LOC104970598	NM_001035490.2	-5.07	0.004
LOC104970629	XM_002687246.2	-5.88	< 0.001
LOC104971207	XM_015458467.1	-4.41	0.009
LOC104971255	XM_005216725.3	4.55	0.031
LOC104971296	NM_174137.2	-3.34	0.003
LOC104971307	NM_174520.2	-5.80	0.047
LOC104971616	XR_001502344.1	4.67	0.016
LOC104971676	XM_010815027.1	-4.16	0.036
LOC104971982	XM_005208557.3	6.96	0.036
LOC104972131	XR_816273.2	3.41	0.010
LOC104972327	NM_001075827.1	-2.30	0.036
LOC104972392	XM_005218906.3	-2.88	0.021
LOC104972592	XM_005213547.2	-2.86	0.043
LOC104972706	XM_005205944.2	-5.04	0.029
LOC104973057	NM_001034377.1	-4.57	0.002
LOC104973118	XM_002691898.4	-7.95	0.015
LOC104973701	XM_005228588.3	-4.36	0.025
LOC104973873	XR_001494592.1	7.43	0.021
LOC104974006	XR_233293.3	-3.26	0.053
LOC104974259	NM_001029846.1	-4.00	0.035
LOC104974282	NM_001075924.1	-10.25	< 0.001
LOC104974691	XM_015474190.1	3.63	0.044
LOC104974705	XM_005201446.3	4.02	0.031
LOC104974752	XR_001500663.1	3.60	0.026
LOC104974953	XM_015459121.1	6.65	0.046
LOC104975124	XM_010810651.1	-2.36	0.005
LOC104975402	NM_001080309.2	-4.77	0.009
LOC104975559	NM_174538.2	-8.27	0.021
LOC104975982	XR_001495590.1	5.93	0.050
LOC104976302	XR_812022.1	-3.08	0.018
LOC104976319	XM_010814042.2	3.40	0.043
LOC104976379	XM_005213754.3	4.87	0.028
LOC107131247	NM_001034646.1	-4.32	0.026
LOC107131367	XR_001502255.1	5.28	0.053
LOC107131455	XM_005223982.3	-5.99	< 0.001

LOC107131534	XM_010806124.2	3.79	0.020
LOC107131539	XM_005201551.3	2.82	0.018
LOC107131569	NM_001206057.1	4.17	0.015
LOC107131637	XR_001500877.1	3.20	0.029
LOC107131652	NM_205779.1	-2.75	0.002
LOC107131806	XM_010813555.2	-5.32	0.036
LOC107132333	NM_001046464.1	-4.50	0.003
LOC107132398	NM_001192165.1	2.12	0.035
LOC107132398	NM_001102539.1	-3.39	< 0.001
LOC107132458	XM_015472633.1	-3.20	0.033
LOC107132475	XR_001495153.1	-8.08	0.007
LOC107132635	XR_808332.2	-4.05	0.003
LOC107132666	NM_001038065.1	-2.43	0.018
LOC107132851	XM_005211495.3	3.81	0.001
LOC107132948	NM_001034220.2	-4.19	0.050
LOC107132958	XM_005216400.2	-5.18	< 0.001
LOC107133105	XM_005225939.2	-5.21	< 0.001
LOC107133298	NM_001035079.2	-4.63	0.002
LOC107133343	XM_002691498.4	2.30	0.010
LOC505383	XM_005222242.3	3.28	0.012
LOC510990	NM_001098946.1	-2.77	0.036
LOC511498	NM_001105637.2	-5.44	0.027
LOC515828	XM_002688782.5	-2.17	0.046
LOC518161	XR_235491.3	7.59	0.009
LOC519654	XR_806217.1	4.99	0.026
LOC519807	NM_001075763.2	4.55	0.017
LOC524642	NM_001114080.1	-2.66	0.035
LOC526041	XR_816185.2	2.69	0.030
LOC528282	XR_816639.1	2.62	0.005
LOC528802	XR_807960.2	5.69	0.001
LOC528802	NM_173900.2	-2.34	0.004
LOC531038	NM_174553.2	-2.92	0.028
LOC532036	XR_001494913.1	-6.13	0.011
LOC532207	XM_015473937.1	-3.35	0.005
LOC615145	XM_010802238.2	-5.40	0.030
LOC615451	NM_001017940.1	-3.68	0.002
LOC616662	XR_814838.2	-6.54	0.043
LOC616883	NM_001192048.1	5.63	0.016
LOC616977	XM_015473845.1	4.38	0.027
LOC781001	NM_001101095.1	6.12	0.054
LOC781112	NM_001101946.1	-2.10	0.042

LOC782601	XR_235741.3	-2.27	0.024
LOC784148	XR_808741.2	4.33	0.016
LOC784741	XM_005213773.3	4.19	0.029
LOC784866	XM_002684775.4	2.33	0.043
LRCH3	NM_174618.2	-4.04	0.045
LRCH3	NM_001205793.1	-2.19	0.042
LRRC27	NM_001206396.1	-5.52	0.018
LYRM9	NM_001034259.1	-2.38	0.035
MAGI3	XM_010806029.2	-5.92	0.002
MALRD1	NM_174006.2	-12.25	0.011
MARCH8	XM_002696386.4	-4.09	0.009
MAVS	NM_001205630.1	-3.30	0.004
MEAF6	XR_811097.2	3.94	0.025
MEIOC	XR_001494301.1	2.69	0.039
MET	XR_001494932.1	4.69	0.003
METTL6	XM_015473572.1	4.98	0.050
MFAP4	XM_005202201.3	3.14	0.008
MGC134577	XR_001495621.1	-4.19	0.043
MIER1	NM_173883.2	3.06	0.014
MREG	XM_015472372.1	-3.86	0.050
MSLN	XR_805513.2	5.84	0.018
MTIF3	NM_175788.1	-3.26	0.023
MTMR3	NM_173940.2	-3.79	0.033
MTMR8	XM_015459817.1	2.97	0.018
MTMR9	XM_003585820.4	-3.61	0.028
MYEF2	XM_015472259.1	-3.00	0.015
NBEAL1	XM_010803262.2	3.48	0.040
NCOA6	XM_010821633.2	-4.60	0.012
NEDD9	XM_015473228.1	3.71	0.014
NEK2	XM_015460065.1	2.88	0.031
NEK4	NM_001013599.1	-2.87	0.011
NEURL4	XM_002687066.3	-6.83	0.002
NHS	XM_010814080.2	2.48	0.006
NID1	NM_001205786.1	-3.58	< 0.001
NKX1-2	XM_002692586.5	3.91	0.052
NLGN1	NM_001205815.1	3.56	0.019
NR3C2	XR_812760.2	-6.73	0.015
NR6A1	XR_238777.3	4.58	0.003
NRG1	XM_005205367.3	6.21	0.023
ODC1	XM_002685010.5	2.66	0.047
OPLAH	XM_005217352.3	4.15	0.004

OR51Q1	XM_002692548.4	-2.77	0.003
ORMDL3	XR_001497049.1	-5.23	0.039
OSGIN2	NM_001206588.2	-2.53	0.048
OXSR1	XM_005213671.3	-5.83	0.049
PAFAH2	NM_001024537.2	-3.47	0.035
PAICS	NM_001045941.1	-5.61	0.027
PAN3	XM_002685877.3	-3.62	0.024
PAN3	XR_001494959.1	2.84	0.024
PAPD5	XR_001494566.1	-4.88	0.028
PAPOLA	XM_015460281.1	2.08	0.049
PCMTD1	NM_001075854.1	6.27	0.014
PCNT	XR_816640.1	2.37	0.049
PCNT	NM_001101155.1	3.47	0.048
PDP1	XM_015459297.1	-5.53	0.014
PITX2	NM_001046149.1	-3.54	0.050
PKD2L2	XR_810842.2	7.75	0.050
PKLR	NM_001076460.1	-7.86	0.015
PLA2G4E	NM_001314031.1	-5.48	0.024
PLCB1	XR_001501293.1	-5.08	0.013
PLCH1	XM_010815628.2	3.63	0.044
POLD3	NM_001078134.2	-3.97	0.020
POLR2K	NM_001034698.2	-3.23	0.053
PPAPDC1A	XM_005219261.3	4.29	0.035
PPP2R1B	NM_001303535.1	10.17	< 0.001
PREX2	NM_001077941.1	-4.20	0.002
PROCA1	XR_807629.2	4.65	0.038
PRPF40B	NM_001024525.1	-3.15	0.047
PRRT1	NM_173985.2	-3.88	0.054
PUS3	NM_001105347.1	-2.01	0.051
RBM26	XM_015470778.1	-3.14	0.003
rC	XM_002688518.4	-2.64	0.052
RCC1	NM_001034039.2	-6.73	0.006
RCE1	NM_001034340.2	-3.38	< 0.001
RFTN2	NM_001034788.2	-3.73	0.025
RFWD3	NM_001078054.2	-5.75	0.037
RGL3	NM_001045902.1	-2.92	0.003
RGS20	XM_002689821.5	-4.73	0.006
RGS21	NM_001034223.2	-3.74	0.047
RHOBTB1	XR_237125.1	-4.91	0.014
RNF38	NM_001206734.1	-3.74	0.016
S100A12	NM_001046095.2	-3.24	0.004

SAMD14	NM_173945.4	-9.52	0.041
SCAF11	XR_233726.3	-5.46	0.007
SCMH1	XM_005212803.3	2.18	0.021
SEC23B	XM_005207719.3	-6.00	0.038
SECISBP2L	NM_001206550.1	5.40	0.042
SEMA3G	XM_015473379.1	-4.23	0.027
serotonin	XM_005212593.3	-2.45	0.017
SHANK1	NM_001100362.1	-4.59	0.004
SIM2	NM_174716.1	-2.54	0.009
SLC25A19	XM_005216887.3	2.93	0.044
SLC25A51	XM_005206042.2	-8.84	0.005
SLC26A3	XR_001501105.1	2.29	0.034
SLC35G6	XM_010818583.2	5.15	0.052
SLC39A10	XR_001494912.1	-2.03	0.053
SLC39A6	NM_001078008.1	2.81	0.034
SLC4A2	XR_810336.2	2.93	0.036
SLC7A6	XM_002689999.3	8.00	0.012
SLMAP	NM_001046043.2	-5.41	0.010
SMC1B	XM_005209429.3	-9.07	< 0.001
SMLR1	NM_001103258.1	8.47	0.037
SMLR1	XM_005210928.1	10.61	0.013
SMPD5	NM_001076841.1	-4.56	0.012
SNAPC5	XM_005220964.3	4.79	0.029
SNAPC5	NM_205774.3	-5.90	0.010
SNRPB2	XM_015472222.1	-5.07	0.054
SNX16	NM_001075192.1	2.67	0.028
SNX24	XR_804947.2	3.05	0.032
SPATA7	NM_001097568.2	-3.49	0.004
ST7L	NM_001113284.1	-6.44	< 0.001
ST8SIA2	XM_010822151.2	2.05	0.049
STARD10	NM_001046157.1	-8.55	< 0.001
STAU1	XR_811947.1	-3.25	0.035
STRA6	XM_010819302.2	-5.75	0.003
STX17	NM_001111103.2	-2.24	0.024
TAAR1	XM_010819479.2	-4.75	< 0.001
TDRD1	XM_015471990.1	-2.48	0.021
TET2	NM_001100374.1	6.65	0.054
TET3	NM_001099055.1	-4.19	0.022
THEMIS2	NM_001205594.1	-2.45	0.021
TLE2	NM_001192919.1	3.54	0.047
TLR2	NM_001075656.1	-2.68	0.001

TLR3	XM_010817188.2	3.82	0.015
TMEM104	XM_005204062.3	3.15	0.012
TMEM106A	NM_001076095.1	4.80	0.002
TNFSF11	XM_005201107.3	-3.04	0.017
TOM1L2	XM_003585891.4	-5.69	< 0.001
TPD52L1	NM_001205930.1	4.70	0.021
TPRG1	NM_001192212.1	3.11	0.048
TRIM68	XM_005218978.3	-4.00	0.025
TRMT5	XM_015460145.1	-4.03	0.022
TRMU	NM_001192732.1	4.33	< 0.001
TSC1	NM_001076148.2	-2.51	0.039
TSPYL6	XM_005207869.3	4.08	0.031
TTC24	NM_001114857.2	-2.28	0.004
TTC6	NM_001105327.2	-5.38	0.009
TTPAL	XM_005227486.3	2.84	0.028
TXNDC16	NM_001075201.1	-5.00	0.016
TYK2	XM_010813725.2	5.73	0.046
UTRN	XR_001496942.1	2.93	0.039
VCPKMT	XM_015463251.1	-3.40	0.017
VPS16	XM_005219180.3	-5.49	0.002
VRK2	XM_010812331.2	3.81	0.014
VSIG8	NM_174796.1	-6.40	0.017
VTI1A	NM_001075698.1	-2.26	0.007
WDFY3	NM_001001163.1	-8.62	0.039
WDR54	NM_001192079.1	-6.38	0.041
WDR78	XR_001494810.1	5.01	0.003
WNT16	NM_001012999.2	3.60	0.008
XPNPEP1	XM_010801600.2	5.35	0.046
XRCC3	NM_001075821.2	2.15	0.054
YEATS2	XR_001495525.1	-3.18	0.034
YIPF7	XM_015475834.1	8.09	0.032
ZBTB49	NM_173934.1	-7.46	0.005
ZC3H12C	NM_001046118.2	-4.87	< 0.001
ZCCHC6	NM_173951.2	2.15	0.052
ZDHHC3	NM_001081519.1	-3.83	< 0.001
ZFR	NM_001081536.2	-8.24	0.013
ZNF205	NM_001192398.1	2.25	0.050
ZNF268	NM_001002892.2	-2.44	0.052
ZNF37A	XM_010817157.2	-4.43	0.054
ZNF501	XM_010807305.2	7.85	0.036
ZNF554	NM_001102132.2	5.40	0.012

ZNF569	XR_805807.2	4.09	< 0.001
ZNF729	XM_015474150.1	11.11	< 0.001
ZNF793	NM_001032294.1	-3.56	0.034
ZSCAN26	XM_005203838.3	-4.39	0.047

Genes are differentially expressed ($\log_2$ fold change ≥ 2 or ≤ -2 and a $P \leq 0.05$) in oocytes collected from bacteria infused heifers compared to heifers infused with vehicle medium at day 4 relative to infusion.

Supplemental Table 5. Differentially expressed genes in oocytes of bacteria infused heifers at day 60.

**Gene symbol**	**Accession number**	**Log$_2$ fold change**	***P*-value**
ABCB6	NM_001098156.2	2.06	0.021
ABHD14B	NM_001105441.1	3.41	0.020
ABRA	NM_001192234.1	-3.25	0.009
ACADM	XM_010803370.2	2.22	0.047
ACTR5	XM_015474204.1	-2.15	0.007
ADAM22	NM_001191131.1	-8.20	< 0.001
ADAMTS9	XM_005222686.3	2.55	0.007
ADP-ribose	XM_002692548.4	5.71	0.001
AGBL4	XM_015466644.1	2.04	0.018
AIFM2	XM_005226487.3	3.12	0.019
AKAP4	NM_174235.1	2.11	0.028
ALG5	XR_236608.3	-2.82	0.040
ANAPC5	XM_005217905.3	2.82	0.031
ANKRD33B	XM_010816845.2	3.36	0.031
AOX2	NM_001281918.1	4.11	0.013
APOE	NM_173991.2	2.87	0.018
APOOL	XM_015461629.1	4.57	0.031
ARHGAP6	NM_001191354.1	3.05	< 0.001
ARL10	NM_001076320.2	2.07	0.045
ARMCX4	XM_003588124.4	3.68	0.018
ASB3	NM_001076927.1	-2.45	0.001
ASB9	NM_001191166.1	4.80	0.047
ASPH	XM_015474457.1	2.68	0.003
AXL	XM_010814979.1	4.25	0.034
BAHCC1	XM_015458949.1	4.69	0.008
BCAM	NM_174741.2	2.17	0.004
BCDIN3D	NM_001075216.1	4.84	< 0.001
BCL11A	NM_001076121.1	2.32	0.018
BEX2	XM_005227757.3	2.14	0.013
BTC	NM_173896.2	2.84	0.010
C17H22orf31	NM_001101245.2	3.75	0.009
C1H3orf17	XM_015471313.1	3.80	0.040
C1H3orf58	NM_001205651.1	2.20	0.018
C1S	NM_001076550.1	6.83	0.046
C21H14orf132	NM_001252510.1	2.65	0.016
C24H18orf63	XM_015460035.1	2.21	< 0.001
C5H12orf73	XM_005206482.1	4.53	0.051
C6H4orf26	XM_002688369.4	-4.33	0.005

C7H19orf45	XM_015472034.1	2.44	0.022
CA10	NM_001079609.1	3.55	< 0.001
CABP1	NM_174254.2	6.69	0.006
CACNA2D3	XM_015459579.1	3.83	0.032
CADM4	XM_010815133.1	2.64	0.026
CAGE1	XM_015460014.1	3.32	0.040
CALCB	NM_001143737.2	-2.06	0.021
CALHM4	NM_001101133.1	-6.09	0.024
CCDC80	NM_001098982.2	2.44	0.005
CCNC	XM_005210847.3	3.05	0.004
CCND2	NM_001076372.1	2.06	< 0.001
CCNT2	XM_010802097.2	3.88	0.018
CCT6A	NM_001034542.2	2.08	< 0.001
CD151	NM_001035347.1	2.80	0.033
CD58	XM_010803137.1	3.18	< 0.001
CD99	NM_001244214.1	2.53	0.005
CDAN1	XM_005211641.3	4.71	0.050
CDH13	NM_001035277.2	-2.14	0.026
CDR2L	XM_010816569.2	2.29	0.032
CELF1	XM_010812844.2	4.93	< 0.001
CFAP47	XM_015461736.1	-3.20	0.042
CHD9	XM_005218694.3	3.05	0.022
CHML	XM_005216871.3	2.26	0.010
CHRNA10	XM_005216210.2	2.66	0.013
CHRNA9	NM_001192546.1	3.02	0.010
CIAO1	NM_001037482.2	2.40	0.006
CLK1	NM_001102271.1	2.15	0.009
CLU	NM_173902.2	2.45	0.048
COL18A1	NM_001083388.2	4.27	0.009
COL1A1	NM_001034039.2	4.20	< 0.001
COL3A1	NM_001076831.1	7.19	0.032
COL4A2	XM_015473936.1	2.66	0.030
COL5A1	XM_015473738.1	7.00	< 0.001
COL6A2	NM_001075126.1	2.58	0.001
COL6A2	XM_005202053.3	2.11	0.002
CPNE4	XM_003585702.3	-6.02	0.014
CSRP3	NM_001024689.3	10.07	0.008
CSTB	NM_001100362.1	5.92	0.053
CTHRC1	NM_001081536.2	7.93	< 0.001
CUX1	XM_010819623.1	3.45	< 0.001
CYP19A1	NM_174305.1	4.52	< 0.001

CYP27A1	NM_001083413.2	2.27	0.003
DACH1	XM_010810843.2	3.19	0.012
DCHS1	XM_002693149.4	4.69	0.033
DDAH2	NM_001034704.1	2.70	0.048
DGKH	XM_010810743.2	-3.88	0.019
DHRS7	NM_001046162.1	3.51	0.009
DHX9	NM_174036.2	2.08	< 0.001
DNER	XM_003585774.2	-5.97	0.006
EGR1	NM_001045875.1	2.85	0.013
EHHADH	NM_001075780.2	2.09	0.053
EID2	NM_001078049.1	2.29	0.011
EMB	XM_015459072.1	2.43	0.032
EMB	XM_005221538.3	2.90	0.053
EMX2	NM_001075845.1	2.26	0.054
ENPP3	NM_001075923.2	3.17	0.013
EPHX2	NM_001075534.1	2.70	0.011
ERBB3	NM_001103105.1	3.29	0.022
ESCO1	XM_005224099.3	3.72	0.038
ETF1	XM_005209423.3	2.68	< 0.001
F11	NM_001008665.1	5.66	0.002
FADS2	NM_001083444.1	2.26	0.044
FAM166B	NM_001038513.2	2.82	0.043
FAM20C	XM_002698175.5	2.21	0.030
FBN2	NM_001278588.1	3.17	0.002
FBN3	XM_015472041.1	2.23	0.050
FGF11	XM_005220239.3	2.03	0.012
FGF12	XM_005201556.3	2.86	0.009
FIGN	XM_003585744.4	4.99	0.024
FKTN	XM_002689900.5	2.19	0.015
FOLH1B	NM_001101858.2	2.50	0.014
FOS	NM_182786.2	4.02	0.010
FOXL2	NM_001031750.1	2.53	0.002
FOXP1	NM_001083689.1	3.16	0.041
FRAS1	XM_003586218.3	-4.96	0.040
FRK	XM_002690084.5	2.49	0.004
FUCA2	NM_001205818.1	2.26	0.002
FUT10	NM_182987.1	2.38	0.031
FUT11	XM_002698914.4	2.17	0.003
FXYD3	NM_001079646.1	7.26	< 0.001
FZD10	NM_001098033.1	3.21	< 0.001
G0S2	XM_005217335.3	3.76	0.034

GABRD	NM_001081715.1	-2.41	0.050
GALNS	NM_001206329.1	2.13	< 0.001
GATA6	XM_002697727.2	2.95	< 0.001
GBA	XR_805328.2	3.30	0.051
GLMP	NM_001105476.1	4.07	0.028
GLYCTK	NM_001045987.2	3.38	< 0.001
GPALPP1	XM_015473775.1	4.81	0.042
GPC1	XM_010803936.1	2.43	0.018
GPIHBP1	XM_005215283.3	4.59	0.048
GPLD1	XM_015459945.1	-2.10	0.028
GPM6B	XM_005228439.3	3.76	0.041
GPR50	XM_002699646.3	11.00	0.006
GPRC5B	XM_010819302.2	3.87	0.009
GPT	XM_015474359.1	3.82	0.031
GTPBP1	XM_010805710.2	2.76	0.045
H19	NR_003958.2	8.30	0.030
H2AFY2	NM_001076086.1	2.69	0.032
H2AFZ	XM_010805947.2	2.50	< 0.001
H2AFZ	NM_174809.2	2.97	0.001
H3F3C	NM_001099370.2	-2.05	0.052
HERC6	XM_010806029.2	5.75	0.050
HEXDC	NM_001101864.2	2.02	0.012
HHIPL1	XM_002696835.2	2.06	< 0.001
HIST1H1B	XM_010818459.2	3.58	0.009
HIST1H2AC	NM_001205596.1	2.30	0.039
HIST1H2BM	XM_010818508.2	-3.58	0.051
HIVEP2	XM_005211000.3	3.86	0.001
HMGN5	XM_002699936.4	2.69	0.004
HMHA1	NM_001205741.1	2.19	0.044
HOPX	XM_005207941.3	2.76	0.016
HOXC10	XM_003586048.3	2.53	0.011
HOXC8	XM_002687232.3	3.62	0.037
HPSE	NM_174082.2	2.31	< 0.001
HSD17B7	NM_001083375.1	2.63	0.020
HSD3B1	NM_174343.3	2.30	0.005
Hsp40	NM_174096.2	5.60	0.010
IDH3B	NR_030768.1	5.08	0.004
IFI27	NM_001038050.2	5.28	0.024
IFI44	XM_005204427.3	6.95	0.050
IFI6	XM_010802541.2	4.22	0.054
IFI6	NM_001075588.1	4.25	0.042

IFI6	XM_005203121.3	4.26	0.040
IFIT5	NM_001075698.1	3.17	0.046
IFITM3	NM_181867.1	3.35	0.007
IFITM3	NM_001078141.2	3.88	0.012
IFT74	XM_015472416.1	5.90	0.031
IKBIP	NM_001081736.2	2.04	< 0.001
IL27RA	NM_001098028.1	2.34	< 0.001
IL2RG	NM_174359.1	-5.62	0.004
IL33	NM_001075297.1	4.08	0.014
IMPG1	NM_174362.2	3.02	0.012
inactive	XR_001495594.1	2.62	0.001
INSL3	NM_174365.2	2.85	< 0.001
IPO8	NM_001206120.1	2.47	< 0.001
IRF9	NM_001024506.1	2.96	0.030
ISG15	NM_174366.1	6.99	0.026
ISG20L2	NM_001046217.2	2.29	< 0.001
ITGA2	NM_001166499.1	2.18	0.002
JAM2	NM_001083736.1	2.97	< 0.001
JSP.1	NM_001040498.1	2.60	0.004
KCNIP4	XM_005207804.3	3.25	0.055
KCNMB4	NM_001192523.1	5.42	< 0.001
KCTD7	NM_001102545.1	2.62	0.016
KCTD9	NM_001102269.1	-2.50	0.028
KIAA0907	XM_015462254.1	5.36	0.017
KIF21A	NM_001206918.1	2.64	0.004
KLF10	NM_001168462.1	2.74	< 0.001
KLHL13	XM_005227425.3	3.01	0.026
KLHL28	NM_001099030.1	2.20	0.004
KLHL31	NM_001101955.1	2.27	0.002
KLK4	NM_001205621.1	2.09	0.028
KRCC1	XM_005212758.3	4.35	0.037
KRT8	NM_001033610.1	3.97	0.028
LACE1	XM_010808444.2	2.59	0.051
LAPTM4B	NM_205802.1	2.19	< 0.001
LAPTM4B	XM_010812166.1	4.95	0.011
LDLR	NM_001166530.1	3.00	0.009
LEXM	XM_015465619.1	2.78	0.018
LGALS3BP	XM_005221235.2	2.22	0.013
LGALS9	NM_001015570.3	2.71	0.007
LGALS9	NM_001039177.2	4.05	0.013
LHFPL2	XM_015472989.1	2.76	0.017

LIG1	XM_010814981.2	4.71	0.022
LOC100296257	XM_015472372.1	-2.03	0.037
LOC100298356	XM_015471926.1	6.79	0.026
LOC100298530	XM_010807766.2	4.26	0.045
LOC100337132	XM_002688038.5	3.96	0.025
LOC100847632	XR_236936.3	2.71	0.008
LOC100847917	XR_139649.4	3.06	0.021
LOC100848315	XR_812022.1	2.94	0.037
LOC100848375	XR_139401.4	8.40	0.002
LOC100848469	XR_139201.4	2.61	0.003
LOC100848581	XR_139381.3	2.08	0.037
LOC100848930	XR_811094.2	2.22	0.034
LOC100848933	XR_813725.2	5.82	0.033
LOC100849652	XR_805435.2	2.12	0.021
LOC101902048	XR_001497410.1	3.26	0.005
LOC101902265	XM_015471022.1	2.91	0.023
LOC101902276	XR_814785.2	3.37	0.030
LOC101902325	XR_809292.2	-2.87	0.035
LOC101902439	XR_001502295.1	2.40	0.020
LOC101902757	XR_237544.2	5.36	0.048
LOC101903564	XR_238481.3	7.98	< 0.001
LOC101903868	XR_234904.3	7.06	0.043
LOC101904130	XR_235431.3	7.14	0.030
LOC101904209	XR_236298.3	6.62	0.049
LOC101904673	XR_001494995.1	3.73	0.003
LOC101905239	XR_001500848.1	-2.53	0.017
LOC101905312	XR_240040.1	2.15	0.017
LOC101905390	XR_001495269.1	2.35	0.049
LOC101905505	XM_005227934.3	5.56	< 0.001
LOC101906072	XR_238597.3	2.94	0.026
LOC101906559	XR_001495015.1	2.55	0.030
LOC101906866	XR_237566.3	-3.35	0.039
LOC101906931	XR_236788.3	2.36	0.008
LOC101907330	XR_001502011.1	4.60	< 0.001
LOC101907782	XR_233800.3	3.81	< 0.001
LOC101907813	XR_238604.2	-5.80	0.031
LOC101908104	XR_233941.3	4.89	0.005
LOC104968894	XM_010806144.2	-2.62	0.038
LOC104969373	XR_001500839.1	-3.82	0.004
LOC104969373	XR_001500838.1	-3.39	0.029
LOC104969425	XR_001500893.1	3.81	0.036

LOC104969678	XM_015472972.1	7.88	0.026
LOC104970219	XM_015473876.1	2.25	0.014
LOC104970563	XR_817049.1	2.60	0.029
LOC104970887	XR_816158.2	-2.19	0.041
LOC104971095	XM_015461070.1	3.21	0.044
LOC104971266	XR_804944.2	3.60	0.034
LOC104972131	XR_806217.1	2.17	0.023
LOC104972256	XR_806381.2	4.06	0.043
LOC104972288	XM_010804675.2	2.11	0.031
LOC104972545	XR_806892.2	9.21	0.014
LOC104972595	XR_807025.2	3.71	0.039
LOC104973209	XR_809433.2	4.88	0.017
LOC104973542	XR_810121.1	-2.40	0.017
LOC104974645	XR_812369.2	-3.54	0.005
LOC104974785	XR_001502326.1	-3.16	0.012
LOC104975755	XM_015460078.1	2.60	0.010
LOC104975788	XR_001501437.1	7.68	< 0.001
LOC104975972	XR_815704.1	5.02	0.021
LOC104976976	XR_001500422.1	-3.59	0.047
LOC107131209	XM_005223693.3	2.96	0.014
LOC107131217	XM_005220063.3	4.93	0.040
LOC107131573	XR_001494474.1	3.46	0.052
LOC107131641	XM_015459398.1	2.96	0.021
LOC107132049	XR_001495589.1	-3.43	0.048
LOC107132061	XR_001495601.1	5.20	0.017
LOC107132236	XR_001501943.1	4.41	< 0.001
LOC107132398	XM_015469045.1	2.42	0.025
LOC107132574	XR_001500518.1	-2.74	0.014
LOC107132614	XR_001500599.1	4.03	0.008
LOC107132641	XR_001500678.1	3.10	0.016
LOC107132642	XR_001500679.1	-5.01	0.021
LOC107132678	XR_001500766.1	-4.50	0.015
LOC107132708	XM_015471179.1	6.46	0.036
LOC107132851	XR_001501173.1	2.14	0.020
LOC107133063	XR_001501779.1	2.18	0.016
LOC107133103	XR_001501814.1	2.90	0.043
LOC107133123	XR_001501854.1	2.07	0.046
LOC107133179	XR_001501952.1	4.75	0.014
LOC107133214	XR_001501996.1	3.52	0.005
LOC107133310	XR_001502245.1	5.34	0.038
LOC504219	XM_010804682.2	4.50	0.054

LOC505918	XM_002688782.5	3.26	0.012
LOC512684	XM_015458443.1	3.54	0.007
LOC513508	XM_015475522.1	2.51	< 0.001
LOC515000	XM_002696020.3	3.44	0.041
LOC515149	XM_010816984.2	2.68	0.017
LOC518161	XM_003585994.3	3.73	0.003
LOC521676	XM_002693413.1	3.50	0.029
LOC527385	XM_015458372.1	7.33	< 0.001
LOC527397	XM_015465165.1	4.69	0.018
LOC530348	XM_002696335.5	8.83	0.002
LOC531038	XR_816944.2	4.73	0.035
LOC616942	NM_001206459.1	5.00	< 0.001
LOC616948	XM_005216143.3	4.77	0.045
LOC618737	XM_002688577.5	5.11	0.030
LOC782776	XR_083578.5	-2.96	0.045
LOC784007	XR_805310.2	2.06	0.052
LOC784007	XM_010802841.1	4.74	0.025
LOC784148	XM_015471974.1	5.61	< 0.001
LOC788150	XM_015458332.1	-2.15	0.050
LOC789374	XR_001494249.1	2.17	0.028
LOC789384	XM_015474370.1	4.99	0.017
LPL	NM_001075120.1	3.51	< 0.001
LRRC75A	XM_002695816.4	2.46	0.018
LTA4H	NM_001034280.1	2.12	0.021
LY6G5B	NM_001192202.1	2.36	0.004
MAGED2	XM_005228224.3	2.08	0.055
MAGED2	XM_005228225.2	2.49	0.048
MAGED2	NM_001075665.1	5.50	0.041
MAML2	NM_001098050.1	5.42	0.012
MAN1A2	NM_001206877.1	3.59	0.028
MAOA	NM_181014.2	3.50	< 0.001
MAP1LC3C	XM_010813121.2	7.11	0.044
MARCH1	XM_015471481.1	-4.43	0.036
MARVELD1	NM_001101262.1	2.25	0.002
MATN2	NM_001102528.1	3.67	0.006
MBNL3	XM_015461425.1	2.47	0.048
MBTPS2	XM_010822145.2	3.24	< 0.001
MCC	XM_010808842.2	3.09	0.002
MCRS1	XM_005206295.2	2.25	0.042
MECOM	XM_010801437.2	-2.62	0.034
MEI4	XM_015472775.1	2.56	0.040

METTL22	XM_005224414.2	-3.42	< 0.001
MFGE8	XM_015459180.1	2.16	0.002
MFGE8	NM_176610.1	2.71	0.045
MGC126945	XM_010818383.2	3.33	0.012
MKX	XM_005214349.3	2.29	0.009
MLC1	NM_001076952.1	5.38	0.002
MOB3B	XM_015472417.1	3.09	< 0.001
MPP5	NM_001205951.1	3.02	0.011
MPV17	XR_809851.2	2.38	0.006
MROH8	XM_015474290.1	2.47	0.016
MSANTD2	XM_002699213.4	4.92	0.037
MSANTD3	NM_001076019.1	-2.26	< 0.0012
MSRB3	XM_002687543.3	4.10	0.036
MX1	NM_173940.2	3.32	0.006
MYH3	NM_001101835.1	-2.17	0.009
MYOF	NM_001193212.1	4.35	0.011
MYRFL	XM_010805087.2	-5.07	0.019
N4BP2L1	XM_005213681.3	3.87	0.033
NADSYN1	XM_005227337.3	4.40	0.002
NAE1	XM_015475771.1	3.02	0.004
NAP1L5	NM_001077878.2	4.17	< 0.001
NEFM	XM_005210283.3	6.89	0.035
NICN1	XM_015459614.1	4.16	0.008
NIPSNAP3A	NM_001080298.2	2.24	0.001
NLRP13	XM_015458454.1	-2.65	0.014
NLRP8	XM_010815595.2	-2.07	0.038
NMI	XM_005202429.3	3.31	0.049
NR1D2	XM_015460904.1	2.95	0.024
NR3C1	XM_005209534.3	7.11	0.050
NSMF	XM_010810604.2	2.32	0.008
NT5C3A	XM_005205470.3	4.69	0.053
OAS1Y	NM_001040606.1	5.92	0.037
OC90	XM_002692609.5	-2.36	0.055
OXT	NM_176855.1	2.77	0.049
OXTR	NM_174134.2	3.56	< 0.001
P4HA2	XM_005209037.3	4.72	0.013
PALMD	NM_001035079.2	9.20	0.014
PARP3	XM_010817872.1	2.27	0.002
PCDHGA2	NM_001099368.2	4.27	< 0.001
PCDHGC3	NM_001114080.1	2.28	0.011
PCLAF	NM_001015678.2	2.01	0.004

PCYT2	NM_001035089.1	2.01	0.019
PDK4	NM_001101883.1	4.52	0.020
PELP1	XM_010815963.1	4.38	0.045
PEPD	NM_001080318.2	2.40	0.007
PHF20L1	XM_010811931.2	2.79	0.016
PHGDH	NM_001035017.1	2.10	< 0.001
PIP4P2	NM_001040594.2	3.39	0.003
PLA2G16	XM_005227020.2	2.58	0.047
PLAC8	NM_001076987.2	4.96	0.051
PLEK	NM_001192496.1	2.59	< 0.001
PLOD2	NM_001101149.1	2.37	< 0.001
PLOD2	XM_005201798.3	2.23	0.009
PLPP3	NM_001076473.2	2.13	0.004
PLSCR4	NM_001081732.1	7.20	0.008
PNP	NM_001007818.1	2.50	< 0.001
PON1	NM_001046269.2	-3.49	0.035
POP4	XM_010814562.1	-4.10	< 0.001
POPDC3	XM_005210837.3	3.26	0.026
POSTN	NM_001040479.1	6.33	0.020
PPHLN1	XM_010805060.2	4.49	0.015
PPOX	XM_005203500.3	3.74	0.049
PPP1R3B	NM_001103247.1	3.92	< 0.001
PQBP1	NM_001046389.1	3.88	0.010
PRKA	NM_001193034.2	2.16	< 0.001
PROS1	NM_174438.1	2.49	< 0.001
PRPF4B	NM_001075557.1	3.30	0.027
PSMA2	NM_001034662.2	2.19	< 0.001
PTGES	NM_174443.2	2.30	0.017
PTPRC	NM_001206523.1	4.99	0.032
PTX3	NM_001076259.2	7.10	0.031
PVRL2	XM_005219196.1	2.19	0.001
PXDC1	NM_001076932.1	2.35	0.003
PYGL	XM_005211657.2	2.54	0.001
QPRT	NM_001035446.2	2.79	0.001
QRSL1	NM_001046336.1	2.75	0.032
R3HCC1	XM_002689780.4	2.27	0.006
RAB28	XM_005208311.3	2.47	0.043
RAB38	XM_002699047.5	2.92	0.001
RARRES2	XM_005205802.1	2.33	0.032
RARRES2	NM_001046020.2	2.37	0.020
RASA2	XM_010801546.2	5.33	0.050

RASGRP3	XM_005212534.3	5.60	0.008
RASSF1	NM_001075442.1	2.83	0.025
RBM11	NM_001040567.1	2.57	0.014
RBM45	XM_015474659.1	2.06	0.050
RBM4B	XR_001495455.1	4.24	0.015
RBP1	NM_001025343.1	2.52	< 0.001
RCVRN	NM_174165.2	-5.24	0.003
REST	XM_005207950.3	3.27	0.027
RGS17	NM_001101836.1	2.31	0.002
RGS3	XM_015472689.1	4.03	< 0.001
RGS6	XM_010809581.2	4.26	0.007
RHO	NM_001014890.1	3.02	0.040
RHOQ	NM_001205498.1	3.26	< 0.001
RNASEL	NM_001098165.1	7.99	0.011
RND3	NM_001191158.3	7.27	0.032
RRAGB	XM_005228243.3	2.70	0.012
RSAD2	NM_001045941.1	6.43	0.049
RTN4	NM_001075138.2	2.43	0.002
S100A11	NM_001098856.1	2.30	0.024
SAA3	NM_181016.3	4.03	0.007
SCP2	XM_010803499.2	2.42	0.041
SDC4	XM_002692330.4	3.52	0.040
SECTM1A	XM_005221075.3	3.01	0.038
SEMA4D	NM_001098160.1	4.02	0.002
SERPING1	XM_005216527.1	2.95	0.028
SERPINH1	NM_001046063.1	2.18	< 0.001
SERTAD3	XM_005219036.3	3.38	0.031
SH3BGRL	NM_001035440.2	2.27	0.009
SH3D19	XM_005217479.3	3.00	0.041
SIAE	NM_001045968.2	2.16	0.004
SKIDA1	XM_015474002.1	-3.56	0.041
SLC17A6	XM_010820860.2	3.14	0.028
SLC22A3	XM_002690372.5	3.00	0.031
SLC27A5	NM_001103273.1	-2.10	0.013
SLC34A1	XM_015472154.1	5.47	0.009
SLC38A2	XM_010805036.2	6.48	0.008
SLC38A2	NM_001082424.1	2.29	0.035
SLC4A10	NM_001038128.1	7.28	0.033
SLC4A1AP	XM_005213074.3	3.35	0.046
SLC5A3	XM_005201099.3	3.85	0.051
SLC6A19	XM_015459131.1	7.49	0.031

SMIM3	NM_001244119.1	2.17	0.043
SMIM3	XM_015472307.1	2.98	0.012
SMOC2	NM_001098134.1	2.10	< 0.001
SNAI2	NM_001034538.2	8.51	0.014
SNX12	XM_010821847.2	-2.53	0.039
SOX30	XM_005209673.3	-4.50	0.054
SPARCL1	NM_001034302.2	7.19	0.002
SPOCK2	NM_001101115.1	2.67	0.006
SPSB1	XM_005216941.3	3.05	0.044
SRSF6	XM_005214609.1	2.50	0.004
ST14	NM_001076538.1	-2.51	0.030
ST3GAL5	XM_010810050.2	2.27	0.010
ST8SIA4	NM_001001163.1	7.82	0.019
STK36	NM_001205636.1	2.34	0.025
STK38L	NM_001101092.2	2.74	0.028
STMND1	XM_003587737.4	-2.34	0.020
STRA6	XM_010817072.2	2.22	0.050
STRA6	XM_005221954.3	2.27	0.012
SUSD5	XM_002696906.4	8.43	0.021
SYMPK	NM_001206913.1	-4.25	0.046
SYS1	XR_237001.3	3.14	0.009
TCEAL4	XM_005227856.3	2.68	0.030
TCIM	NM_001035490.2	7.46	0.028
TDG	NM_001083696.2	2.21	< 0.001
TGM2	NM_177507.2	2.69	0.001
THBS2	NM_176872.1	6.31	0.031
THEMIS2	XM_002685688.5	-2.54	0.014
TKTL2	NM_001046025.1	5.63	0.005
TM4SF20	NM_001076981.1	3.76	0.048
TM6SF1	NM_001102295.2	2.23	0.004
TMEM11	XM_005220335.3	-5.03	0.048
TMEM150B	XR_001494233.1	4.24	0.032
TMEM176A	NM_205779.1	3.16	0.008
TMEM178A	NM_001192028.1	2.18	0.046
TMEM246	NM_001102342.2	2.52	< 0.001
TMEM45A	NM_001075405.1	2.23	0.001
TNFAIP6	NM_001007813.2	12.23	0.003
TNFRSF10D	NM_001102327.1	6.22	0.004
TNFRSF1A	NM_174674.2	2.08	0.003
TNFSF18	NM_001077136.1	8.75	0.018
TNS1	NM_174766.2	3.26	0.002

TPBG	XM_002690047.4	2.39	< 0.001
TPRG1	XM_010801257.1	5.84	0.023
TRA2B	NM_001034776.2	2.01	0.012
TRA2B	XM_010801244.2	2.21	0.013
TRANK1	XM_002696940.4	2.91	0.007
TRIM35	XM_002689793.5	2.37	0.008
TSC22D3	NM_001103342.1	2.05	0.002
TSHZ3	XM_015475845.1	3.05	0.016
TTC25	NM_001103308.2	2.93	0.018
TTC27	XM_010809874.2	2.97	0.050
TTC31	XM_015473382.1	5.46	< 0.001
TTC38	XM_010805831.2	2.95	< 0.001
TUBA1A	NM_001166505.1	3.40	0.004
TYW3	NM_001015620.1	3.03	< 0.001
UAP1	XM_005203526.2	2.49	0.047
UBA7	NM_001012284.1	3.57	0.034
UBL7	XR_814045.2	5.66	0.036
UBQLN4	NM_001080295.1	2.08	0.002
UPK1B	NM_174482.2	3.26	< 0.001
UPK1B	XM_005201259.3	3.26	0.013
UPK1B	XM_005201261.3	4.55	0.021
USHBP1	NM_001077137.1	2.67	0.031
USP18	NM_001017940.1	4.99	0.052
VEGFD	NM_001101043.2	2.37	0.024
VNN1	XM_010808580.2	4.08	0.023
VPS13A	XM_010807910.2	3.31	0.037
VSNL1	NM_174490.3	3.41	< 0.001
WBP1	NM_001034346.1	2.15	0.043
WDR31	XM_010808290.2	-3.70	0.025
WDR75	XM_005202201.3	2.02	0.031
XCL1	NM_175716.3	6.28	0.046
ZBBX	XM_015472820.1	5.04	0.026
ZBED9	XM_010801388.2	6.58	0.026
ZBTB38	XM_005201905.3	2.68	0.031
ZBTB47	XM_002707778.3	2.04	0.047
ZCCHC24	XM_003587989.3	4.53	< 0.001
ZDHHC14	NM_001191179.1	2.05	0.011
ZDHHC21	XM_010807799.2	3.98	0.008
ZEB2	NM_001076192.1	3.09	0.005
ZFP37	XM_003586435.4	2.48	0.016
ZKSCAN4	XM_002697448.5	2.46	0.051

ZNF189	XM_005210479.3	2.99	0.027
ZNF274	XM_010815572.1	5.20	< 0.001
ZNF484	NM_001192027.1	3.28	< 0.001
ZNF543	XM_005219811.3	3.06	0.003
ZNF567	XM_010814770.1	5.34	0.013
ZNF619	XM_010817602.2	6.16	0.001
ZNF740	NM_001103252.1	-4.14	0.001
ZNF839	XM_015459373.1	2.42	0.030
ZNF865	XM_010815504.2	-3.20	0.023

Genes are differentially expressed ($\log_2$ fold change ≥ 2 or ≤ -2 and a $P \leq 0.05$) in oocytes collected from bacteria infused heifers compared to heifers infused with vehicle medium at day 60 relative to infusion.

Supplemental Table 6. Canonical pathways overrepresented in oocytes collected from bacteria infused at day 4.

**Canonical Pathways**	**-log (*P*-value)**	**Z-score**	**Differentially expressed genes in pathway**
Hepatic Fibrosis / Hepatic Stellate Cell Activation	7.08	na	*ACTA2, CCL2, CCN2, CCR5, CD14, COL1A1, COL1A2, COL6A6, IFNAR2, IGFBP5, MET, SERPINE1, TGFB1, TNFRSF1B*
Interferon Signaling	5.33	-2.449	*IFIT1, IFITM1, IFNAR2, ISG15, MX1, OAS1*
Inhibition of Matrix Metalloproteases	3.95	na	*MMP19, SDC1, SDC2, THBS2, TIMP3*
Granulocyte Adhesion and Diapedesis	3.41	na	*C5AR1, CCL2, CLDN23, CLDN5, CXCL16, MMP19, SDC1, SDC2, TNFRSF1B*
Leukocyte Extravasation Signaling	3.07	-1.89	*ACTA2, ARHGAP35, CLDN23, CLDN5, DLC1, MMP19, PIK3R3, RAC2, TIMP3*
Coagulation System	3.06	1	*PLAT, PLG, SERPINC1, SERPINE1*
Glucocorticoid Receptor Signaling	2.99	na	*CCL2, FCGR1A, KRT1, KRT18, KRT72, KRT8, PCK2, PIK3R3, POLR2K, SERPINE1, SRA1, TGFB1*
HMGB1 Signaling	2.97	-2.449	*CCL2, PIK3R3, PLAT, RAC2, RND3, SERPINE1, TGFB1, TNFRSF1B*
Glioma Invasiveness Signaling	2.63	0	*PIK3R3, PLG, RAC2, RND3, TIMP3*
Apelin Cardiac Fibroblast Signaling Pathway	2.56	na	*CCN2, SERPINE1, TGFB1*
Atherosclerosis Signaling	2.36	na	*APOE, CCL2, COL1A1, COL1A2, TGFB1, TNFRSF12A*
Phagosome Formation	2.32	na	*C5AR1, FCGR1A, PIK3R3, RAC2, RND3, TLR6*
Germ Cell-Sertoli Cell Junction Signaling	2.24	na	*ACTA2, GSN, MAP3K15, PIK3R3, RAC2, RND3, TGFB1*
Factors Promoting Cardiogenesis in Vertebrates	2.23	na	*BMP2, BMPR1B, CCNE1, SMAD9, TGFB1*
Fcγ Receptor-mediated Phagocytosis in Macrophages and Monocytes	2.21	-2.236	*ACTA2, FCGR1A, PIK3R3, PLD2, RAC2*
TGF-β Signaling	2.17	-2	*BMP2, BMPR1B, SERPINE1, SMAD9, TGFB1*

Dendritic Cell Maturation	2.1	-2.646	*CD1D, COL1A1, COL1A2, FCGR1A, PIK3R3, TNFRSF1B, TYROBP*
Inhibition of Angiogenesis by TSP1	2.08	na	*SDC1, SDC2, TGFB1*
Endocannabinoid Cancer Inhibition Pathway	2.05	0.816	*AKT1S1, ATF3, CCNE1, PIK3R3, PRKAR1B, SNAI2*
Production of Nitric Oxide and Reactive Oxygen Species in Macrophages	2.04	-1.633	*APOE, MAP3K15, PIK3R3, RAC2, RND3, SIRPA, TNFRSF1B*
Guanosine Nucleotides Degradation III	2.04	na	*NT5C, NT5M*
IGF-1 Signaling	2.01	na	*CCN1, CCN2, IGFBP5, PIK3R3, PRKAR1B*
ILK Signaling	2	-2.236	*ACTA2, BMP2, KRT18, PIK3R3, RAC2, RND3, SNAI2*
Agranulocyte Adhesion and Diapedesis	2	na	*ACTA2, C5AR1, CCL2, CLDN23, CLDN5, CXCL16, MMP19*
RAR Activation	1.98	na	*BMP2, CITED2, PIK3R3, PRKAR1B, SMAD9, SRA1, TGFB1*
Complement System	1.98	na	*C1QC, C5AR1, C8B*
Urate Biosynthesis/Inosine 5'-phosphate Degradation	1.97	na	*NT5C, NT5M*
Role of Pattern Recognition Receptors in Recognition of Bacteria and Viruses	1.9	-2	*C1QC, C5AR1, OAS1, PIK3R3, TGFB1, TLR6*
Adenosine Nucleotides Degradation II	1.84	na	*NT5C, NT5M*
Intrinsic Prothrombin Activation Pathway	1.83	na	*COL1A1, COL1A2, SERPINC1*
Role of Hypercytokinemia/hyperchemokinemia in the Pathogenesis of Influenza	1.8	na	*CCL2, CCR5, IL9*
Osteoarthritis Pathway	1.78	-1.342	*ANXA2, BMP2, SLC39A8, SMAD9, TGFB1, TIMP3, TNFRSF1B*
LXR/RXR Activation	1.77	0.447	*AHSG, APOE, CCL2, CD14, TNFRSF1B*

Tight Junction Signaling	1.74	na	*ACTA2, CLDN23, CLDN5, PRKAR1B, TGFB1, TNFRSF1B*
RhoA Signaling	1.74	-1.342	*ACTA2, ARHGAP35, CDC42EP1, DLC1, RND3*
Actin Cytoskeleton Signaling	1.71	-1.342	*ACTA2, ARHGAP35, CD14, FGF20, GSN, PIK3R3, RAC2*
IL-6 Signaling	1.7	-2	*CD14, COL1A1, PIK3R3, TNFAIP6, TNFRSF1B*
Role of Osteoblasts, Osteoclasts and Chondrocytes in Rheumatoid Arthritis	1.7	na	*BMP2, COL1A1, GSN, PIK3R3, SMAD9, TGFB1, TNFRSF1B*
Differential Regulation of Cytokine Production in Macrophages and T Helper Cells by IL-17A and IL-17F	1.69	na	*CCL2, IL9*
Purine Nucleotides Degradation II (Aerobic)	1.69	na	*NT5C, NT5M*
BMP signaling pathway	1.68	-2	*BMP2, BMPR1B, PRKAR1B, SMAD9*
DNA damage-induced 14-3-3σ Signaling	1.65	na	*CCNE1, RAD1*
Molecular Mechanisms of Cancer	1.62	na	*BMP2, BMPR1B, CCNE1, PIK3R3, PRKAR1B, RAC2, RND3, SMAD9, STK36, TGFB1*
NF-κB Signaling	1.61	-1.633	*BMP2, BMPR1B, PIK3R3, TLR6, TNFAIP3, TNFRSF1B*
Glycine Biosynthesis III	1.61	na	*AGXT2*
Cardiomyocyte Differentiation via BMP Receptors	1.61	na	*BMP2, BMPR1B*
Sertoli Cell-Sertoli Cell Junction Signaling	1.55	na	*ACTA2, CLDN23, CLDN5, MAP3K15, PRKAR1B, SPTA1*
Th2 Pathway	1.55	-0.447	*CCR5, IL9, MAF, PIK3R3, TGFB1*
Regulation of Actin-based Motility by Rho	1.54	na	*ACTA2, GSN, RAC2, RND3*
Differential Regulation of Cytokine Production in Intestinal Epithelial	1.49	na	*CCL2, IL9*

Cells by IL-17A and IL-17F			
p53 Signaling	1.48	1	*JMY, PIK3R3, SNAI2, TP63*
Clathrin-mediated Endocytosis Signaling	1.48	na	*ACTA2, APOE, DNM3, FGF20, MET, PIK3R3*
Regulation of the Epithelial-Mesenchymal Transition Pathway	1.48	na	*EGR1, FGF20, MET, PIK3R3, SNAI2, TGFB1*
MSP-RON Signaling Pathway	1.44	na	*ACTA2, CCL2, PIK3R3*
Inosine-5'-phosphate Biosynthesis II	1.44	na	*PAICS*
Semaphorin Signaling in Neurons	1.42	na	*MET, RAC2, RND3*
Colorectal Cancer Metastasis Signaling	1.41	-2.236	*MMP19, PIK3R3, PRKAR1B, RAC2, RND3, TGFB1, TLR6*
PCP pathway	1.4	na	*CTHRC1, JUNB, NDP*
Autophagy	1.4	na	*CTSH, CTSS, STX17*
Antiproliferative Role of TOB in T Cell Signaling	1.39	na	*CCNE1, TGFB1*
NAD Salvage Pathway II	1.39	na	*NT5C, NT5M*
Apelin Liver Signaling Pathway	1.39	na	*COL1A1, COL1A2*
Role of Macrophages, Fibroblasts and Endothelial Cells in Rheumatoid Arthritis	1.39	na	*C5AR1, CCL2, CEBPD, FCGR1A, PIK3R3, TGFB1, TLR6, TNFRSF1B*
Pancreatic Adenocarcinoma Signaling	1.33	na	*CCNE1, PIK3R3, PLD2, TGFB1*
Arsenate Detoxification I (Glutaredoxin)	1.32	na	*GLRX2*
Melatonin Degradation II	1.32	na	*MAOA*
AMPK Signaling	1.31	-1	*AKT1S1, CHRNA5, MLYCD, PCK2, PIK3R3, PRKAR1B*
Cell Cycle: G1/S Checkpoint Regulation	1.3	na	*CCNE1, NRG1, TGFB1*
HGF Signaling	1.3	na	*ELF4, MAP3K15, MET, PIK3R3*

Supplemental Table 7. Canonical pathways overrepresented in oocytes collected from bacteria infused at day 60.

**Canonical Pathways**	**-log (*P*-value)**	**Z-score**	**Differentially expressed genes in pathway**
FXR/RXR Activation	4.14	na	*APOE, CLU, CYP19A1, CYP27A1, IL33, LPL, PON1, SAA1, SLC27A5*
Interferon Signaling	3.86	2.000	*IFI6, IRF9, ISG15, MX1, OAS1*
LXR/RXR Activation	3.51	0.378	*APOE, CLU, IL33, LDLR, LPL, PON1, SAA1, TNFRSF1A*
Bile Acid Biosynthesis, Neutral Pathway	3.16	na	*CYP27A1, SCP2, SLC27A5*
Fatty Acid β-oxidation I	3	1	*ACADM, EHHADH, SCP2, SLC27A5*
Chondroitin Sulfate Degradation (Metazoa)	2.88	na	*GALNS, HEXD, HYAL4*
Intrinsic Prothrombin Activation Pathway	2.56	1	*COL1A1, F11, KLK4, PROS1*
Atherosclerosis Signaling	2.09	na	*APOE, CLU, COL1A1, IL33, LPL, PON1*
IL-6 Signaling	2.06	2.449	*COL1A1, CYP19A1, FOS, IL33, TNFAIP6, TNFRSF1A*
Retinoic acid Mediated Apoptosis Signaling	2.00	2.000	*IFNW1, PARP10, PARP3, TNFRSF10A*
Acute Phase Response Signaling	1.88	na	*C1S, FOS, IL33, NR3C1, SAA1, SERPING1, TNFRSF1A*
Xanthine and Xanthosine Salvage	1.85	na	*PNP*
Sumoylation Pathway	1.83	-1.000	*FOS, NR3C1, RHOQ, RND3, TDG*
Production of Nitric Oxide and Reactive Oxygen Species in Macrophages	1.76	1.890	*APOE, CLU, FOS, PON1, RHOQ, RND3, TNFRSF1A*
ILK Signaling	1.73	1.890	*FOS, MYH3, RHOQ, RND3, SNAI2, TNFRSF1A, VEGFD*
Agranulocyte Adhesion and Diapedesis	1.73	na	*CD99, IL33, ITGA2, MYH3, SDC4, TNFRSF1A, XCL1*
γ-linolenate Biosynthesis II (Animals)	1.64	na	*FADS2, SLC27A5*

Dermatan Sulfate Degradation (Metazoa)	1.64	na	*HEXD, HYAL4*
Guanine and Guanosine Salvage I	1.56	na	*PNP*
L-glutamine Biosynthesis II (tRNA-dependent)	1.56	na	*QRSL1*
Adenine and Adenosine Salvage I	1.56	na	*PNP*
Tec Kinase Signaling	1.54	2.449	*FOS, FRK, ITGA2, RHOQ, RND3, TNFRSF10A*
Tight Junction Signaling	1.51	na	*FOS, JAM2, MPP5, MYH3, SYMPK, TNFRSF1A*
Death Receptor Signaling	1.41	2.000	*PARP10, PARP3, TNFRSF10A, TNFRSF1A*
Granulocyte Adhesion and Diapedesis	1.39	na	*CD99, IL33, ITGA2, SDC4, TNFRSF1A, XCL1*
Role of Lipids/Lipid Rafts in the Pathogenesis of Influenza	1.39	na	*IFNW1, RSAD2*

Supplemental Table 8. Canonical pathways overrepresented in control oocytes comparing day 4 and day 60 collection.

**Canonical Pathways**	**-log (*P*-value)**	**z-score**	**Molecules**
EIF2 Signaling	11.6	-4.382	*ACTA2, ACTB, ACTC1, ACTG2, AGO4, ATF3, EIF2B5, EIF4A2, HSPA5, MYC, MYCN, PABPC1, RAP2A, RPL10, RPL10A, RPL11, RPL12, RPL13A, RPL14, RPL18A, Rpl22l1, RPL23, RPL23A, RPL24, RPL29, RPL3, RPL30, RPL32, RPL35A, RPL36A, RPL37, RPL37A, RPL7, RPL8, RPLP0, RPS11, RPS12, RPS14, RPS17, RPS18, RPS19, RPS2, RPS20, RPS24, RPS25, RPS26, RPS27, RPS29, RPS3, RPS3A, RPS5, RPS6, RPS8, RPS9, RPSA, RRAS, VEGFA*
Hepatic Fibrosis / Hepatic Stellate Cell Activation	8.15	na	*ACTA2, CCL2, CCL5, CCN2, CCR5, CD14, COL1A1, COL1A2, COL27A1, COL4A1, COL4A3, COL4A6, COL6A1, COL6A6, ECE1, FAS, IFNAR2, IFNGR2, IGFBP4, IGFBP5, IL1A, IL1B, IL4R, IL6R, KLF6, LY96, MET, MMP9, MYH11, MYL6, MYL9, PDGFC, PDGFRA, PGF, SERPINE1, SMAD4, TGFB1, TIMP1, TIMP2, TNFRSF1A, TNFRSF1B, VEGFA, VEGFB, VEGFC*
Glycolysis I	7.88	-3.207	*ALDOA, ALDOB, ALDOC, ENO1, ENO3, FBP1, GAPDH, PFKL, PFKP, PGAM1, PGK1, PKLR, PKM, TPI1*
Aryl Hydrocarbon Receptor Signaling	6.51	-1.528	*ALDH16A1, ALDH18A1, ALDH1L1, ALDH5A1, ALDH9A1, ARNT, CCND2, CCND3, CDK4, CDK6, CTSD, ESR1, FAS, FOS, GSTA1, GSTA3, GSTM1, GSTM4, GSTO1, GSTP1, HSPB1, IL1A, IL1B, JUN, MCM7, MYC, NCOA7, NFE2L2, NQO2, RARA, RXRB, TGFB1, TGM2, TP53*
mTOR Signaling	6.03	-2.668	*EIF4A2, EIF4B, EIF4EBP1, FNBP1, HIF1A, HMOX1, PDGFC, PGF, PLD2, PLD3, PPP2R5A, PRKAB1,*

			PRKCB, RAC2, RAP2A, RHOB, RHOQ, RND2, RND3, RPS11, RPS12, RPS14, RPS17, RPS18, RPS19, RPS2, RPS20, RPS24, RPS25, RPS26, RPS27, RPS29, RPS3, RPS3A, RPS5, RPS6, RPS8, RPS9, RPSA, RRAS, VEGFA, VEGFB, VEGFC
Death Receptor Signaling	5.63	-3.128	*ACTA2, ACTB, ACTC1, ACTG1, ACTG2, ARHGDIB, BIRC3, CASP7, DFFA, DFFB, DIABLO, FAS, GAS2, HSPB1, MAP3K5, NFKBID, PARP10, PARP14, PARP3, PARP8, TNFRSF10A, TNFRSF1A, TNFRSF1B, TNFRSF21*
ILK Signaling	5.10	-4.116	*ACTA2, ACTB, ACTC1, ACTG1, ACTG2, BMP2, CREB3, CTNNB1, FLNA, FNBP1, FOS, HIF1A, ILK, ITGB8, JUN, KRT18, MAPK10, MMP9, MYC, MYH11, MYL6, MYL9, PARVA, PDGFC, PGF, PPP2R5A, PTEN, RAC2, RHOB, RHOQ, RND2, RND3, SNAI2, TNFRSF1A, VEGFA, VEGFB, VEGFC, VIM*
Interferon Signaling	4.90	-3.464	*BAK1, IFI35, IFI6, IFIT1, IFITM1, IFNAR2, IFNGR2, IRF9, ISG15, MX1, OAS1, PSMB8, TAP1*
Agrin Interactions at Neuromuscular Junction	4.60	-3.000	*ACTA2, ACTB, ACTC1, ACTG1, ACTG2, AGRN, CHRNA1, ERBB2, ITGA2, ITGA5, ITGA6, JUN, LAMB1, MAPK10, NRG1, PKLR, RAC2, RAP2A, RRAS, UTRN*
Inhibition of Matrix Metalloproteases	4.47	0.905	*MMP14, MMP16, MMP17, MMP19, MMP23B, MMP9, SDC1, SDC2, THBS2, TIMP1, TIMP2, TIMP3, TIMP4*
Inhibition of Angiogenesis by TSP1	4.45	-1.667	*CD36, CD47, GUCY1B1, JUN, MAPK10, MMP9, SDC1, SDC2, TGFB1, THBS1, TP53, VEGFA*
Agranulocyte Adhesion and Diapedesis	4.34	na	*ACTA2, ACTB, ACTC1, ACTG1, ACTG2, CCL2, CCL22, CCL25, CCL3L3, CCL5, CCL8, CD99, CLDN20, CXCL10, CXCL12, CXCL13, CXCL16, CXCL2, IL1A, IL1B, IL33, ITGA2, ITGA5, ITGA6,*

			MMP14, MMP16, MMP17, MMP19, MMP23B, MMP9, MYH11, MYL6, MYL9, SDC4, TNFRSF1A, XCL1
LXR/RXR Activation	4.31	0.853	*AHSG, APOA1, APOE, APOM, CCL2, CD14, CD36, CLU, FASN, IL1A, IL1B, IL33, IRF3, LPL, LY96, LYZ, MMP9, MSR1, PCYOX1, RXRB, S100A8, SCD, SERPINF2, TLR3, TNFRSF1A, TNFRSF1B*
Gluconeogenesis I	4.16	-3.162	*ALDOA, ALDOB, ALDOC, ENO1, ENO3, FBP1, GAPDH, MDH1B, PGAM1, PGK1*
Proline Biosynthesis I	4.11	-2.000	*ALDH18A1, PYCR1, PYCR2, PYCR3*
RhoGDI Signaling	3.84	3.780	*ACTA2, ACTB, ACTC1, ACTG1, ACTG2, ARHGAP1, ARHGAP6, ARHGDIB, ARHGEF17, ARPC5, CD44, CDH2, CDH6, DLC1, ESR1, FNBP1, GNG10, GNG2, GNG5, GNG7, ITGA2, ITGA5, MYL6, MYL9, MYLPF, PI4KA, PIP5K1A, RAC2, RHOB, RHOQ, RND2, RND3, ROCK2*
Regulation of eIF4 and p70S6K Signaling	3.82	-1.633	*AGO4, EIF2B5, EIF4A2, EIF4EBP1, ITGA2, ITGA5, PABPC1, PPP2R5A, RAP2A, RPS11, RPS12, RPS14, RPS17, RPS18, RPS19, RPS2, RPS20, RPS24, RPS25, RPS26, RPS27, RPS29, RPS3, RPS3A, RPS5, RPS6, RPS8, RPS9, RPSA, RRAS*
Atherosclerosis Signaling	3.71	na	*APOA1, APOE, APOM, CCL2, CD36, CLU, COL1A1, COL1A2, CXCL12, IL1A, IL1B, IL33, LPL, LYZ, MMP9, MSR1, PCYOX1, PDGFC, PLA2G16, PLA2G4F, PLA2G7, PNPLA8, S100A8, TGFB1, TNFRSF12A*
Leukocyte Extravasation Signaling	3.67	-3.182	*ACTA2, ACTB, ACTC1, ACTG1, ACTG2, ARHGAP1, ARHGAP6, CD44, CD99, CLDN20, CTNNB1, CXCL12, CYBA, DLC1, EDIL3, ITGA2, ITGA5, ITGA6, JAM2, MAPK10, MMP14, MMP16, MMP17, MMP19, MMP23B, MMP9, MYL6, PRKCB, RAC2, ROCK2,*

			TEC, TIMP1, TIMP2, TIMP3, TIMP4
Colorectal Cancer Metastasis Signaling	3.66	-4.867	*CTNNB1, FNBP1, FOS, FZD1, FZD10, FZD9, GNG10, GNG2, GNG5, GNG7, IL6R, JUN, MAPK10, MMP14, MMP16, MMP17, MMP19, MMP23B, MMP9, MYC, PDGFC, PGF, PRKAR1B, PRKAR2B, RAC2, RAP2A, RHOB, RHOQ, RND2, RND3, RRAS, SMAD4, TGFB1, TLR3, TLR6, TNFRSF1A, TP53, VEGFA, VEGFB, VEGFC, WNT3, WNT5B*
Bladder Cancer Signaling	3.65	-1.633	*CDK4, ERBB2, FGF11, FGF20, FGF23, MMP14, MMP16, MMP17, MMP19, MMP23B, MMP9, MYC, PDGFC, PGF, RAP2A, RRAS, THBS1, TP53, VEGFA, VEGFB, VEGFC*
Apoptosis Signaling	3.52	-1.964	*BAK1, BIRC3, CAPN2, CAPN5, CAPN6, CAPNS1, CASP7, DFFA, DFFB, DIABLO, ENDOG, FAS, GAS2, MAP3K5, MCL1, NFKBID, RAP2A, RRAS, TNFRSF1A, TNFRSF1B, TP53*
HIF1α Signaling	3.46	na	*ARNT, ELOB, EPO, HIF1A, JUN, MAPK10, MMP14, MMP16, MMP17, MMP19, MMP23B, MMP9, P4HTM, PDGFC, PGF, RAP2A, RRAS, SLC2A1, SLC2A3, TP53, VEGFA, VEGFB, VEGFC*
Osteoarthritis Pathway	3.41	-4.131	*ANKH, ANXA2, ANXA5, BMP2, CASP7, CASQ1, CREB3, CTNNB1, DCN, FZD1, FZD10, FZD9, HIF1A, HTRA1, IHH, IL1B, ITGA2, ITGA5, MMP9, PDGFC, PGF, PRKAB1, RARRES2, S100A8, SDC4, SLC39A8, SMAD4, SPHK1, SPP1, TGFB1, TIMP3, TNFRSF1A, TNFRSF1B, VEGFA, VEGFB, VEGFC*
Regulation of Actin-based Motility by Rho	3.40	-3.441	*ACTA2, ACTB, ACTC1, ACTG2, ARPC5, FNBP1, GSN, ITGA2, ITGA5, MYL6, MYL9, MYLPF, PFN2, PI4KA, PIP5K1A, RAC2, RHOB, RHOQ, RND2, RND3*

Induction of Apoptosis by HIV1	3.39	-2.138	*BAK1, BIRC3, DFFA, DFFB, DIABLO, FAS, MAP3K5, MAPK10, NFKBID, SLC25A10, SLC25A3, SLC25A6, TNFRSF1A, TNFRSF1B, TP53*
Sertoli Cell-Sertoli Cell Junction Signaling	3.23	na	*ACTA2, ACTB, ACTC1, ACTG1, ACTG2, CLDN20, CTNNB1, GUCY1B1, ILK, ITGA2, ITGA5, JAM2, JUN, JUP, MAP3K15, MAP3K2, MAP3K20, MAP3K5, MAPK10, NECTIN1, PRKAR1B, PRKAR2B, PTEN, RAP2A, RRAS, SPTA1, TNFRSF1A, TUBA1A, TUBB, TUBB2B, TUBB6, YBX3*
Granulocyte Adhesion and Diapedesis	3.20	na	*CCL2, CCL22, CCL25, CCL3L3, CCL5, CCL8, CD99, CLDN20, CXCL10, CXCL12, CXCL13, CXCL16, CXCL2, IL1A, IL1B, IL33, ITGA2, ITGA5, ITGA6, MMP14, MMP16, MMP17, MMP19, MMP23B, MMP9, SDC1, SDC2, SDC4, TNFRSF1A, TNFRSF1B, XCL1*
Oleate Biosynthesis II (Animals)	3.18	-2.449	*FADS1, FADS2, SCD, SCD5, UFSP1, UFSP2*
Sucrose Degradation V (Mammalian)	3.18	-2.236	*ALDOA, ALDOB, ALDOC, GALM, TPI1*
Antigen Presentation Pathway	3.16	na	*B2M, HLA-B, HLA-DMB, HLA-DQA1, HLA-DQB1, HLA-DRA, PSMB8, PSMB9, TAP1, TAP2, TAPBP*
Germ Cell-Sertoli Cell Junction Signaling	3.13	na	*ACTA2, ACTB, ACTC1, ACTG1, ACTG2, CDH2, CTNNB1, FNBP1, GSN, ILK, ITGA2, ITGA6, JUP, MAP3K15, MAP3K2, MAP3K5, MAPK10, RAC2, RAP2A, RHOB, RHOQ, RND2, RND3, RRAS, TGFB1, TNFRSF1A, TUBA1A, TUBB, TUBB2B, TUBB6*
LPS/IL-1 Mediated Inhibition of RXR Function	2.99	-0.905	*ACOX3, ACSBG1, ALDH16A1, ALDH18A1, ALDH1L1, ALDH5A1, ALDH9A1, APOE, CD14, CES2, FABP4, FMO4, FMO5, GSTA1, GSTA3, GSTM1, GSTM4, GSTO1, GSTP1, HS3ST5, HS6ST1, HS6ST2, IL1A, IL1B, IL33, JUN, LY96,*

			MAOA, MAOB, PAPSS2, RARA, SCARB1, SOD3, TNFRSF1A, TNFRSF1B, XPO1
IL-8 Signaling	2.99	-4.950	*ANGPT1, ANGPT2, CCND2, CCND3, EIF4EBP1, FNBP1, FOS, GNG10, GNG2, GNG5, GNG7, HMOX1, ITGAV, JUN, MAPK10, MMP9, MYL9, PDGFC, PGF, PLD2, PLD3, PRKCB, RAC2, RAP2A, RHOB, RHOQ, RND2, RND3, ROCK2, RRAS, VEGFA, VEGFB, VEGFC*
Caveolar-mediated Endocytosis Signaling	2.98	na	*ACTA2, ACTB, ACTC1, ACTG1, ACTG2, B2M, CAVIN1, CD48, FLNA, HLA-B, ITGA2, ITGA5, ITGA6, ITGAV, ITGB8, MAP3K2*
Mechanisms of Viral Exit from Host Cells	2.96	na	*ACTA2, ACTB, ACTC1, ACTG1, ACTG2, CHMP4C, NEDD4, PRKCB, SH3GL3, VPS36, XPO1*
Signaling by Rho Family GTPases	2.87	-3.889	*ACTA2, ACTB, ACTC1, ACTG1, ACTG2, ARHGEF17, ARPC5, CDC42EP1, CDH2, CDH6, FNBP1, FOS, GNG10, GNG2, GNG5, GNG7, ITGA2, ITGA5, JUN, MAP3K20, MAPK10, MYL6, MYL9, MYLPF, NEDD4, PARD3, PI4KA, PIP5K1A, PKN1, RAC2, RHOB, RHOQ, RND2, RND3, ROCK2, SEPT10, STMN1, VIM*
Glioma Invasiveness Signaling	2.85	-1.291	*CD44, FNBP1, ITGAV, MMP9, PLG, RAC2, RAP2A, RHOB, RHOQ, RND2, RND3, RRAS, TIMP1, TIMP2, TIMP3, TIMP4*
Axonal Guidance Signaling	2.79	na	*ADAM20, ADAM22, ADAM9, ADAMTS3, ADAMTS9, AOPEP, ARPC5, BMP2, BMP4, CXCL12, ECE2, EFNA5, ERBB2, FZD1, FZD10, FZD9, GNG10, GNG2, GNG5, GNG7, ITGA2, ITGA5, MET, MMP14, MMP16, MMP17, MMP23B, MMP9, MYL6, MYL9, MYLPF, NGF, NTNG1, NTRK1, PAPPA2, PDGFC, PFN2, PGF, PLCB4, PLXNA2, PLXNB1, PPP3CA, PRKAR1B, PRKAR2B, PRKCB, PSMD14, RAC2, RAP2A,*

			RGS3, ROCK2, RRAS, RTN4, SDC2, SEMA4F, SLIT1, SRGAP2, STK36, TUBA1A, TUBB, TUBB2B, TUBB6, VEGFA, VEGFB, VEGFC, WNT3, WNT5B
NRF2-mediated Oxidative Stress Response	2.73	-3.500	*ACTA2, ACTB, ACTC1, ACTG1, ACTG2, DNAJB9, ENC1, EPHX1, FOS, FTH1, GCLC, GSTA1, GSTA3, GSTM1, GSTM4, GSTO1, GSTP1, HERPUD1, HMOX1, JUN, JUNB, MAP3K5, NFE2L2, NQO2, PPIB, PRKCB, RAP2A, RRAS, SCARB1, SOD3, TXN*
Tight Junction Signaling	2.69	na	*ACTA2, ACTB, ACTC1, ACTG1, ACTG2, BET1L, CDK4, CLDN20, CPSF6, CTNNB1, FOS, JAM2, JUN, MPP5, MYH11, MYL6, MYL9, NECTIN1, PPP2R5A, PRKAR1B, PRKAR2B, PTEN, RAB13, STX4, TGFB1, TNFRSF1A, TNFRSF1B, YBX3*
RhoA Signaling	2.62	-2.558	*ACTA2, ACTB, ACTC1, ACTG1, ACTG2, ARHGAP1, ARHGAP6, ARPC5, CDC42EP1, DLC1, MYL6, MYL9, MYLPF, NEDD4, PFN2, PI4KA, PIP5K1A, PKN1, RHPN1, RND3, ROCK2, SEPT10*
Arsenate Detoxification I (Glutaredoxin)	2.51	na	*GLRX2, GSTO1, PNP*
Heme Degradation	2.51	na	*BLVRA, BLVRB, HMOX1*
FXR/RXR Activation	2.49	na	*AHSG, APOA1, APOE, APOM, CLU, CYP19A1, CYP27A1, FASN, FBP1, G6PC3, IL1A, IL1B, IL33, LPL, MAPK10, PCYOX1, PKLR, RARA, SCARB1, SDC1, SERPINF2, VLDLR*
Neuroinflammation Signaling Pathway	2.42	-5.284	*B2M, BIRC3, CCL2, CCL5, CD200, CREB3, CTNNB1, CXCL10, CXCL12, FAS, FOS, FZD1, GABBR1, GABRD, GABRE, GRIN3A, HLA-B, HLA-DMB, HLA-DQA1, HLA-DQB1, HLA-DRA, HMOX1, IFNA16, IFNGR2, IL1B, IL6R, IRF3, JUN, KLK1, MAPK10, MFGE8, MMP9, NFE2L2, NGF, PLA2G4F, PPP3CA, PYCARD,*

			SLC6A1, SYK, TGFB1, TLR3, TLR6, TNFRSF1A
PCP pathway	2.41	-2.309	*CTHRC1, FZD1, FZD10, FZD9, JUN, JUNB, MAPK10, NDP, PFN2, ROCK2, ROR2, WNT3, WNT5B*
Coagulation System	2.38	1.667	*F13B, F8, KLKB1, PLAT, PLG, PROS1, SERPINA5, SERPINE1, SERPINF2*
Graft-versus-Host Disease Signaling	2.37	na	*FAS, FCER1G, HLA-B, HLA-DMB, HLA-DQA1, HLA-DQB1, HLA-DRA, IL1A, IL1B, IL33, PRF1*
Clathrin-mediated Endocytosis Signaling	2.34	na	*ACTA2, ACTB, ACTC1, ACTG1, ACTG2, AP1G1, AP1G2, AP1S2, APOA1, APOE, APOM, ARPC5, CLU, FGF11, FGF20, FGF23, ITGA5, ITGB8, LYZ, MET, PCYOX1, PDGFC, PGF, PPP3CA, S100A8, SH3GL3, UBD, VEGFA, VEGFB, VEGFC*
Glutathione Redox Reactions I	2.28	-2.646	*GPX1, GPX3, GPX7, GPX8, GSTA1, GSTM1, GSTP1*
TCA Cycle II (Eukaryotic)	2.28	-2.646	*IDH3A, IDH3B, IDH3G, MDH1B, SDHAF4, SDHD, SUCLG1*
Virus Entry via Endocytic Pathways	2.26	na	*ACTA2, ACTB, ACTC1, ACTG1, ACTG2, AP1G1, AP1G2, AP1S2, B2M, FLNA, HLA-B, ITGA2, ITGA5, ITGA6, ITGB8, PRKCB, RAC2, RAP2A, RRAS*
Tec Kinase Signaling	2.21	-3.710	*ACTA2, ACTB, ACTC1, ACTG1, ACTG2, FAS, FCER1G, FNBP1, FOS, GNG10, GNG2, GNG5, GNG7, HCK, ITGA2, ITGA5, MAPK10, PRKCB, RAC2, RHOB, RHOQ, RND2, RND3, TEC, TNFRSF10A, TNFRSF21*
Integrin Signaling	2.21	-4.642	*ACTA2, ACTB, ACTC1, ACTG1, ACTG2, ARPC5, CAPN2, CAPN5, CAPN6, CAPNS1, FNBP1, GSN, ILK, ITGA2, ITGA5, ITGA6, ITGAV, ITGB8, MYL9, PARVA, PFN2, PTEN, RAC2, RAP2A, RHOB, RHOQ, RND2, RND3, RRAS, TLN1, TSPAN4, TSPAN6*
Sumoylation Pathway	2.14	0.000	*AR, ARHGDIB, FAS, FNBP1, FOS, ISG20, JUN, MAP3K5, MAPK10, RAC2, RHOB, RHOQ, RND2,*

			RND3, SMAD4, SUMO3, TDG, TP53
T Helper Cell Differentiation	2.13	na	*FCER1G, HLA-B, HLA-DMB, HLA-DQA1, HLA-DQB1, HLA-DRA, IFNGR2, IL10RB, IL2RG, IL4R, IL6R, TGFB1, TNFRSF1A, TNFRSF1B*
Crosstalk between Dendritic Cells and Natural Killer Cells	2.08	na	*ACTA2, ACTB, ACTC1, ACTG1, ACTG2, CD83, FAS, FSCN1, HLA-B, HLA-DRA, IL15RA, IL2RG, PRF1, TLN1, TLR3, TNFRSF1B*
Sulfate Activation for Sulfonation	2.05	na	*PAPSS1, PAPSS2*
Glycine Degradation (Creatine Biosynthesis)	2.05	na	*GAMT, GATM*
Adenine and Adenosine Salvage I	2.05	na	*APRT, PNP*
PFKFB4 Signaling Pathway	2.03	-1.897	*CREB3, FBP1, GCK, HK1, HK2, PRKAR1B, PRKAR2B, TGFB1, TKT, TP53*
Role of Osteoblasts, Osteoclasts and Chondrocytes in Rheumatoid Arthritis	2.03	na	*BIRC3, BMP2, BMP4, COL1A1, CSNK1A1, CTNNB1, CTSK, FOS, FZD1, FZD10, FZD9, GSN, IL1A, IL1B, IL33, ITGA2, ITGA5, JUN, MAP3K5, MAPK10, MMP14, NFKBID, PPP3CA, SFRP4, SMAD4, SPP1, TGFB1, TNFRSF1A, TNFRSF1B, TNFSF11, WNT3, WNT5B*
Altered T Cell and B Cell Signaling in Rheumatoid Arthritis	2.03	na	*CXCL13, FAS, FCER1G, HLA-B, HLA-DMB, HLA-DQA1, HLA-DQB1, HLA-DRA, IL1A, IL1B, IL33, SPP1, TGFB1, TLR3, TLR6, TNFSF11*
Amyotrophic Lateral Sclerosis Signaling	2.02	-2.183	*BIRC3, CAPN2, CAPN5, CAPN6, CAPNS1, CASP7, GPX1, GRIA2, GRIN3A, PDGFC, PGF, PPP3CA, RNF19A, TP53, VEGFA, VEGFB, VEGFC*
Production of Nitric Oxide and Reactive Oxygen Species in Macrophages	1.97	-3.272	*APOA1, APOE, APOM, CLU, CYBA, FNBP1, FOS, IFNGR2, JUN, LYZ, MAP3K15, MAP3K2, MAP3K5, MAPK10, NFKBID, PCYOX1, PPP1R14A, PPP2R5A, PRKCB, RAC2, RHOB, RHOQ, RND2,*

			RND3, S100A8, SIRPA, TNFRSF1A, TNFRSF1B
Endoplasmic Reticulum Stress Pathway	1.97	na	*CASP7, HSPA5, MAP3K5, MBTPS1, MBTPS2, XBP1*
IL-10 Signaling	1.95	na	*BLVRA, BLVRB, CCR5, CD14, FOS, HMOX1, IL10RB, IL1A, IL1B, IL33, IL4R, JUN, NFKBID*
Remodeling of Epithelial Adherens Junctions	1.95	na	*ACTA2, ACTB, ACTC1, ACTG1, ACTG2, ARPC5, CTNNB1, MET, NME1, TUBA1A, TUBB, TUBB2B, TUBB6*
Ovarian Cancer Signaling	1.94	-3.051	*CD44, CDK4, CTNNB1, FSHR, FZD1, FZD10, FZD9, GJA1, MMP9, PDGFC, PGF, PRKAR1B, PRKAR2B, PTEN, RAP2A, RRAS, TP53, VEGFA, VEGFB, VEGFC, WNT3, WNT5B*
Cytotoxic T Lymphocyte-mediated Apoptosis of Target Cells	1.92	-1.633	*B2M, CASP7, DFFA, DFFB, FAS, FCER1G, HLA-B, PRF1*
Role of Macrophages, Fibroblasts and Endothelial Cells in Rheumatoid Arthritis	1.92	na	*CCL2, CCL5, CEBPD, CREB3, CSNK1A1, CTNNB1, CXCL12, FOS, FZD1, FZD10, FZD9, IL17RA, IL1A, IL1B, IL33, IL6R, JUN, MIF, MYC, NFKBID, PDGFC, PGF, PLCB4, PPP3CA, PRKCB, PRSS2, RAP2A, ROCK2, ROR2, RRAS, SFRP4, TGFB1, TLR3, TLR6, TNFRSF1A, TNFRSF1B, TNFSF11, VEGFA, VEGFB, VEGFC, WNT3, WNT5B*
VEGF Signaling	1.89	-3.357	*ACTA2, ACTB, ACTC1, ACTG1, ACTG2, ARNT, EIF2B5, HIF1A, PDGFC, PGF, PRKCB, RAP2A, ROCK2, RRAS, VEGFA, VEGFB, VEGFC*
Proline Biosynthesis II (from Arginine)	1.87	na	*PYCR1, PYCR2, PYCR3*
Arginine Degradation VI (Arginase 2 Pathway)	1.87	na	*PYCR1, PYCR2, PYCR3*
Polyamine Regulation in Colon Cancer	1.86	na	*AZIN1, CTNNB1, MYC, PSME1, PSME2, SAT2*
Pyrimidine Deoxyribonucleotides De Novo Biosynthesis I	1.86	-2.449	*AK5, AK7, CMPK2, NME1, NME3, NME6*

Fcγ Receptor-mediated Phagocytosis in Macrophages and Monocytes	1.85	-4.000	*ACTA2, ACTB, ACTC1, ACTG1, ACTG2, ARPC5, HCK, HMOX1, PIP5K1A, PLD2, PLD3, PRKCB, PTEN, RAC2, SYK, TLN1*
Unfolded protein response	1.85	na	*CEBPD, DNAJB9, ERO1B, HSPA5, MAP3K5, MBTPS1, MBTPS2, NFE2L2, OS9, P4HB, XBP1*
MIF Regulation of Innate Immunity	1.84	-1.000	*CD14, FOS, JUN, LY96, MAPK10, MIF, NFKBID, PLA2G4F, TP53*
Endocannabinoid Cancer Inhibition Pathway	1.81	2.132	*ATF3, CASP7, CASQ1, CCND2, CCND3, CREB3, CTNNB1, HIF1A, MYC, NUPR1, PDGFC, PGF, PRKAB1, PRKAR1B, PRKAR2B, ROCK2, SMPD1, SNAI2, VEGFA, VEGFB, VEGFC, VIM*
Epithelial Adherens Junction Signaling	1.79	na	*ACTA2, ACTB, ACTC1, ACTG1, ACTG2, ARPC5, CDH2, CTNNB1, JUP, MET, MYH11, MYL6, MYL9, NECTIN1, PARD3, PTEN, RAP2A, RRAS, SNAI2, TUBA1A, TUBB, TUBB2B, TUBB6*
FAK Signaling	1.77	na	*ACTA2, ACTB, ACTC1, ACTG1, ACTG2, CAPN2, CAPN5, CAPN6, CAPNS1, ITGA2, ITGA5, PTEN, RAP2A, RRAS, TLN1, TNS1*
Apelin Cardiac Fibroblast Signaling Pathway	1.77	1.633	*ANGPT2, CCN2, PRKAB1, SERPINE1, SPHK1, TGFB1*
Regulation of Cellular Mechanics by Calpain Protease	1.72	-2.828	*CAPN2, CAPN5, CAPN6, CAPNS1, CDK4, CDK6, CNGA3, ITGA2, ITGA5, RAP2A, RRAS, TLN1*
Pyrimidine Ribonucleotides De Novo Biosynthesis	1.71	-3.000	*AK5, AK7, CAD, CMPK2, CTPS2, DHX9, NME1, NME3, NME6*
Tumoricidal Function of Hepatic Natural Killer Cells	1.68	-1.342	*CASP7, DFFA, DFFB, ENDOG, FAS, PRF1*
Cell Cycle: G1/S Checkpoint Regulation	1.67	0.302	*CCND2, CCND3, CDK4, CDK6, CDKN2C, HDAC6, MYC, NRG1, RPL11, SMAD4, TGFB1, TP53*
NAD Salvage Pathway III	1.66	na	*NMNAT3, NMRK1, NMRK2*
Glycoaminoglycan-protein Linkage Region Biosynthesis	1.66	na	*B3GAT3, XYLT1, XYLT2*

D-myo-inositol (1, 3, 4)-trisphosphate Biosynthesis	1.66	-2.236	*INPPL1, ITPKA, MINPP1, PTEN, SEC16A*
Trehalose Degradation II (Trehalase)	1.6	na	*GCK, HK1*
5-aminoimidazole Ribonucleotide Biosynthesis I	1.6	na	*GART, PPAT*
IL-17A Signaling in Gastric Cells	1.59	-1.000	*CCL5, CXCL10, FOS, IL17RA, JUN, MAPK10*
Autophagy	1.59	na	*ATG4C, ATG7, CTSA, CTSD, CTSF, CTSH, CTSK, CTSS, CTSV, NBR1, WIPI1*
FAT10 Cancer Signaling Pathway	1.59	-2.333	*ACKR3, CTNNB1, EEF1A1, SMAD4, TGFB1, TNFRSF1A, TNFRSF1B, TP53, UBD*
Cardiac Hypertrophy Signaling (Enhanced)	1.58	-5.612	*CTF1, CTNNB1, EIF2B5, EIF4EBP1, FGF11, FGF20, FGF23, FGFRL1, FZD1, FZD10, FZD9, GNG2, GNG5, GNG7, HAND2, HDAC6, HSPB1, IL10RB, IL11RA, IL15RA, IL17RA, IL1A, IL1B, IL27RA, IL2RG, IL33, IL4R, IL6R, ITGA2, ITGA5, JUN, MAP3K15, MAP3K2, MAP3K20, MAP3K5, MAPK10, MPPE1, MYC, PDE7B, PKN1, PLCB4, PPP3CA, PRKAR1B, PRKAR2B, PRKCB, PTEN, RAP2A, ROCK2, RPS6, RRAS, RYR2, TG, TGFB1, TNFRSF1A, TNFRSF1B, TNFSF11, TNFSF4, WNT3, WNT5B*
IL-6 Signaling	1.58	-3.771	*CD14, COL1A1, CYP19A1, FOS, HSPB1, IL1A, IL1B, IL33, IL6R, JUN, MAPK10, MCL1, NFKBID, RAP2A, RRAS, TNFAIP6, TNFRSF1A, TNFRSF1B, VEGFA*
Bile Acid Biosynthesis, Neutral Pathway	1.55	-2.000	*AMACR, CYP27A1, HSD3B7, SCP2*
Type I Diabetes Mellitus Signaling	1.52	-2.333	*FAS, FCER1G, HLA-B, HLA-DMB, HLA-DQA1, HLA-DQB1, HLA-DRA, HSPD1, IFNGR2, IL1B, MAP3K5, MAPK10, NFKBID, PRF1, PTPRN, TNFRSF1A, TNFRSF1B*
NAD Salvage Pathway II	1.51	-0.816	*ACP4, NMNAT3, NMRK1, NMRK2, NT5C, NT5M*

Activation of IRF by Cytosolic Pattern Recognition Receptors	1.50	-1.508	*DDX58, IFIH1, IFNA16, IRF3, IRF9, ISG15, JUN, MAPK10, NFKBID, PIN1, PPIB*
TGF-β Signaling	1.46	-2.887	*AMH, BMP2, BMP4, FOS, INHA, INHBA, INHBB, JUN, PMEPA1, RAP2A, RRAS, SERPINE1, SMAD4, TGFB1, TGIF1*
Molecular Mechanisms of Cancer	1.45	na	*ARHGEF17, BAK1, BIRC3, BMP2, BMP4, CASP7, CCND2, CCND3, CDK10, CDK19, CDK4, CDK6, CDKN2C, CTNNB1, DHH, DIABLO, FAS, FNBP1, FOS, FZD1, FZD10, FZD9, HIF1A, IHH, ITGA2, ITGA5, JUN, MAP3K5, MAPK10, MYC, NFKBID, PLCB4, PRKAR1B, PRKAR2B, PRKCB, RAC2, RAP2A, RHOB, RHOQ, RND2, RND3, RRAS, SMAD4, STK36, TGFB1, TP53, WNT3, WNT5B*
Actin Nucleation by ARP-WASP Complex	1.45	-2.714	*ARPC5, FNBP1, ITGA2, ITGA5, RAC2, RAP2A, RHOB, RHOQ, RND2, RND3, ROCK2, RRAS*
Autoimmune Thyroid Disease Signaling	1.43	na	*FAS, FCER1G, HLA-B, HLA-DMB, HLA-DQA1, HLA-DQB1, HLA-DRA, PRF1, TG*
Salvage Pathways of Pyrimidine Ribonucleotides	1.43	-3.873	*ADPGK, AK5, AK7, APOBEC3B, CDK4, CDK6, CMPK2, CSNK1A1, NME1, NME3, NME6, PKN1, POMK, PYCR3, SGK1*
HMGB1 Signaling	1.42	-3.300	*CCL2, FNBP1, FOS, IFNGR2, IL1A, IL1B, IL33, JUN, MAPK10, PLAT, RAC2, RAP2A, RHOB, RHOQ, RND2, RND3, RRAS, SERPINE1, TGFB1, TNFRSF1A, TNFRSF1B, TNFSF11, TNFSF4*
Intrinsic Prothrombin Activation Pathway	1.40	-0.707	*COL1A1, COL1A2, F13B, F8, KLK1, KLK4, KLKB1, PROS1*
Pyrimidine Ribonucleotides Interconversion	1.40	-2.828	*AK5, AK7, CMPK2, CTPS2, DHX9, NME1, NME3, NME6*
Phospholipase C Signaling	1.39	-3.657	*ARHGEF17, BLNK, CREB3, FCER1G, FNBP1, GNG10, GNG2, GNG5, GNG7, HDAC6, HMOX1, ITGA2, ITGA5, MARCKS, MYL6, MYL9, MYLPF, PLA2G4F, PLCB4, PLD2, PLD3, PPP1R14A, PPP3CA,*

			PRKCB, RAC2, RAP2A, RHOB, RHOQ, RND2, RND3, RRAS, SYK, TGM2
RAR Activation	1.38	na	*ACTB, ACTL6B, BMP2, CRABP2, DHRS3, DUSP1, FOS, HLTF, JUN, MAP3K5, MAPK10, NR2F6, PHF10, PNRC1, PRKAR1B, PRKAR2B, PRKCB, PTEN, RARA, RDH13, RXRB, SMAD4, SMARCD3, SRA1, TGFB1, VEGFA*
IL-17A Signaling in Fibroblasts	1.38	na	*CCL2, CEBPD, FOS, IL17RA, JUN, NFKBID, NFKBIZ*
CXCR4 Signaling	1.37	-3.130	*CXCL12, EGR1, FNBP1, FOS, GNG10, GNG2, GNG5, GNG7, JUN, MAPK10, MYL6, MYL9, MYLPF, PLCB4, PRKCB, RAC2, RAP2A, RHOB, RHOQ, RND2, RND3, ROCK2, RRAS*
Role of Hypercytokinemia/hyperchemokinemia in the Pathogenesis of Influenza	1.35	na	*CCL2, CCL5, CCR5, CXCL10, IFNA16, IL1A, IL1B, IL33*
Pathogenesis of Multiple Sclerosis	1.34	na	*CCL5, CCR5, CXCL10*
Prostanoid Biosynthesis	1.34	na	*PTGDS, PTGES, PTGES2*
UDP-N-acetyl-D-galactosamine Biosynthesis II	1.34	na	*GCK, HK1, UAP1*
Choline Biosynthesis III	1.34	-2.000	*HMOX1, PCYT1A, PLD2, PLD3*
Thyroid Cancer Signaling	1.33	na	*CTNNB1, CXCL12, MYC, NGF, NTRK1, RAP2A, RRAS, RXRB, TP53*
Melatonin Degradation II	1.33	na	*MAOA, MAOB*
Fatty Acid β-oxidation III (Unsaturated, Odd Number)	1.33	na	*ECI1, EHHADH*
MSP-RON Signaling Pathway	1.33	na	*ACTA2, ACTB, ACTC1, ACTG1, ACTG2, CCL2, KLK1, KLK4, KLKB1, MST1*
Xenobiotic Metabolism Signaling	1.32	na	*ALDH16A1, ALDH18A1, ALDH1L1, ALDH5A1, ALDH9A1, ARNT, CES1, CES2, FMO4, FMO5, GCLC, GSTA1, GSTA3, GSTM1, GSTM4, GSTO1, GSTP1, HMOX1, HS3ST5,*

			HS6ST1, HS6ST2, IL1A, IL1B, MAOA, MAOB, MAP3K15, MAP3K2, MAP3K5, NFE2L2, NQO2, PPP2R5A, PRKCB, RAP2A, RRAS, SOD3, SRA1
Phagosome Formation	1.32	na	*FCER1G, FNBP1, ITGA2, ITGA5, MARCKS, MRC2, MSR1, PLCB4, PRKCB, RAC2, RHOB, RHOQ, RND2, RND3, SCARA3, SYK, TLR3, TLR6*
STAT3 Pathway	1.31	-3.357	*IL10RB, IL11RA, IL15RA, IL17RA, IL1A, IL1B, IL27RA, IL2RG, IL4R, IL6R, MAP3K20, MAPK10, MYC, NTRK1, PDGFRA, RAP2A, RRAS, TGFB1, VEGFA*

Supplemental Table 9. Canonical pathways overrepresented in bacteria oocytes comparing day 4 and day 60 collection.

**Canonical Pathways**	**-log (*P*-value)**	**z-score**	**Molecules**
Crosstalk between Dendritic Cells and Natural Killer Cells	3.23	na	*ACTA1, HLA-DRA, IL2RG, TYROBP*
GADD45 Signaling	2.53	na	*BRCA1, CCNE1*
DNA damage-induced 14-3-3σ Signaling	2.53	na	*BRCA1, CCNE1*
Phagosome Maturation	2.4	na	*CTSF, CTSK, HLA-DRA, TUBA8*
4-hydroxybenzoate Biosynthesis	2.37	na	*TAT*
4-hydroxyphenylpyruvate Biosynthesis	2.37	na	*TAT*
TGF-β Signaling	2.09	na	*AMHR2, INHBB, SMAD9*
IL-9 Signaling	2.03	na	*IL2RG, IL9*
Clathrin-mediated Endocytosis Signaling	2.01	na	*ACTA1, AP1S2, PON1, VEGFD*
5-aminoimidazole Ribonucleotide Biosynthesis I	1.89	na	*PPAT*
S-adenosyl-L-methionine Biosynthesis	1.89	na	*MAT1A*
LXR/RXR Activation	1.82	na	*LBP, PON1, SAA1*
FXR/RXR Activation	1.77	na	*PON1, SAA1, SDC1*
Melatonin Degradation II	1.77	na	*MAOA*
Tyrosine Degradation I	1.67	na	*TAT*
Th2 Pathway	1.67	na	*HLA-DRA, IL2RG, IL9*
Autophagy	1.55	na	*CTSF, CTSK*
Superpathway of Melatonin Degradation	1.50	na	*CYP4B1, MAOA*
Remodeling of Epithelial Adherens Junctions	1.45	na	*ACTA1, TUBA8*
Role of JAK1 and JAK3 in γc Cytokine Signaling	1.44	na	*IL2RG, IL9*
Th1 and Th2 Activation Pathway	1.42	na	*HLA-DRA, IL2RG, IL9*
Caveolar-mediated Endocytosis Signaling	1.41	na	*ACTA1, CD48*

T Helper Cell Differentiation	1.41	na	*HLA-DRA, IL2RG*
Calcium Transport I	1.38	na	*ATP2B2*
Toll-like Receptor Signaling	1.37	na	*LBP, TNFAIP3*
Dopamine Receptor Signaling	1.36	na	*MAOA, NCS1*
Dendritic Cell Maturation	1.35	na	*CD1D, HLA-DRA, TYROBP*
Hepatic Fibrosis / Hepatic Stellate Cell Activation	1.34	na	*COL27A1, LBP, VEGFD*
Purine Nucleotides De Novo Biosynthesis II	1.34	na	*PPAT*
Glycogen Degradation II	1.30	na	*PYGM*
Guanosine Nucleotides Degradation III	1.30	na	*XDH*

Supplemental Table 10. Gene networks enriched among differentially expressed genes of oocytes collected from bacteria infused heifers at day 4.

Gene network*	Score†	Molecules in network‡
Cell Morphology, Cell-To-Cell Signaling and Interaction, Connective Tissue Development and Function	36	AIF1L, Ap1, Ap2, C1QC, CD1D, CD37, CSRP3, CTSS, cytochrome-c oxidase, Cytokeratin, Ecm, GAS1, GLRX2, hemoglobin, HOXA2, IFITM1, IgG, IgG1, IgG2a, Igg3, Keratin, KMT2E, KRT1, KRT18, KRT72, KRT8, LUM, MAF, MFAP4, MHC II, Raf, SLC39A8, TAGLN, TGFB1, TYROBP
Antimicrobial Response, Connective Tissue Development and Function, Inflammatory Response	34	AChR, BMP, BMP2, BMPR1B, CDC42EP1, CHRNA5, COL1A2, ID1, IFN alpha receptor, IFN alpha/beta, IFN type 1, Ifnar, IFNAR2, IRF2, ISG15, JAK, LRCH3, LTC4S, MARCH3, MEFV, NFkB (complex), nicotinic acetylcholine receptor, NRG (family), OAS1, P3H2, Pdi, RSAD2, Smad, Smad1/5/8, Smad2/3-Smad4, SMAD9, TCIM, TG, TREX1, USP18
Cellular Development, Cellular Movement, Neurological Disease	32	AHSG, Akt, AKT1S1, AMIGO2, ANKRD1, ARHGAP35, c-Src, C8B, cAMP-Gef, DLC1, DNER, EGLN, Fc gamma receptor, GPNMB, growth factor, MAC, MTORC2, NADPH oxidase, NDNF, NFkB (family), Notch, NTS, RhoGap, RND3, RNF125, Serine Protease, SIRPA, Sphk, ST8SIA2, ST8SIA4, STX17, TLR6, TNFAIP6, TSH, YOD1
Cellular Movement, Hematological System Development and Function, Immune Cell Trafficking	28	Adaptor protein 2, ANXA2, APLF, C5AR1, CCL2, CCR5, CERS5, CHD1, chemokine, Ck2, G protein, G protein alphai, GNRH, Gpcr, GPRC5B, Histone h3, Ikb, IKK (complex), INO80B, MET, Mmp, NDP, PALMD, PCDHGC3, PLC, POLR2K, RAC2, Ras homolog, RNA polymerase II, Rnr, S100A4, Secretase gamma, SPTA1, Vegf, WDR75
Developmental Disorder, Hereditary Disorder, Neurological Disease	26	Alp, AMOTL2, Angiotensin II receptor type 1, CCN1, CCN2, CD68, COL1A1, COL6A6, collagen, Collagen type I, Collagen type III, Collagen type IV, ERK, Fcer1, GPIIB-IIIA, IFI44L, Igfbp, JINK1/2, Laminin1, LDL, LRP, MSLN, NFAT (complex), NID1, OLR1, PDGF BB, PLD2, POSTN, Rock,

		SERPINE1, SIM2, SNAI2, Tgf beta, TMEM204, VCAN
Gene Expression, Neurological Disease, Organismal Injury and Abnormalities	26	ANAPC10, APC (complex), ATF3, C/EBP, caspase, CCNE1, Cdc2, CEBPD, CITED2, Cyclin A, Cyclin D, Cyclin E, E2f, ELF4, FOSB, Gcn5l, histone deacetylase, Jnk, JUNB, LFNG, MAP1LC3, PIK3R3, PNPO, Rb, Smad2/3, SRA1, TCF, TH2 Cytokine, TIMP3, Tnf receptor, TNFRSF12A, TNFRSF1B, TP63, transglutaminase, TRPV2
Cardiovascular Disease, Ophthalmic Disease, Organismal Injury and Abnormalities	24	Cathepsin, CLDN5, Collagen Alpha1, Collagen type II, Collagen(s), CSPG4, CTSH, elastase, ERK1/2, ETS, Fibrin, gelatinase, GMFG, Hspg, IGFBP5, Kallikrein, Laminin (complex), MMP19, Pdgf Ab, plasminogen activator, PLAT, PLG, RAD1, RGCC, SDC1, SERPINC1, SLC12A6, SMAD1/5, SMOOTH MUSCLE ACTIN, SULF2, THBS2, Thrombospondin, TLR7/8, TMEM176B, trypsin
Hereditary Disorder, Immunological Disease, Organismal Injury and Abnormalities	24	C1QL3, CD14, CXCL16, FCGR1A, FSHB, HDL, HERC6, HSPB6, Ifi27, IFIT1, IFIT5, Ifn, IFN Beta, Ifn gamma, Iga, IL12 (complex), IL12 (family), IL23, IL9, Interferon alpha, IRF, JAK1/2, LAPTM5, LITAF, MHC Class II (complex), MX1, PI3K (complex), PI3K (family), PI3K p85, Pro-inflammatory Cytokine, STAT5a/b, STC1, Tlr, Tnf (family), TNFAIP3
Cell Morphology, Cellular Assembly and Organization, Cellular Compromise	24	Actin, Alpha catenin, Alpha tubulin, BCR (complex), CAVIN1, CD3, CD9, CTHRC1, Fgf, FGF20, FHL3, Fibrinogen, Focal adhesion kinase, FOXN4, GSN, IBTK, Ige, Integrin, MAOA, Mapk, NBEAL1, NEDD9, P glycoprotein, p85 (pik3r), PARP10, Pld, PTK, Rac, SDC2, Shc, Sos, SRC (family), STEAP2, TAGLN2, TUFT1
Gastrointestinal Disease, Inflammatory Disease, Ophthalmic Disease	24	AP4E1, APBB2, C2orf68, CAMKMT, CEMIP, DUSP18, EGFR, ELAVL1, GLIPR2, HNRNPDL, HSP90AA1, HYAL, HYAL2, HYAL4, hyaluronic acid, hyaluronidase, ITIH2, KATNBL1, MAP3K15, MAP4K4, MKX, NT5M, NUTF2, PCMTD1, PI4K2B, QTRT1, RIN2, SLC45A2, SRRM4, ST3GAL2, STK35, STK36, TNFAIP6, TNNI3K, Tpsab1

Lipid Metabolism, Molecular Transport, Small Molecule Biochemistry	20	AIFM2, Alpha actin, ANGPTL4, APOE, Cbp/p300, CFAP44, CLDN23, ENaC, estrogen receptor, Growth hormone, Hdac, HDL-cholesterol, HISTONE, Histone h4, Hsp70, Hsp90, Insulin, JMY, KLF7, LDL-cholesterol, MYEF2, N-cor, Nr1h, NRG1, P38 MAPK, p70 S6k, PCK2, Proinsulin, Rxr, SFXN3, TSSK6, UBA2, Ubiquitin, VLDL-cholesterol, ZBTB7C
Cardiovascular System Development and Function, Organismal Development, Tissue Morphology	20	ADK, AGXT2, Ang4, ANKH, ARHGEF4, ASH1L, beta-estradiol, CAP2, CRADD, CTSH, CXXC5, DHRS7, ERMARD, FAM111A, FAM222A, FBXO16, HGF, HNRNPL, LHX9, MAB21L1, MPEG1, N, N-dimethylarginine, nitric oxide, PBX1, phosphate, RLN2, SRPX2, STC1, STK10, TMIE, TNFAIP6, TREML2, ZFP30, ZFP36L2, ZSWIM6
Cancer, Cellular Development, Organismal Injury and Abnormalities	20	ABHD1, ANAPC10, CBL, CCDC155, CCL15, CDKN1A, CIB3, DBH-AS1, Dgk, DMTN, DNASE1L3, DOK5, DSE, ESR2, F2, GLIPR2, H2-T24, HMGXB3, IL19, KIAA1324, KLF17, MAPK1, MTIF3, NEBL, NTN4, PAICS, PEBP4, putrescine, RAB39B, RECQL4, SOCS1, TCN2, TIGD3, TTC6, ZNF205
Cellular Compromise, Organismal Injury and Abnormalities, Skeletal and Muscular Disorders	18	ANKS4B, APP, ARFGAP3, ARIH1, BEND5, C1QC, CA14, CCDC63, DNAH1, DNAH10, DNAH14, DNAH5, DNALI1, FANCD2, FN1, HIST1H2BH, HSPB6, KRT81, LOXL3, MEIOC, MLXIP, MTFMT, MYL1, NAALADL2, phospholipid, PLPP4, RAB38, RAB43, SNX24, SON, SPATA46, SPEG, XIRP2, YAP/TAZ, YWHAG
Cardiac Enlargement, Cardiovascular Disease, Cell-To-Cell Signaling and Interaction	16	15-epi-lipoxin A4, 7S NGF, 8-hydroxyeicosapentaenoic acid, arachidic acid, B4GALT6, BSCL2, Cutal, EXOC3L4, GALNT14, GPR108, GTPase, IL17D, IL6, L-lactic acid, LINC00963, MRAP, Msantd2, MYO1G, NfkB-RelA, norepinephrine, NT5C, PDE4DIP, RNASE1, RNASE6, RSPO3, SFRP5, SMLR1, SNCA, SPON2, SRGAP2, TNF, TNIK, UCN2, UCP1, USP17L2 (includes others)

Amino Acid Metabolism, Cell-To-Cell Signaling and Interaction, Lipid Metabolism	16	7S NGF, ADGRB3, AGTR1, BARX2, Ca2 , CARF, COL16A1, CYTH4, DBNDD2, DDIT4L, DNASE1, DNM3, DYNLL1, ESR1, FOS, GAL3ST4, GPR12, GPR19, GPR85, HIF1A, HIST1H2AM, L-lactic acid, MFAP4, MSLN, NTSR2, PEBP4, PIGF, PKIB, PKIG, SEC22C, SHISA5, SLC24A4, SNAPC5, SPAG4, TP53
Cell-To-Cell Signaling and Interaction, Cellular Function and Maintenance, Molecular Transport	12	26s Proteasome, ACTA2, Adducin, CDC42SE1, cytokine, FSH, GNPDA1, HERC4, HNRNPM, Igg2, Igm, IL1, Immunoglobulin, Metalloprotease, MOV10, MTORC1, Pdgfr, Pka, PKAr, POLR2K, PPP1R26, PRKAR1B, PRLH, protein phosphatase, RAD52, RAS, RNF128, SNPH, STAT, STX1B, Syt7, TCR, TNFAIP6, vitamin K1, ZNFX1
Cancer, Gastrointestinal Disease, Hepatic System Disease	8	5-oxo-6-8-11-14-(e, z, z, z)-eicosatetraenoic acid, ABCC1, acetaldehyde, Adaptor protein 1, CALHM2, CAMK2N1, CGB3 (includes others), CPXM2, CSPG4, DCT, ERF, GBX2, GOT1, IGK, KIF5C, LOC102724788/PRODH, MAPK3, MAPKAPK5, MAZ, METAP2, mevalonic acid, miR-24-3p (and other miRNAs w/seed GGCUCAG), MYC, NfkB1-RelA, NSMF, OTX2, PFKFB1, PLXNA4, PROKR1, prostaglandin E1, SEMA6D, THBS2, TLE4, ULBP3, ZXDB
Auditory Disease, Hereditary Disorder, Metabolic Disease	5	14-3-3, ADRB, AMPK, Calcineurin A, Calcineurin protein(s), calpain, CaMKII, CG, Cofilin, COQ6, Creb, cytochrome C, EGR1, F Actin, Gsk3, Hsp27, L-type Calcium Channel, Lh, MAP2, MAP2K1/2, Mek, Mlc, MLYCD, Myosin, Nfat (family), Ngf, NMDA Receptor, Nos, PARP, Pdgf (complex), Pdlim3, PEPCK, Pkc(s), PP2A, Sod
Cardiovascular System Development and Function, Embryonic Development, Nervous System Development and Function	2	C6orf15, OGA
Cancer, Cellular Development, Cellular Growth and Proliferation	2	C14orf39, STAT5B

Lipid Metabolism, Small Molecule Biochemistry	2	SMPD5, sphingomyelin phosphodiesterase
Developmental Disorder, Hereditary Disorder, Ophthalmic Disease	1	beta-carotene, LHX1, SLC16A12, TET2

*Enriched gene networks determined by Ingenuity Pathway Analysis using significantly differentially expressed genes only. †Network score is derived from a P value and indicates the likelihood of the genes in a network being found together due to random chance. A network score of 2 or greater gives a 99% confidence the network and genes not being generated by random chance alone. ‡All genes in a given network, capitalized genes are differentially regulated.

Supplemental Table 11. Gene networks enriched among differentially expressed genes of oocytes collected from bacteria infused heifers at day 60.

**Gene network***	**Score†**	**Molecules in network‡**
Dental Disease, Developmental Disorder, Gastrointestinal Disease	42	ABRA, Alpha tubulin, BETA TUBULIN, Calcineurin protein(s), CCT6A, CG, CSRP3, EID2, EMX2, ENPP3, F11, FOXL2, FSH, GPC1, Hdac, HSD3B2, IPO8, KIF21A, KLK4, KRT8, L-type Calcium Channel, LGALS3BP, Lh, MAGED2, MYOF, ODAPH, OXT, Pkc(s), QRSL1, RTN4, Serine Protease, ST14, TUBA1A, ZBTB38, ZNF484
Cell Morphology, Cellular Function and Maintenance, Reproductive System Development and Function	40	Akt, ARHGAP6, ASB9, C1q, C1S, CDH13, CHRNA10, CHRNA9, Complement, creatine kinase, DHRS7, DIPK2A, DNER, Foxp1, HOPX, Igm, Kallikrein, LAPTM4B, MLC1, MYH3, Ngf, nicotinic acetylcholine receptor, NRG (family), PCDHGC3, PROS1, PTX3, RhoGap, RHOQ, RRAGB, Secretase gamma, SERPING1, SERPINH1, SLC38A2, TNFRSF10A, UPK1B
Cell Morphology, Cellular Development, Cellular Movement	35	BCAM, CD151, COL1A1, collagen, Collagen(s), Complement component 1, cytokine receptor, DEPDC1, ERK1/2, FBN2, Fcer1, IL27RA, INSL3, Integrin, Integrin alpha 4 beta 1, ITGA2, JAM2, JINK1/2, Laminin (complex), MAP2K1/2, MATN2, MFGE8, MKX, PEPD, PLEK, PLPP3, POSTN, Rap1, RASGRP3, SH3BGRL, TNS1, VEGFD, Vla-4, VNN1, WDR75
Cell Signaling, Dermatological Diseases and Conditions, Immunological Disease	35	ARHGAP45, CD99, G0S2, GLMP, HIVEP2, HSD17B7, Ifi27, IFI6, Ifn, IFN Beta, IFN type 1, Ifnar, IFNW1, IL-2R, IL2RG, Interferon alpha, IRF9, ISG15, ISGF3, JAK, JAK1/2, MHC Class I (complex), MHC CLASS I (family), MX1, NFkB (complex), Nuclear factor 1, OAS1, RNASEL, RSAD2, TCIM, TNFSF18, TRANK1, UBA7, USP18, VSNL1
Cell-To-Cell Signaling and Interaction, Connective Tissue Development and Function, Skeletal and Muscular System Development and Function	33	Alpha catenin, Cbp/p300, CCNT2, Collagen type I, FADS2, FAM20C, FRK, H2AF, H2AFY2, H2AFZ, histone deacetylase, Histone H1, Histone h4, HMGN5, Holo RNA polymerase II, HPSE, IFIT5, Jnk, N-cor, NMI, PARP10, PLOD2, Rar, SDC4, SPARCL1, STAT5a/b, STRA6, TDG, Tgf

		beta, TGM2, THBS2, thyroid hormone receptor, TNFAIP6, TTC27, ZNF189
Lipid Metabolism, Small Molecule Biochemistry, Vitamin and Mineral Metabolism	29	ALT, APOE, C/EBP, CPT1, CYP19A1, CYP27A1, EHHADH, ERK, Gap, GPIHBP1, Growth hormone, HDL, HDL-cholesterol, hemoglobin, KLF10, LDL, LDL-cholesterol, LDLR, LPL, MBTPS2, Nr1h, PCYT2, PDK4, PON1, PTGES, RASA2, RCVRN, SAA, SAA1, SCP2, SPSB1, Srebp, THEMIS2, TMSB4, VLDL-cholesterol
Cell Cycle, Cellular Development, Cellular Growth and Proliferation	29	26s Proteasome, ABCB6, ACADM, CCNC, chymotrypsin, cytochrome-c oxidase, DDAH2, EGR1, FOS, GTPBP1, HISTONE, Histone h3, INTERLEUKIN, MAN1A2, MAOA, mediator, Mitochondrial complex 1, Nos, P glycoprotein, PEPCK, Pkg, PPOX, PQBP1, PSMA2, RAB38, RGS3, RNA polymerase II, SLC34A1, SLC4A10, SMOC2, SNAI2, ST8SIA4, TCF, trypsin, Ubiquitin
Lipid Metabolism, Molecular Transport, Small Molecule Biochemistry	29	ANKRD55, BCDIN3D, C1QTNF1, CALHM4, CCDC80, CUL3, DCUN1D4, ELAVL1, FUCA2, FUT4, GLOD4, GPRC5B, IFI6, IFT74, LMNA, LTA4H, MARVELD1, MCC, MOB3B, MSANTD3, OIP5-AS1, PTPN9, PUS7, PXDC1, RNF149, SH3D19, SLC27A5, SYMPK, TCIM, TM4SF20, TMEM14A, TNF, UAP1, ZMAT4, ZNF395
Gastrointestinal Disease, Inflammatory Disease, Ophthalmic Disease	25	ABHD14B, ADAMTS9, adenosine triphosphate, AGPAT1, CIAO1, CPB2, DPAGT1, FUT10, FUT11, GPR83, HERC6, HNF4A, HOXC8, HYAL, HYAL2, HYAL4, hyaluronic acid, hyaluronidase, IFNG, IL6, ITIH3, KCNMB4, MTNR1A, MX, PADI2, SBNO2, SLC22A3, SMIM3, SSTR4, TCIM, TNFAIP6, TNFSF18, TTC25, WBP1, ZFP37
Cell Death and Survival, Cellular Assembly and Organization, Cellular Function and Maintenance	23	ACTR5, ADCY, ADRB, CACNA2D3, Creb, EPHX2, G protein alpha, GNRH, GOT, Gpcr, GPR50, GPRC5B, IgG, IgG1, Igg3, Immunoglobulin, MCRS1, NIPSNAP3A, OXTR, P38 MAPK, p85 (pik3r), PHF20L1, Pka, Rac, Ras homolog, Rgs, RGS17, RGS6, RHO, S100A11, SERTAD3, SRC (family), TNFRSF1A, USHBP1, voltage-gated calcium channel

Carbohydrate Metabolism, Protein Degradation, Small Molecule Biochemistry	21	ADGRE2, ANKRD33B, ASB9, ASPH, BACE2, beta-estradiol, Ca2 , Cabp1, CASQ, CHML, DCAF6, DPP7, FZD10, GIPC2, GPRC6A, GRM3, GTPase, HSPD1, KCTD7, KHDC4, LHFPL2, MICOS10-NBL1/NBL1, MYOF, NBPF10 (includes others), NPS, Rcan1, SNN, SNX12, SPOCK2, SSTR4, STK38L, TRIM25, XCR1, ZDHHC14, ZNF567
Carbohydrate Metabolism, Nervous System Development and Function, Tissue Morphology	19	AKAP4, CARD6, CD58, CDAN1, CKMT1A/CKMT1B, EGFR, EMP1, ETF1, ethanol, Folh1, FUT4, GABRD, Gpm6b, GPRC5B, HOXB1, IDH3G, IL2, KLHL11, LRRC75A, mir-296, MTA2, NAP1L5, PHLDA2, PIP4P2, POU5F1, PRKAR2B, PSEN1, Sectm1b, SYT2, TNFAIP6, TNFRSF21, TNXB, TRIM35, UBC, WARS
Carbohydrate Metabolism, Cellular Development, Cellular Growth and Proliferation	19	ADGRG5, ADORA3, ALG5, APLN, BCAM, Endothelin, FGF11, FZD3, Gli-Kif7-Stk36-Sufu, GPR161, GPR50, GPRC5B, HIF1A, IDH3A, IDH3B, LPAR1, LZTS3, MAS1, MPP5, Msantd2, PALM2-AKAP2, PLSCR4, POMK, PRKACA, PRKAR1B, SLC19A2, SMO, SSTR3, STK36, TMEM11, TMEM120B, TMEM246, TMEM45A, TNIK, ZDHHC9
Cell Morphology, Cellular Assembly and Organization, DNA Replication, Recombination, and Repair	19	BPTF, BRCA1, C22orf31, CASP8AP2, CCDC71, CFAP47, CHD1, ELMO3, HDAC1, HDAC8, HEXD, HIST3H3, HOPX, HSPA8, IFI6, IMPG1, JPH2, MKX, NDST1, NEPRO, NPM1, OARD1, PALMD, RPP25, RPP40, SEC23IP, SMCHD1, SOX30, TEX45, TYW3, YTHDF3, ZBTB47, ZKSCAN4, ZNF395, ZNF496
Connective Tissue Disorders, Dermatological Diseases and Conditions, RNA Post-Transcriptional Modification	17	Actin, AIFM2, C1QTNF1, C1QTNF4, Ck2, CTHRC1, DHX9, dihydroxyacetone, EBAG9, EGLN, FKBP51-TEBP-GR-HSP90-HSP70, Focal adhesion kinase, GALNS, HLA-B, Hsp70, Insulin, LGALS12, LIG1, NLRP10, NR3C1, Pdgfr, PHGDH, PLC, Proinsulin, RARRES2, RAS, Rasgrp, Shc, SIAE, SRSF6, STAT, TCR, TRA2B, Vegf, WDR31
Cancer, Cell Death and Survival, Post-Translational Modification	17	ADAM22, ANAPC5, CA10, CKAP2, DLG1, DNAJC12, DNAJC15, FBXO15, FFAR3, Foxp2, FZD6, GDA, GLYCTK, GPR17,

		GPR179, GPR83, GPRC5B, KCTD15, LGI1, LY6G5B, MNS1, MSRB1, MSRB3, MYCN, PCLAF, PTGER1, RAB28, SUCLG2, TCEAL4, TM6SF1, TMEM178A, TP53, TRIM27, UBE2N, UBL5
Cardiac Dilation, Cardiac Enlargement, RNA Post-Transcriptional Modification	15	ABCF2, ATP5MF-PTCD1, BMS1, C18orf21, CHD9, CWC15, DDX42, ESF1, ESR2, FAM210A, FKTN, HIST1H2BK, ISG20L2, LMAN2, LSG1, MICALL2, MROH8, NXF1, PCYOX1L, PNISR, PRCC, QPRT, R3HCC1, RBM11, RBM15B, RBM45, Rnr, RPP14, RPP40, SAP30BP, SF3A2, SLC4A1AP, TKTL2, WDR12, ZNF740
Cardiovascular Disease, Cardiovascular System Development and Function, Organ Morphology	13	AMPK, Ap2, BCR (complex), BTC, caspase, CCND2, Cyclin A, Cyclin D, Cyclin E, cytochrome C, DCHS1, ERBB3, estrogen receptor, GBA, Gsk3, HOXC10, Hsp27, Hsp90, Ifn gamma, Mek, Nfat (family), p70 S6k, PARP, PARP3, PDGF BB, PELP1, PI3K (complex), PI3K (family), PI3K p85, PTK, PTPRC, Raf, RND3, Sos, TSC22D3
Cellular Compromise, Neurological Disease, Organismal Injury and Abnormalities	13	Alp, Ap1, CD3, CD58, chemokine, CLU, Cofilin, Collagen type IV, cytokine, F Actin, HIST1H2AC, Iga, Ige, IL1, IL12 (complex), IL12 (family), IL33, KLHL13, KLHL28, LGALS9, Mapk, Metalloprotease, MHC Class II (complex), Mmp, NFAT (complex), Pdgf (complex), Pka catalytic subunit, PNP, PPP1R3B, Pro-inflammatory Cytokine, Rock, Tlr, Tnf (family), TPBG, XCL1
Cellular Development, Reproductive System Development and Function, Tissue Development	2	HHIPL1, ING4
Behavior, Cancer, Endocrine System Disorders	2	GLP1R, SYS1
Cell Morphology, Cellular Assembly and Organization, Cellular Compromise	2	C12orf73, GHITM
Cancer, Hereditary Disorder, Organismal Injury and Abnormalities	2	HOXB13, POPDC3, SCRN1
Cell Cycle, Cell Morphology, Cellular Assembly and Organization	2	MEI4, PAX7, REC114

Developmental Disorder, Hereditary Disorder, Immunological Disease	2	ARMCX4, DPAGT1, PDE4DIP

*Enriched gene networks determined by Ingenuity Pathway Analysis using significantly differentially expressed genes only. †Network score is derived from a P value and indicates the likelihood of the genes in a network being found together due to random chance. A network score of 2 or greater gives a 99% confidence the network and genes not being generated by random chance alone. ‡All genes in a given network, capitalized genes are differentially regulated.

Supplemental Table 12. Gene networks enriched among differentially expressed genes of control oocytes comparing day 4 and day 60.

**Gene network***	**Score†**
Connective Tissue Disorders, Developmental Disorder, Hereditary Disorder	38
Cancer, Protein Synthesis, RNA Damage and Repair	36
Amino Acid Metabolism, Post-Translational Modification, Small Molecule Biochemistry	36
Developmental Disorder, Ophthalmic Disease, Organismal Injury and Abnormalities	34
Molecular Transport, RNA Post-Transcriptional Modification, RNA Trafficking	34
Amino Acid Metabolism, Neurological Disease, Small Molecule Biochemistry	32
Cell Death and Survival, Developmental Disorder, Gastrointestinal Disease	32
Auditory Disease, Dermatological Diseases and Conditions, Developmental Disorder	32
Nervous System Development and Function, Organ Morphology, Tissue Morphology	30
Cardiovascular Disease, Heart Failure, Organismal Injury and Abnormalities	30
Carbohydrate Metabolism, Developmental Disorder, Small Molecule Biochemistry	30
Cellular Assembly and Organization, Cellular Movement, Reproductive System Development and Function	30
Cell Morphology, Connective Tissue Disorders, Dermatological Diseases and Conditions	30
Cell Morphology, Cellular Assembly and Organization, Cellular Function and Maintenance	30
Carbohydrate Metabolism, Infectious Diseases, Lipid Metabolism	28
Cell Morphology, Nucleic Acid Metabolism, Small Molecule Biochemistry	28
Cell Cycle, Cellular Assembly and Organization, Cellular Function and Maintenance	28
Cancer, Protein Synthesis, RNA Damage and Repair	28
Developmental Disorder, Hereditary Disorder, Neurological Disease	28
Developmental Disorder, Hereditary Disorder, Metabolic Disease	28
Cellular Development, Cellular Growth and Proliferation, Organismal Injury and Abnormalities	28
Carbohydrate Metabolism, Post-Translational Modification, Small Molecule Biochemistry	26
Hereditary Disorder, Nephrosis, Organismal Injury and Abnormalities	26
Cancer, Connective Tissue Disorders, Tissue Development	26
Cell Signaling, Cellular Growth and Proliferation, RNA Post-Transcriptional Modification	26

*Enriched gene networks determined by Ingenuity Pathway Analysis using significantly differentially expressed genes only. †Network score is derived from a P value and indicates the likelihood of the genes in a network being found together due to random chance. A network

score of 2 or greater gives a 99% confidence the network and genes not being generated by random chance alone.

Supplemental Table 13. Gene networks enriched among differentially expressed genes of oocytes from bacteria infused heifers comparing day 4 and day 60.

**Gene network***	**Score†**
Connective Tissue Development and Function, Inflammatory Response, Organismal Injury and Abnormalities	47
Connective Tissue Development and Function, Connective Tissue Disorders, Organismal Injury and Abnormalities	36
Embryonic Development, Organ Development, Organismal Development	24
Cancer, Cell-To-Cell Signaling and Interaction, Cellular Development	24
Developmental Disorder, Hair and Skin Development and Function, Hereditary Disorder	21
Cell Morphology, Cellular Assembly and Organization, Drug Metabolism	17
Cell Death and Survival, Cellular Function and Maintenance, Hematological System Development and Function	13
Cell Cycle, Cell Morphology, DNA Replication, Recombination, and Repair	2
Cellular Assembly and Organization, Metabolic Disease, Organismal Injury and Abnormalities	2

*Enriched gene networks determined by Ingenuity Pathway Analysis using significantly differentially expressed genes only. †Network score is derived from a P value and indicates the likelihood of the genes in a network being found together due to random chance. A network score of 2 or greater gives a 99% confidence the network and genes not being generated by random chance alone.

Supplemental Table 14. Predicted upstream regulators of differentially expressed genes in oocytes from bacteria infused heifers at day 4.

**Upstream regulator**	**Predicted activation state**	**Activation z-score**	***P*-value of overlap**	**Target molecules in dataset**
Lipopolysaccharide	Inhibited	-4.993	< 0.0001	*ACTA2, ANGPTL4, APOE, ATF3, BMP2, C5AR1, CAVIN1, CCL2, CCN1, CCN2, CCNE1, CCR5, CD14, CD1D, CD37, CD68, CD9, CEBPD, CITED2, CLDN5, COL1A1, COL1A2, CSPG4, CXCL16, EGR1, FOSB, GAS1, GMFG, GSN, IFI44L, IFIT1, IFIT5, IFITM1, IGFBP5, IL9, IRF2, ISG15, JUNB, LITAF, LTC4S, MAF, MAOA, MEFV, MET, MX1, NID1, NTS, OAS1, OLR1, PARP10, PLAT, PLG, RGCC, RND3, RSAD2, SDC1, SERPINC1, SERPINE1, SIRPA, SLC39A8, TCIM, TGFB1, THBS2, TIMP3, TLR6, TNFAIP3, TNFAIP6, TNFRSF1B, TP63, TREX1, TYROBP, USP18, VCAN*
TGFB1	Inhibited	-4.692	< 0.0001	*ACTA2, AIF1L, AMOTL2, ANGPTL4, ANKRD1, ANXA2, APOE, ARHGAP35, BMP2, C1QC, C5AR1, CCL2, CCN1, CCN2, CCNE1, CCR5, CD14, CD68, CITED2, COL1A1, COL1A2, CSPG4, CTSH, CTSS, EGR1, ELF4, FCGR1A, FOSB, GAS1, GLRX2, GPRC5B, GSN, HNRNPDL, HOXA2, ID1, IGFBP5, IL9, JUNB, KMT2E, KRT18, KRT8, LITAF, LTC4S, MAF, MAOA, MEFV, MET, MFAP4, NEDD9, OLR1, PLAT, POSTN, RAD1, RGCC, RSAD2, S100A4, SDC1, SERPINE1, SLC39A8, SNAI2, TAGLN, TG, TGFB1, THBS2,*

				TIMP3, TNFAIP3, TNFAIP6, TNFRSF12A, VCAN
PRL	Inhibited	-3.818	< 0.0001	*ANXA2, CCL2, CEBPD, CLDN5, COL1A1, COL1A2, CTSH, CTSS, EGR1, GPNMB, HERC6, ID1, IFI44L, IFIT1, IFIT5, IFITM1, IGFBP5, ISG15, KRT72, OAS1, PARP10, PLAT, RSAD2, SDC1, TGFB1, TP63, USP18*
STAT3	Inhibited	-2.292	< 0.0001	*ACTA2, AHSG, ANGPTL4, C5AR1, CCL2, CCN2, CCNE1, CCR5, CD9, CEBPD, COL1A1, COL1A2, EGR1, FCGR1A, HERC6, ID1, IFIT1, IFIT5, IFITM1, IGFBP5, IL9, ISG15, JUNB, MAF, MAP2, MX1, OAS1, RSAD2, SERPINE1, SMAD9, SNAI2, TAGLN, TGFB1, TNFRSF1B, USP18, VCAN*
IFNG	Inhibited	-3.683	< 0.0001	*ACTA2, AIF1L, ANGPTL4, ATF3, C1QC, C5AR1, CCL2, CCN2, CCR5, CD14, CD1D, CD68, CEBPD, COL1A1, COL1A2, CTSH, CTSS, CXCL16, EGR1, FCGR1A, FOSB, GPRC5B, HERC6, ID1, IFI44L, IFIT1, IFIT5, IFITM1, IL9, IRF2, ISG15, JUNB, MEFV, MX1, NEDD9, OAS1, RAC2, RGCC, RSAD2, SDC1, SERPINE1, SNAI2, TG, TGFB1, TIMP3, TLR6, TNFAIP6, TNFRSF12A, TNFRSF1B, TP63, TYROBP, UBA2, USP18*
SMAD7	Activated	2.204	< 0.0001	*ACTA2, BMP2, BMPR1B, CCL2, CCN2, CCNE1, CITED2, COL1A1, COL1A2, FSHB, ID1, KRT8, MET, PLAT, SERPINE1, TAGLN, TGFB1, TIMP3*
SNAI1	Inhibited	-2.035	< 0.0001	*ANKRD1, CCL2, CCN1, CCN2, CCNE1, COL1A1, COL1A2, GSN, ID1, KRT18,*

				KRT8, SDC1, SNAI2, TGFB1, TP63
tetradecanoylphorbol acetate	Inhibited	-4.316	< 0.0001	*AMIGO2, ANGPTL4, C5AR1, CCL2, CCN1, CCN2, CCNE1, CD14, CD68, CTSH, EGR1, FCGR1A, FHL3, FOSB, FSHB, ID1, IGFBP5, IL9, INO80B, IRF2, ISG15, JUNB, MAF, MEFV, MMP19, NTS, OAS1, OLR1, PALMD, PCK2, PLAT, SDC1, SERPINE1, SLC39A8, SNAI2, STC1, TCIM, TGFB1, TLR6, TNFAIP3, TNFAIP6, TNFRSF12A, TNFRSF1B, VCAN*
F2	Inhibited	-3.683	< 0.0001	*ACTA2, ANGPTL4, CCL2, CCN1, CCN2, CD68, CDC42EP1, COL1A1, EGR1, FOSB, IGFBP5, JUNB, PLAT, RAC2, SERPINC1, SERPINE1, TAGLN2, TNFAIP3, TNFRSF12A, ZNF205*
VEGFA	Inhibited	-3.934	< 0.0001	*ACTA2, BMP2, CCL2, CCN1, CCN2, CCNE1, CD68, CITED2, COL1A1, CTSS, EGR1, FOSB, ID1, IGFBP5, JUNB, PLAT, SERPINE1, SLC12A6, SNAI2, STC1, TGFB1*
tretinoin	Inhibited	-2.641	< 0.0001	*ANGPTL4, ANXA2, APOE, BMP2, BMPR1B, C5AR1, CCL2, CCN1, CCN2, CCNE1, CCR5, CD14, CD1D, CD68, CD9, CITED2, COL1A1, COL1A2, CTSS, DLC1, EGR1, ELF4, HOXA2, ID1, IFI44L, IFIT1, IFIT5, IFITM1, IGFBP5, IL9, ISG15, KRT1, KRT18, KRT72, LTC4S, MAP2, MMP19, MSLN, OAS1, PLAT, POSTN, SERPINE1, SIRPA, SMAD9, STC1, TGFB1, TNFAIP3, TNFAIP6, TNFRSF1B, TP63, TYROBP, USP18*

IL13	Inhibited	-2.433	< 0.0001	*ATF3, CCL2, CCN2, CCR5, CD14, CD37, COL1A1, COL1A2, CTSH, CTSS, EGR1, FCGR1A, GPNMB, GSN, MAF, MAOA, NID1, POSTN, RIN2, SERPINE1, SNAI2, ST8SIA4, TGFB1, TNFRSF1B*
IL1B	Inhibited	-2.503	< 0.0001	*ACTA2, ANGPTL4, APOE, ATF3, BMP2, CCL2, CCN2, CCR5, CD14, CEBPD, COL1A1, CTSS, EGR1, FOSB, IFIT1, IGFBP5, IL9, ISG15, JUNB, MEFV, MX1, MYEF2, OLR1, PLAT, POSTN, RAC2, RSAD2, SDC1, SERPINE1, TCIM, TGFB1, TIMP3, TMEM176B, TNFAIP3, TNFAIP6, TNFRSF1B, USP18, VCAN*
IL6	Inhibited	-3.089	< 0.0001	*APOE, ATF3, BMP2, C5AR1, CCL2, CCN2, CCNE1, CCR5, CD14, CD68, CEBPD, COL1A1, EGR1, FCGR1A, ID1, IFIT1, IGFBP5, IL9, JUNB, KRT18, KRT8, MAF, MAP2, MET, MFAP4, PLAT, PLG, RNASE6, SERPINE1, SRA1, TGFB1, TLR6, TNFRSF12A, TNFRSF1B*
SMAD3	Inhibited	-3.302	< 0.0001	*ACTA2, ANKRD1, BMP2, CCL2, CCN2, CCNE1, COL1A1, COL1A2, EGR1, FSHB, ID1, IL9, JUNB, LUM, SERPINE1, SNAI2, TAGLN, TGFB1, TIMP3*
bleomycin	Inhibited	-3.615	< 0.0001	*ACTA2, CCL2, CCN2, CCR5, CD68, COL1A1, COL1A2, CTSS, DNER, ID1, PLAT, RGCC, S100A4, SERPINE1, TGFB1, TIMP3, TP63*
poly rI:rC-RNA	Inhibited	-3.701	< 0.0001	*ATF3, BMP2, CCL2, CD14, CEBPD, EGR1, FCGR1A, H2-T24, IFIT1, IFIT5, IRF2, ISG15, JUNB, MX1, OAS1, OLR1, PCK2, PRKAR1B, RSAD2, SNAI2, ST8SIA4, TG,*

				TGFB1, TLR6, TNFAIP3, TNFAIP6, USP18
LDL	Inhibited	-2.657	< 0.0001	*APOE, ATF3, BMP2, CCL2, CCN2, CD68, EGR1, FCGR1A, FOSB, IFITM1, IRF2, MX1, OLR1, PLAT, RND3, SERPINE1, TGFB1, TNFAIP3, TNFAIP6*
TP53	Inhibited	-3.103	< 0.0001	*ACTA2, AIFM2, AMOTL2, ANKRD1, ANXA2, APBB2, APOE, ATF3, C1QC, CCL2, CCN1, CCN2, CCNE1, CEBPD, CITED2, COL1A1, COL1A2, COQ6, CTSH, DDIT4L, DLC1, EGR1, ELF4, GAL3ST4, GAS1, GSN, ID1, IGFBP5, IL9, ISG15, JUNB, KRT18, KRT8, MET, MX1, OAS1, PAICS, PIK3R3, POSTN, RAC2, RND3, S100A4, SERPINC1, SERPINE1, SNAI2, SON, SULF2, TAGLN2, TG, TGFB1, THBS2, TIMP3, TLR6, TNFRSF1B, TP63, VCAN*
PDGF BB	Inhibited	-3.159	< 0.0001	*ACTA2, ATF3, CCL2, CCN1, CCN2, CCNE1, CEBPD, EGR1, FOSB, JUNB, MAP2, OLR1, PLAT, POSTN, RND3, SERPINE1, TAGLN, TGFB1, TNFAIP3, TNFRSF12A, VCAN*
TGFB3	Inhibited	-2.737	< 0.0001	*ACTA2, CCN1, CCN2, COL1A1, COL1A2, FOSB, HOXA2, LTC4S, SERPINE1, SNAI2, TAGLN, TGFB1, TIMP3*
TGFB2	Inhibited	-2.264	< 0.0001	*ACTA2, ANGPTL4, CCN2, COL1A1, COL1A2, EGR1, GAS1, LTC4S, SERPINE1, SNAI2, TAGLN, TGFB1, VCAN*
KRAS	Activated	2.242	< 0.0001	*ATF3, CAVIN1, CCN2, COL1A1, EGR1, FOSB, GAS1, GSN, HNRNPDL, ID1, IFIT1, IFITM1, IRF2, ISG15, JUNB, MET, MSLN, MX1, OAS1,*

				PLAT, S100A4, SIRPA, SNAI2, ST3GAL2, TIMP3, TNFRSF12A, VCAN
decitabine	Inhibited	-2.244	< 0.0001	*ANGPTL4, ANKRD1, ANXA2, APOE, CCN2, CD9, CLDN5, COL1A1, COL1A2, DLC1, DNASE1L3, DNM3, ELF4, GSN, ID1, IFITM1, ISG15, JUNB, KIF5C, KRT18, KRT8, LUM, MAF, MAP2, MET, MX1, MYEF2, OAS1, SDC2, STEAP2, TIMP3, TP63, VCAN*
IL4	Inhibited	-2.357	< 0.0001	*ACTA2, ANXA2, APOE, C5AR1, CCL2, CCN2, CCNE1, CCR5, CD14, CITED2, CLDN5, COL1A1, COL1A2, CXCL16, FOSB, IFNAR2, IL9, ISG15, JUNB, KRT1, LFNG, LTC4S, MAF, MAOA, MEFV, MOV10, MPEG1, NEDD9, PKIB, POSTN, SERPINE1, SIRPA, SON, TGFB1, TIMP3, TMEM176B, TRPV2*
EGF	Inhibited	-2.791	< 0.0001	*ATF3, CCN1, CCN2, CCNE1, CDC42EP1, CEBPD, COL1A1, COL1A2, EGR1, FOSB, GAS1, ID1, IGFBP5, JUNB, KRT72, MET, PLAT, S100A4, SDC1, SERPINE1, SNAI2, TGFB1, TIMP3, TNFRSF12A, TP63, VCAN*
TLR3	Inhibited	-2.965	< 0.0001	*ATF3, CCL2, CSRP3, ID1, IFI44L, IFIT1, ISG15, JUNB, MX1, NTS, OAS1, PIK3R3, RSAD2, SERPINE1, TNFRSF1B, TP63, USP18, ZNFX1*
C5	Inhibited	-2.215	< 0.0001	*ACTA2, ATF3, C5AR1, CCL2, CCNE1, EGR1, FCGR1A, IL9, MET, PLAT, SERPINE1, TGFB1, TNFAIP3*
IFNL1	Inhibited	-3.275	< 0.0001	*ATF3, HERC6, IFI44L, IFIT1, IFIT5, IFITM1, ISG15, MX1, OAS1, RSAD2, USP18*
SMAD4	Inhibited	-2.374	< 0.0001	*ANGPTL4, BMP2, CCL2, CCN2, CCNE1, CITED2,*

				COL1A2, FSHB, ID1, IL9, MET, RGCC, SERPINE1, SNAI2, TGFB1, TIMP3, TNFAIP6
FGF2	Inhibited	-3.212	< 0.0001	*ACTA2, ANGPTL4, CCL2, CCN1, CCNE1, COL1A1, COL1A2, EGR1, GPNMB, IGFBP5, JUNB, MET, PLAT, S100A4, SDC1, SDC2, SERPINE1, SNAI2, TAGLN, TGFB1, TIMP3, TNFRSF12A*
mifepristone	Activated	2.144	< 0.0001	*APOE, ATF3, CCL2, CCN2, CD14, CEBPD, DLC1, EGR1, JUNB, NDP, PLAT, POSTN, SERPINE1, TGFB1, THBS2, TIMP3, TNFAIP6, VCAN*
EIF2AK2	Inhibited	-2.573	< 0.0001	*ATF3, CEBPD, EGR1, IFIT1, IFIT5, IFITM1, ISG15, NEDD9, OAS1, TNFAIP3, USP18*
IRF1	Inhibited	-3.058	< 0.0001	*CCL2, CTSS, CXCL16, IFI44L, IFIT1, IFIT5, IFITM1, IRF2, ISG15, MX1, OAS1, RSAD2, TGFB1, TLR6*
TAZ	Inhibited	-2.167	< 0.0001	*AMOTL2, ANKRD1, CCL2, CCN1, CCN2, COL1A1, LUM, MET, SERPINE1, TAGLN, TGFB1*
IFIH1	Inhibited	-2.190	< 0.0001	*CCL2, EGR1, IFI44L, IFIT1, ISG15, OAS1, RND3, RSAD2*
BMP2	Inhibited	-2.052	< 0.0001	*ACTA2, BMP2, COL1A1, COL1A2, FOSB, FSHB, ID1, IGFBP5, JUNB, POSTN, RAD1, TAGLN, TG, TIMP3, VCAN*
IFNA2	Inhibited	-3.533	< 0.0001	*CCL2, HERC6, IFI44L, IFIT1, IFIT5, IFITM1, IFNAR2, ISG15, MET, MX1, OAS1, PLAT, RSAD2, SDC1, TREX1, USP18*
TLR4	Inhibited	-2.309	< 0.0001	*ATF3, BMP2, CCL2, CCN1, CCR5, CD14, CEBPD, CERS5, CITED2, IRF2, ISG15, LTC4S, MARCH3, MET, MX1, PLAT, RGCC, RSAD2, TGFB1, TNFAIP3*

CEBPB	Inhibited	-2.547	< 0.0001	*ACTA2, CCL2, CCNE1, CCR5, CD14, CD1D, CEBPD, COL1A1, COL1A2, GAS1, ID1, KRT18, PLG, RAC2, SERPINE1, SIM2, TGFB1, TMEM176B, TNFAIP6, UBA2, VCAN*
Brd4	Inhibited	-2.813	< 0.0001	*ACTA2, COL1A1, COL1A2, PLAT, SPON2, SPTA1, TAGLN, TLR6*
Jnk	Inhibited	-3.059	< 0.0001	*ACTA2, CCL2, CCN2, CCNE1, COL1A1, EGR1, FOSB, PLAT, PLG, POSTN, SERPINE1, SNAI2, TGFB1, TNFRSF12A, VCAN*
OSM	Inhibited	-2.475	< 0.0001	*ACTA2, ANXA2, ATF3, CCL2, CEBPD, CHD1, COL1A1, COL1A2, CTSH, DHRS7, EGR1, FOSB, GPNMB, ID1, JUNB, KRT8, LITAF, MAOA, MAP2, MX1, MYEF2, OAS1, SERPINE1, SON, TIMP3*
Y 27632	Activated	2.190	< 0.0001	*ACTA2, AMOTL2, BMP2, CCL2, CCN2, CLDN5, SERPINE1, TAGLN, TGFB1*
cisplatin	Inhibited	-2.637	< 0.0001	*ACTA2, AGXT2, ANKRD1, ANXA2, ATF3, CCL2, CCN2, CCR5, CD14, CD68, COL1A1, DNAH5, EGR1, GAS1, GPNMB, IFITM1, IRF2, MAOA, MET, MX1, PLAT, SERPINE1, SULF2, TCIM, TGFB1, TIMP3, TNFRSF12A, TNFRSF1B, TP63*
GPER1	Inhibited	-2.789	< 0.0001	*ATF3, CCN1, CCN2, CEBPD, EGR1, FOSB, TNFAIP3, TUFT1*
Interferon alpha	Inhibited	-3.481	< 0.0001	*ATF3, CCL2, CCR5, CD1D, FCGR1A, HERC6, Ifi27, IFI44L, IFIT1, IFITM1, IFNAR2, IL9, ISG15, MEFV, MX1, OAS1, PARP10, PLAT, RSAD2, TGFB1, USP18*
EDN1	Inhibited	-3.180	< 0.0001	*ACTA2, CCL2, CCN1, CCN2, COL1A2, EGR1, FOSB, JUNB,*

				NRG1, OLR1, SDC1, SERPINE1, TIMP3, VCAN
SEMA7A	Inhibited	-2.449	< 0.0001	*CCN2, COL1A1, COL1A2, CTSS, EGR1, TGFB1*
salmonella minnesota R595 lipopolysaccharides	Inhibited	-2.047	< 0.0001	*CCL2, CEBPD, EGR1, FOSB, ISG15, JUNB, MEFV, MET, RSAD2, SERPINE1, TNFAIP3*
oblimersen	Inhibited	-2.828	< 0.0001	*ATF3, CEBPD, IFIT1, IFITM1, ISG15, MX1, OAS1, RSAD2*
Smad	Inhibited	-2.155	< 0.0001	*ANGPTL4, CCN2, COL1A1, COL1A2, ID1, SERPINE1*
PTK2	Inhibited	-2.192	< 0.0001	*ACTA2, CCN1, COL1A1, COL1A2, CSPG4, KRT18, KRT8, TIMP3*
Pkc(s)	Inhibited	-2.914	< 0.0001	*ACTA2, ATF3, CCL2, CCN1, CCN2, CCR5, EGR1, FSHB, JUNB, KRT8, SERPINE1, STC1, TAGLN, TGFB1*
IGF1	Inhibited	-2.113	< 0.0001	*ACTA2, APLF, BMP2, CCN1, CCN2, CCNE1, CEBPD, CITED2, COL1A1, EGR1, FOSB, ID1, IGFBP5, JUNB, PCK2, SERPINE1, SLC12A6, SNAI2, TG, TGFB1, TNFRSF12A*
STAT1	Inhibited	-3.086	< 0.0001	*APOE, CCL2, CCNE1, CD14, CEBPD, CTSS, EGR1, FCGR1A, HERC6, IFI44L, IFIT1, IFITM1, IRF2, ISG15, MX1, OAS1, RSAD2, USP18*
AKT1	Inhibited	-2.283	< 0.0001	*ACTA2, ANKRD1, BMP2, C1QC, CCL2, CCNE1, CEBPD, EGR1, FOSB, HNRNPM, IGFBP5, PLG, SERPINE1, SNAI2, THBS2, VCAN*
PI3K (complex)	Inhibited	-2.579	< 0.0001	*ATF3, CCL2, CCN2, CCNE1, COL1A1, FSHB, IFIT1, IRF2, MAP2, POSTN, RND3, SERPINE1, SLC12A6, TAGLN, TNFAIP3, TP63*
TGFBR1	Inhibited	-2.372	< 0.0001	*ACTA2, CCN2, CLDN5, COL1A1, COL1A2, GAS1, SERPINE1, SNAI2, TAGLN*

AGT	Inhibited	-4.189	< 0.0001	*ACTA2, ATF3, CCL2, CCN2, CCNE1, COL1A1, COL1A2, CTSS, EGR1, FCGR1A, IGFBP5, MAP2, NRG1, OLR1, POSTN, SERPINE1, STC1, TGFB1, TNFRSF12A, TNFRSF1B*
SMAD2	Inhibited	-2.352	< 0.0001	*ACTA2, CCN2, COL1A2, FSHB, IL9, MET, SERPINE1, SNAI2, TIMP3*
SMARCA4	Inhibited	-3.748	< 0.0001	*ACTA2, BEND5, CA14, CCL2, CCN2, COL1A1, CTSH, CTSS, EGR1, GMFG, IFIT1, IFITM1, IGFBP5, KRT18, LUM, Pdlim3, PLAT, RAC2, RAD1, SDC2, SERPINE1, TAGLN, TCIM, TMEM204*
MYD88	Inhibited	-2.893	< 0.0001	*CCL2, CCN1, CD14, CEBPD, EGR1, IGFBP5, ISG15, MEFV, MET, OLR1, RSAD2, SERPINE1, TGFB1, TNFAIP3, USP18*
IFN Beta	Inhibited	-2.813	< 0.0001	*ATF3, IFIT1, IFITM1, IFNAR2, IL9, ISG15, MX1, OAS1, RSAD2, TGFB1, USP18*
TLR9	Inhibited	-2.010	< 0.0001	*ATF3, EGR1, IFI44L, IFIT1, IFITM1, ISG15, MX1, NTS, RSAD2, SDC1, SERPINE1, TGFB1, TNFRSF1B, USP18*
ABCB4	Activated	2.418	< 0.0001	*ACTA2, CCN2, COL1A1, COL1A2, SERPINE1, TGFB1*
GLIS2	Activated	2.236	< 0.0001	*CCN2, COL1A1, SERPINE1, SNAI2, TGFB1*
ethanol	Inhibited	-2.636	< 0.0001	*ACTA2, ATF3, C5AR1, CCL2, CCN2, CD14, CEBPD, COL1A2, EGR1, FOSB, ID1, MAP2, PLAT, RGCC, SERPINE1, TGFB1, TLR6, TNFAIP6*
CR1L	Activated	2.412	< 0.0001	*CCN2, COL1A1, COL1A2, LUM, SERPINE1, TGFB1*
4-hydroxytamoxifen	Inhibited	-2.026	< 0.0001	*ATF3, CCN1, CCN2, CEBPD, EGR1, FOSB, JUNB, NEDD9, SLC39A8, SNAI2, TNFAIP3, TUFT1*

SRC (family)	Inhibited	-2.608	< 0.0001	*BMP2, CCL2, EGR1, ID1, MAOA, S100A4, TGFB1*
TLR7	Inhibited	-3.383	< 0.0001	*ATF3, CCL2, CD1D, FOXN4, IFI44L, IFIT1, IFITM1, ISG15, JUNB, MX1, PLAT, RSAD2, TNFAIP3*
bromodeoxyuridine	Inhibited	-2.630	< 0.0001	*IFIT1, IFITM1, IL9, ISG15, MX1, OAS1, SERPINE1*
TSC2	Activated	2.975	< 0.0001	*ANXA2, ATF3, CD68, EGR1, GPNMB, GSN, IGFBP5, PKIB, TAGLN, TMEM176B*
EGR1	Inhibited	-2.315	< 0.0001	*ACTA2, ATF3, CAVIN1, CCL2, COL1A1, COL1A2, EGR1, ID1, JUNB, SERPINE1, SNAI2, TGFB1*
FBN1	Activated	2.213	< 0.0001	*CCN1, CCN2, COL1A2, SERPINE1, TIMP3*
P38 MAPK	Inhibited	-2.608	< 0.0001	*ATF3, BMP2, CCL2, CCN2, CCNE1, CD1D, CEBPD, CLDN5, EGR1, IGFBP5, JUNB, POSTN, RND3, SDC2, SERPINE1, TGFB1, TIMP3*
F2R	Inhibited	-2.416	< 0.0001	*CCL2, CCN1, CCN2, EGR1, KRT8, PLAT, S100A4, TAGLN, TNFAIP3*
CD38	Inhibited	-2.558	< 0.0001	*ACTA2, ANXA2, CCNE1, CITED2, COL1A1, LFNG, PKIB, S100A4, SDC1, SLC39A8, TGFB1*
IPMK	Inhibited	-2.236	< 0.0001	*ACTA2, CCN1, FOSB, JUNB, TAGLN2*
NFkB (complex)	Inhibited	-3.366	< 0.0001	*ANKRD1, APOE, ATF3, BMP2, CCL2, CCN1, CCN2, CEBPD, COL1A2, EGR1, FCGR1A, IFNAR2, ISG15, JUNB, LITAF, LTC4S, OLR1, RSAD2, SERPINE1, SNAI2, TGFB1, TNFAIP3, TNFAIP6*
RETNLB	Inhibited	-2.813	< 0.0001	*ACTA2, CCL2, CCN2, CCR5, COL1A1, COL1A2, DNER, TGFB1*
SFTPA1	Activated	2.828	< 0.0001	*ANGPTL4, CCN1, CCN2, COL1A1, EGR1, FOSB, SERPINE1, TGFB1*
HIF1A	Inhibited	-2.39	< 0.0001	*ACTA2, ANGPTL4, APOE, BMP2, CCN1, CCN2, CCR5,*

				CITED2, FHL3, HIST1H2AM, ID1, IGFBP5, KRT18, MET, SERPINE1, SNAI2, TGFB1, TLR6
deferoxamine	Inhibited	-2.137	< 0.0001	*ANGPTL4, ANXA2, CCNE1, CD14, CITED2, IFIT5, IGFBP5, PLAT, RSAD2, SERPINE1, SULF2, TGFB1*
SOCS1	Activated	3.113	< 0.0001	*CCL2, CCNE1, CD1D, H2-T24, IFIT1, ISG15, MX1, OAS1, RSAD2, USP18*
ERK1/2	Inhibited	-3.092	< 0.0001	*BMP2, CCL2, CCN2, COL1A1, EGR1, FOSB, FSHB, ID1, IFIT1, JUNB, POSTN, SERPINE1, SLC12A6, SNAI2, VCAN*
IL17A	Inhibited	-2.21	< 0.0001	*ACTA2, CCL2, CCN1, CCN2, CD14, CD68, CEBPD, COL1A1, IL9, ISG15, TNFAIP3, TNFRSF1B, TP63*
SMARCB1	Inhibited	-2.828	< 0.0001	*CCNE1, COL1A1, COL1A2, FCGR1A, GSN, IFITM1, IFNAR2, MX1, OAS1, POSTN, RND3*
SP1	Inhibited	-2.635	< 0.0001	*ACTA2, APOE, ATF3, CCL2, CCN2, CEBPD, CHRNA5, CITED2, COL1A1, COL1A2, DLC1, EGR1, KRT18, MAOA, MET, SERPINE1, SLC39A8, TAGLN, TGFB1, TIMP3, TNFAIP3*
ERK	Inhibited	-2.978	< 0.0001	*CCL2, CCN2, CCNE1, CD1D, CEBPD, COL1A1, EGR1, IFNAR2, JUNB, KRT18, SERPINE1, SNAI2, VCAN*
TICAM1	Inhibited	-2.003	< 0.0001	*CCL2, CEBPD, EGR1, IFIT1, ISG15, MEFV, MET, RSAD2, SERPINE1, TNFAIP3*
SOCS3	Activated	2.592	< 0.0001	*ATF3, EGR1, FCGR1A, IFIT1, MX1, OAS1, TGFB1, TNFRSF1B*
IRAK3	Inhibited	-2.219	< 0.0001	*ACTA2, CCN2, COL1A1, TGFB1, TNFAIP3*
CG	Inhibited	-3.759	< 0.0001	*ACTA2, BMP2, CCL2, CEBPD, EGR1, GAS1, MAF, MARCH3, MMP19, PLAT,*

				SDC1, ST8SIA4, STC1, TNFAIP6, TNFRSF12A, VCAN
BCL6	Activated	2.178	< 0.0001	*CCL2, COL1A1, HERC6, IL9, JMY, JUNB, LITAF, MAF, SDC1, SERPINE1, SLC39A8, TGFB1*
APOE	Activated	2.375	< 0.0001	*ACTA2, APOE, ATF3, C5AR1, CCN1, CCN2, CCR5, CD68, COL1A1, CTSS, EGR1, FCGR1A, MAP2, SERPINE1, TGFB1*
NR3C2	Inhibited	-2.377	< 0.0001	*CCL2, CCN2, COL1A1, EGR1, JUNB, MAP2, PLAT, SERPINE1, TGFB1*
IL5	Inhibited	-3.719	< 0.0001	*ANXA2, CCL2, CCNE1, CCR5, CD9, CITED2, EGR1, IL9, LFNG, LUM, PKIB, S100A4, SLC39A8, TGFB1*
sphingosine-1-phosphate	Inhibited	-2.560	< 0.0001	*ACTA2, ANXA2, CCL2, CCN2, EGR1, SERPINE1, TAGLN, TGFB1*
IFNAR1	Inhibited	-2.163	< 0.0001	*ATF3, IFITM1, ISG15, OAS1, PLAT, RSAD2, SDC1, SERPINE1, TNFAIP3, USP18*
Alpha catenin	Activated	2.960	< 0.0001	*COL1A1, COL1A2, LUM, MMP19, STEAP2, THBS2, TNFAIP3, TNFAIP6, TNFRSF12A*
IFNA4	Inhibited	-2.357	< 0.0001	*H2-T24, IFIT1, ISG15, MX1, RSAD2, USP18*
stallimycin	Inhibited	-2.433	< 0.0001	*IFIT1, IFITM1, IL9, ISG15, MX1, OAS1*
SPDEF	Activated	2.611	< 0.0001	*CCN2, COL1A1, SDC1, SDC2, SERPINE1, SNAI2, TGFB1*
cytokine	Inhibited	-2.377	< 0.0001	*C5AR1, CCL2, CCN1, CCNE1, EGR1, PLAT, S100A4, SERPINE1, TNFAIP6, TNFRSF1B*
PPARGC1A	Activated	3.121	< 0.0001	*CCN2, CD68, CDC42EP1, CEBPD, COL1A2, IGFBP5, LUM, MMP19, OLR1, PCK2, POSTN, STC1, TIMP3, TNFRSF12A, VCAN*
SPI1	Inhibited	-2.713	< 0.0001	*C1QC, CD14, CD1D, CD68, CTSS, IFI44L, IFIT1, IFITM1,*

				IL9, ISG15, MX1, RSAD2, USP18
IL1	Inhibited	-2.165	< 0.0001	*APOE, BMP2, CCL2, CEBPD, COL1A1, IL9, KRT72, NRG1, SERPINE1, TG, TGFB1, TNFAIP3, TNFAIP6, TNFRSF1B, VCAN*
Pdgf (complex)	Inhibited	-2.762	< 0.0001	*CCL2, EGR1, FOSB, JUNB, PIK3R3, SERPINE1, TGFB1, TIMP3*
Growth hormone	Inhibited	-2.760	< 0.0001	*ANGPTL4, APOE, BMP2, CEBPD, CITED2, COL1A1, EGR1, ID1, IGFBP5, JUNB, NEDD9, STC1*
PTH	Inhibited	-2.046	< 0.0001	*BMP2, CCL2, CCN2, COL1A1, COL1A2, FOSB, IGFBP5, POSTN, STC1, TGFB1*
CXCL12	Inhibited	-3.016	< 0.0001	*ACTA2, BMP2, CCL2, CD14, CD9, COL1A1, EGR1, ID1, IFNAR2, JUNB, TNFRSF1B*
ALDH1A2	Activated	2.236	< 0.0001	*ATF3, CCN1, CCN2, COL1A2, TIMP3*
curcumin	Activated	2.855	0.0001	*ACTA2, APOE, CCL2, CCN2, CCNE1, COL1A1, COL1A2, EGR1, FOSB, IGFBP5, JUNB, OLR1, SERPINE1, SIM2, TIMP3*
anisomycin	Inhibited	-2.395	0.0001	*ATF3, EGR1, JUNB, POSTN, SDC1, SERPINE1*
BMP4	Inhibited	-2.070	0.0001	*ACTA2, BMP2, CCN1, ID1, JUNB, POSTN, SMAD9, SNAI2, TAGLN, TP63, TRPV2*
IRF7	Inhibited	-3.061	0.0001	*FCGR1A, IFI44L, IFIT1, IFITM1, ISG15, MX1, OAS1, RSAD2, TREX1, USP18*
IFNA1/IFNA13	Inhibited	-2.387	0.0001	*IFIT1, IFITM1, ISG15, MX1, OAS1, RSAD2*
SETD2	Activated	2.236	0.0001	*CCN1, CCN2, CITED2, PLG, SERPINE1*
RAC1	Inhibited	-2.755	0.0001	*ACTA2, ANKRD1, ATF3, CCN2, COL1A2, GSN, SNAI2, TGFB1*
CCR2	Inhibited	-2.598	0.0001	*C1QC, CCL2, CCR5, COL1A1, COL1A2, LUM, TGFB1, VCAN*

MTPN	Inhibited	-2.63	0.0001	*AHSG, COL1A1, COL1A2, PLAT, SERPINE1, TAGLN, TGFB1*
GNRH1	Inhibited	-2.410	0.0001	*EGR1, FOSB, FSHB, IGFBP5, JUNB, PLAT*
CAV1	Inhibited	-2.183	0.0001	*Ang4, CAVIN1, CCNE1, CD14, EGR1, ID1, KRT18, KRT8, SERPINE1*
losartan potassium	Activated	2.270	0.0001	*CCN2, COL1A1, EGR1, MAP2, OLR1, POSTN, SERPINE1, TGFB1*
GDF9	Inhibited	-2.180	0.0001	*CCN2, FSHB, ID1, TNFAIP6, VCAN*
S-adenosylmethionine	Activated	2.177	0.0001	*ACTA2, COL1A1, COL1A2, MAF, TGFB1*
IFNB1	Inhibited	-2.130	0.0001	*CCL2, CCR5, CD14, GAS1, IFIT1, IFITM1, ISG15, MX1, OAS1, RND3, RSAD2, THBS2, USP18*
OGA	Inhibited	-2.000	0.0001	*AIFM2, AKT1S1, APOE, C6orf15, CCL2, CCR5, CD14, CD68, CXCL16, GAS1, GSN, IGFBP5, PRKAR1B, TGFB1, TIMP3, TNFRSF12A*
HOXD3	Inhibited	-2.000	0.0001	*COL1A1, SDC1, SERPINE1, TAGLN*
15-deoxy-delta-12, 14 -PGJ 2	Activated	2.269	0.0001	*ACTA2, CCL2, CCN2, CEBPD, COL1A1, COL1A2, EGR1, GSN, SERPINE1, TG*
cholecalciferol	Inhibited	-2.042	0.0001	*ANXA2, CCL2, CD14, CEBPD, COL1A1, SERPINE1, SULF2, TGFB1*
MAP2K1	Inhibited	-2.596	0.0001	*ACTA2, APOE, ATF3, CCL2, CCN2, COL1A1, COL1A2, GPRC5B, SIRPA, SNAI2*
TSLP	Inhibited	-2.000	0.0001	*ACTA2, COL1A2, IL9, MX1, TGFB1*
pimagedine	Activated	2.219	0.0001	*ACTA2, CCN2, SERPINE1, TAGLN, TGFB1*
imiquimod	Inhibited	-2.426	0.0001	*IFITM1, ISG15, JUNB, MX1, OAS1, RSAD2, TGFB1*
SRF	Inhibited	-2.092	0.0002	*ACTA2, ANKRD1, BMP2, C1QL3, CCN2, CEBPD, CLDN5, EGR1, FOSB, GSN, JUNB, RND3, TAGLN, VCAN*

SAMSN1	Inhibited	-2.646	0.0002	*CITED2, IRF2, ISG15, PLAT, RGCC, RSAD2, SDC1*
dalfampridine	Inhibited	-2.236	0.0002	*ATF3, CCN1, EGR1, FOSB, JUNB*
laminaran	Inhibited	-2.236	0.0002	*IFI44L, OLR1, RSAD2, SLC39A8, TNFAIP6*
vitamin E	Activated	2.425	0.0002	*ACTA2, CCL2, EGR1, PLAT, SERPINE1, TGFB1*
SNCA	Inhibited	-2.143	0.0002	*C1QL3, CCL2, CD68, CTHRC1, Cutal, GSN, ISG15, MX1, RSAD2, SERPINE1, STX1B*
D-fructose	Inhibited	-2.236	0.0002	*ACTA2, CD68, COL1A1, TGFB1, TIMP3*
KITLG	Inhibited	-2.778	0.0002	*CCL2, CCN2, CCNE1, CD14, CD68, EGR1, IL9, IRF2, LAPTM5, LTC4S, SNAI2*
testosterone	Inhibited	-2.596	0.0002	*ANKRD1, CCNE1, EGR1, FOSB, FSHB, ID1, IGFBP5, MAOA, MX1, PKIB, SDC1, SLC39A8, TG, VCAN*
Ap1	Inhibited	-2.412	0.0002	*ATF3, CCL2, CCN2, FSHB, KRT18, MET, S100A4, SERPINE1, TGFB1*
JAK1	Inhibited	-2.425	0.0002	*CCL2, CD14, ID1, JUNB, MX1, USP18*
SMAD1	Inhibited	-2.207	0.0002	*ACTA2, CCN2, COL1A1, COL1A2, ID1*
actinomycin D	Activated	2.243	0.0003	*CCL2, CCN1, CCN2, COL1A2, FSHB, IGFBP5, JMY, MAOA, SERPINE1, TGFB1, TNFRSF1B*
D-glucose	Inhibited	-3.466	0.0003	*ACTA2, ATF3, BMP2, CCL2, CCN2, CCNE1, COL1A1, EGR1, GSN, ID1, JUNB, KIF5C, NTS, PCK2, S100A4, SERPINE1, SLC39A8, TAGLN, TAGLN2, TGFB1*
Vegf	Inhibited	-2.877	0.0003	*ACTA2, ANGPTL4, ATF3, BMP2, CCN1, EGR1, FOSB, GPRC5B, ID1, MET, NDP, NRG1, RGCC, RNF125, ST8SIA4, STC1, TGFB1*
FOXO3	Inhibited	-2.137	0.0003	*BMP2, CCN1, CCN2, CERS5, EGR1, FOSB, FSHB, JUNB,*

				MET, SERPINE1, TCIM, TGFB1, TNFRSF1B
hydrogen peroxide	Inhibited	-3.928	0.0003	*ACTA2, ANKRD1, ANXA2, ATF3, CCL2, CCN2, CCR5, CD68, COL1A1, COL1A2, CTSS, EGR1, SERPINE1, SNAI2, STC1, TAGLN, TNFAIP3*
estrogen	Inhibited	-2.218	0.0003	*APOE, BMP2, CCL2, CCN1, CCN2, COL1A1, EGR1, Ifi27, IGFBP5, JUNB, NTS, PLAT*
AVP	Inhibited	-2.176	0.0003	*ACTA2, ATF3, CCL2, EGR1, TGFB1*
CREB1	Inhibited	-2.876	0.0004	*APOE, ARHGAP35, ATF3, CCN1, CD68, CD9, CEBPD, EGR1, FOSB, GPNMB, ID1, JUNB, KLF7, LITAF, PLAT, STC1, TAGLN2, TG*
PAF1	Inhibited	-2.236	0.0004	*CITED2, IFI44L, ISG15, SERPINE1, ZNFX1*
aldosterone	Inhibited	-2.770	0.0004	*BMP2, CCL2, CCN2, COL1A1, COL1A2, OLR1, SERPINE1, TGFB1*
CDKN2A	Inhibited	-2.271	0.0005	*ACTA2, CCL2, CCN2, CCNE1, CEBPD, CITED2, COL1A2, EGR1, IGFBP5, SERPINE1, TNFRSF1B, TP63*
Pka	Inhibited	-2.714	0.0005	*APOE, CCN2, CD14, EGR1, FSHB, IL9, JUNB, NTS*
SPARC	Inhibited	-2.063	0.0005	*CEBPD, COL1A1, COL1A2, MAP2, SERPINE1, SNAI2, TGFB1*
CSF1	Inhibited	-2.530	0.0005	*APOE, CCL2, CCNE1, CCR5, CD68, EGR1, JUNB, TLR6, TNFAIP3, TNFRSF1B*
E. coli B5 lipopolysaccharide	Inhibited	-2.720	0.0005	*AHSG, CCL2, CCR5, IFIT1, IL9, JUNB, SDC1, SERPINE1, TGFB1, TLR6, TNFAIP3*
MAP3K1	Inhibited	-2.186	0.0006	*ATF3, EGR1, LOXL3, SERPINE1, TGFB1*
IL27	Inhibited	-2.020	0.0006	*CCL2, CD14, CEBPD, EGR1, IL9, MAF, MX1, OAS1*
NR4A1	Activated	2.030	0.0006	*ACTA2, APOE, ATF3, CLDN5, COL1A1, COL1A2, FSHB, KRT18, KRT8, PCK2, SERPINE1*

FGF10	Inhibited	-2.200	0.0007	*ANXA2, CTSH, LFNG, TGFB1, TIMP3*
HOXC8	Activated	2.000	0.0007	*ANKRD1, GAS1, MSLN, TAGLN*
MAPK14	Inhibited	-2.360	0.0007	*ACTA2, ANKRD1, BMP2, CCL2, CEBPD, COL1A1, JUNB, TAGLN, TGFB1*
NOTCH3	Inhibited	-2.048	0.0007	*ACTA2, ID1, SDC2, SNAI2, TAGLN*
RARA	Activated	2.003	0.0007	*CD14, CD9, CTSS, EGR1, HOXA2, IFI44L, IL9, NEDD9, OAS1, RSAD2, TG, TGFB1, TP63*
TAC1	Inhibited	-2.449	0.0008	*CCL2, CCN1, FOSB, PLAT, SERPINE1, TGFB1*
amphetamine	Inhibited	-2.191	0.0008	*ATF3, EGR1, FOSB, JUNB, NTS, TNFAIP3*
cocaine	Inhibited	-2.921	0.0008	*CCL2, CCR5, EGR1, FOSB, IFIT1, JUNB, MX1, NTS, OAS1*
IL1A	Inhibited	-3.073	0.0009	*ATF3, CCL2, CCN1, FOSB, IGFBP5, IL9, JUNB, SERPINE1, TGFB1, TNFAIP3*
NORAD	Inhibited	-2.000	0.0010	*JUNB, SDC1, TAGLN, TNFRSF12A*
SOX2	Activated	2.095	0.0011	*ATF3, CCN2, CD9, CITED2, COL1A1, GSN, KRT18, Pdlim3, PIK3R3, SERPINE1, TGFB1, TIMP3, TP63, USP18, VCAN*
ATF4	Inhibited	-2.388	0.0011	*ANKRD1, ATF3, CCL2, CSRP3, IGFBP5, PCK2, SNAI2, TNFRSF12A*
Ige	Inhibited	-2.905	0.0011	*CCL2, CCR5, CXCL16, EGR1, FCGR1A, GSN, IL9, JUNB, RSAD2, TGFB1, TNFRSF12A, TNFRSF1B*
PTGS2	Inhibited	-2.621	0.0013	*ANXA2, CCL2, CCNE1, CD68, EGR1, ID1, STC1, TNFAIP6*
FOXL2	Inhibited	-2.177	0.0014	*ATF3, BMP2, FSHB, GPRC5B, TNFAIP3*
FOXO1	Inhibited	-2.347	0.0014	*AIFM2, ANGPTL4, CCN2, CERS5, CITED2, CLDN5, EGR1, FOSB, FSHB, JUNB, KLF7, PCK2, SERPINE1, TGFB1*

SREBF1	Inhibited	-2.236	0.0015	*C5AR1, CD14, CEBPD, GPNMB, MLYCD, PCK2, PIK3R3, SERPINE1, TGFB1, TNFRSF1B*
TRIM24	Activated	2.213	0.0017	*HERC6, ISG15, MOV10, OAS1, SERPINE1, USP18*
TNFSF10	Inhibited	-2.393	0.0017	*CD14, IFIT1, IFITM1, ISG15, TNFAIP3, TP63*
LIF	Inhibited	-2.214	0.0019	*ACTA2, AHSG, BMP2, CCNE1, CD9, CEBPD, EGR1, JUNB, TMEM176B*
INSIG1	Activated	2.449	0.0019	*APOE, CCR5, CD68, OLR1, STEAP2, TGFB1*
EPAS1	Inhibited	-2.205	0.0019	*ANGPTL4, CCN1, CCN2, CCR5, CITED2, IGFBP5, PKIB, SERPINE1, TNFAIP3*
E. coli lipopolysaccharide	Inhibited	-2.17	0.0020	*CCL2, CCN1, CEBPD, MX1, TGFB1*
NFATC3	Inhibited	-2.000	0.0020	*ACTA2, ATF3, BMP2, COL1A1, FOSB*
CEBPA	Inhibited	-2.173	0.0020	*CD14, CEBPD, COL1A1, COL1A2, FCGR1A, GAS1, ID1, ISG15, JUNB, LITAF, OLR1, SERPINE1, TNFAIP6, VCAN*
DDX58	Inhibited	-2.192	0.0021	*CCL2, IFIT1, ISG15, OAS1, RSAD2*
GLI3	Inhibited	-2.219	0.0021	*EGR1, FOSB, JUNB, TNFAIP3, TP63*
TNFSF11	Inhibited	-2.376	0.0021	*CCL2, CCNE1, CD14, IL9, JUNB, SDC1, SERPINE1, TGFB1, TNFRSF1B, USP18*
JAK	Inhibited	-2.000	0.0021	*CCL2, IFIT1, ISG15, RSAD2*
ACKR2	Activated	2.000	0.0021	*ISG15, OAS1, RSAD2, USP18*
Ins1	Inhibited	-2.728	0.0022	*ANGPTL4, CA14, CCNE1, CEBPD, COL1A1, EGR1, IGFBP5, JUNB, PLAT, SERPINE1, TIMP3*
palmitic acid	Inhibited	-2.924	0.0023	*AHSG, ATF3, CCL2, CCN2, CD68, MET, OLR1, SERPINE1, TGFB1*
topotecan	Inhibited	-2.200	0.0024	*ATF3, CCNE1, CHD1, CITED2, EGR1, FOSB, JUNB, SON*
L-glutamic acid	Inhibited	-2.133	0.0024	*ACTA2, CCNE1, EGR1, FOSB, JUNB, MAP2*

SQSTM1	Inhibited	-2.000	0.0027	*CITED2, PLAT, RGCC, RSAD2*
IL33	Inhibited	-2.938	0.0035	*APOE, CCL2, CXCL16, IL9, JUNB, PLAT, TGFB1, TNFAIP3, TNFRSF1B*
methylprednisolone	Inhibited	-2.150	0.0036	*ANXA2, CCN2, CD9, CITED2, CTSH, CTSS, ID1, MYO1G, PLAT, RGCC, RND3, SDC1, SERPINE1, SIRPA, TIMP3*
BTK	Activated	2.236	0.0037	*CCNE1, IFI44L, IFIT1, IFITM1, ISG15, MX1*
let-7a-5p (and other miRNAs w/seed GAGGUAG)	Activated	2.569	0.0037	*ACTA2, CCNE1, COL1A1, COL1A2, IFIT5, S100A4, TAGLN*
H2AFB3 (includes others)	Inhibited	-2.000	0.0040	*ATF3, CSRP3, GAS1, GMFG*
norepinephrine	Inhibited	-2.630	0.0061	*CCN2, COL1A2, EGR1, GALNT14, ID1, SERPINE1, TGFB1*
MYB	Inhibited	-2.169	0.0061	*CCNE1, COL1A2, IGFBP5, RGCC, SDC1, SNAI2*
Ifnar	Inhibited	-2.213	0.0069	*CCL2, ISG15, OAS1, RSAD2, USP18*
HOXD10	Inhibited	-2.000	0.0084	*AGXT2, RSAD2, SERPINE1, ST8SIA2*
TGM2	Inhibited	-2.789	0.0084	*C5AR1, CCL2, IFIT1, IFIT5, OAS1, SIRPA, TGFB1, TYROBP*
gentamicin C	Inhibited	-2.000	0.0089	*ANXA2, CD14, GPNMB, TNFRSF12A*
lomustine	Inhibited	-2.000	0.0089	*ANXA2, CD14, GPNMB, TNFRSF12A*
filgrastim	Activated	2.967	0.0112	*ATF3, DLC1, IFIT1, IFIT5, ISG15, MPEG1, MX1, RSAD2, S100A4, SPON2, SULF2, TREX1*
DOCK8	Inhibited	-2.000	0.0115	*CITED2, ISG15, PLAT, RSAD2*
peptidoglycan	Inhibited	-2.201	0.0136	*CCL2, CCR5, CXCL16, EGR1, JUNB*
5-hydroxytryptamine	Inhibited	-2.195	0.0141	*CCL2, CCN2, EGR1, SERPINE1, TGFB1*
LCN2	Inhibited	-2.000	0.0146	*BMP2, COL1A1, TAGLN2, TGFB1*
Bvht	Inhibited	-2.000	0.0152	*ACTA2, CSRP3, MMP19, SNAI2*

EGLN	Activated	2.415	0.0175	*CAVIN1*, *CCL2*, *NTS*, *SERPINE1*, *STC1*, *TNFAIP6*
SPIB	Inhibited	-2.000	0.0221	*APOE*, *ATF3*, *CCR5*, *EGR1*
FOXA2	Activated	2.000	0.0225	*ACTA2*, *CCR5*, *CTHRC1*, *IGFBP5*, *LTC4S*, *TAGLN*, *TGFB1*
allopurinol	Inhibited	-2.000	0.0230	*ANXA2*, *CD14*, *GPNMB*, *TNFRSF12A*
STAT4	Inhibited	-2.401	0.0251	*CCL2*, *CCR5*, *ISG15*, *KRT18*, *RGCC*, *S100A4*, *SERPINE1*
CpG oligonucleotide	Inhibited	-2.236	0.0377	*CD14*, *EGR1*, *OLR1*, *SDC1*, *SERPINE1*
E2F1	Inhibited	-2.728	0.0525	*CCNE1*, *CD9*, *CITED2*, *EGR1*, *JMY*, *KRT1*, *MAF*, *RAD52*, *RND3*, *SERPINE1*, *TGFB1*
MYCN	Activated	2.020	0.0698	*AMOTL2*, *CCN2*, *CITED2*, *COL1A1*, *SDC2*, *SERPINE1*, *TAGLN*
HOXA10	Activated	2.236	0.0811	*APOE*, *CLDN5*, *ID1*, *IGFBP5*, *KRT18*
SOX4	Inhibited	-2.219	0.0886	*AIF1L*, *CD1D*, *CSRP3*, *DBNDD2*, *TMEM176B*
NOS2	Inhibited	-2.000	0.0933	*CCL2*, *CD14*, *ISG15*, *TGFB1*, *TYROBP*

Supplemental Table 15. Predicted upstream regulators of differentially expressed genes in oocytes from bacteria infused heifers at day 60.

**Upstream regulator**	**Predicted activation state**	**Activation z-score**	***P*-value of overlap**	**Target molecules in dataset**
Chorionic gonadotropin complex	Activated	2.993	< 0.0001	*BTC, CCND2, CLU, CYP19A1, EGR1, ENPP3, FOS, G0S2, GPC1, HPSE, HSD17B7, HSD3B2, IL33, LDLR, LGALS3BP, OXT, OXTR, PLPP3, PTX3, SDC4, ST8SIA4, TNFAIP6*
PRL	Activated	3.893	< 0.0001	*CLU, COL1A1, EGR1, ERBB3, FOS, HERC6, HSD17B7, IFI6, IFIT5, IRF9, ISG15, MFGE8, OAS1, OXT, PARP10, PDK4, RSAD2, SCP2, USP18*
OSM	Activated	2.708	< 0.0001	*BTC, C1S, CCND2, COL1A1, CYP19A1, DHRS7, DHX9, EGR1, ERBB3, FOS, HLA-B, IL33, IRF9, KLF10, KRT8, LDLR, MAGED2, MAOA, MX1, OAS1, PEPD, PTGES, SAA1, SERPING1, TGM2, TPBG, TUBA1A, UAP1*
IFNL1	Activated	3.260	< 0.0001	*HERC6, HLA-B, IFI6, IFIT5, IRF9, ISG15, LGALS3BP, MX1, OAS1, RSAD2, USP18*
IFNA2	Activated	3.580	< 0.0001	*C1S, HERC6, HLA-B, IFI6, IFIT5, IRF9, ISG15, LAPTM4B, LGALS3BP, MX1, OAS1, RSAD2, TGM2, THEMIS2, TNFRSF1A, UBA7, USP18*
EIF2AK2	Activated	3.130	< 0.0001	*EGR1, FOS, IFI6, IFIT5, ISG15, LGALS3BP, NMI, OAS1, TM4SF20, TUBA1A, USP18*
dihydrotestosterone	Activated	2.440	< 0.0001	*APOE, CCND2, CLU, CYP19A1, DEPDC1, EGR1, FOS, Foxp1, FRK, G0S2, HSD3B2, ITGA2, KLK4, LDLR, LPL, NR3C1, OXT, OXTR, PHGDH, POSTN, PROS1, RARRES2, SCP2, SNAI2, TGM2, UAP1*

TNF	Activated	2.759	< 0.0001	*ACADM, APOE, CCDC80, CCND2, CDH13, CLU, COL1A1, CYP19A1, CYP27A1, DCHS1, EGR1, ENPP3, FOS, G0S2, GPRC5B, HLA-B, HSD17B7, HSD3B2, IFI6, IFIT5, IL33, ISG15, ITGA2, KLF10, KRT8, LDLR, LGALS9, LPL, MAN1A2, MX1, NR3C1, OAS1, PHGDH, PLOD2, POSTN, PSMA2, PTGES, PTPRC, PTX3, RARRES2, RGS3, RND3, SAA1, SDC4, SLC27A5, SPSB1, ST8SIA4, TCIM, TGM2, THBS2, TNFAIP6, TNFRSF10A, TNFRSF1A, TSC22D3, VEGFD*
lipopolysaccharide	Activated	4.264	< 0.0001	*ACADM, APOE, CCND2, CD58, COL1A1, CYP27A1, EGR1, EHHADH, EPHX2, FOS, Foxp1, G0S2, HIVEP2, IFI6, IFIT5, IFNW1, IL2RG, IL33, IRF9, ISG15, KCNMB4, KLF10, LGALS3BP, LGALS9, LPL, LTA4H, MAOA, MX1, NR3C1, OAS1, PARP10, PARP3, PDK4, PLEK, PROS1, PSMA2, PTGES, PTX3, RND3, RSAD2, SAA1, SDC4, SERPINH1, SNX12, TCIM, TGM2, THBS2, TNFAIP6, TNFRSF1A, TNFSF18, TPBG, TSC22D3, USP18, XCL1*
poly rI:rC-RNA	Activated	3.153	< 0.0001	*BTC, CCND2, EGR1, FOS, HLA-B, IFIT5, IL2RG, IL33, IRF9, ISG15, LGALS9, LPL, LTA4H, MX1, NMI, OAS1, RSAD2, SDC4, SERPING1, SNAI2, ST8SIA4, TNFAIP6, TNFRSF10A, USP18*
IFNG	Activated	2.764	< 0.0001	*ADAMTS9, CCND2, CDH13, COL1A1, CYP27A1, DHX9, EGR1, FOS, GPRC5B, HERC6, HLA-B, IDH3B, IFI6, IFIT5, IRF9, ISG15, KCTD7,*

				KLF10, LGALS3BP, LGALS9, LPL, MX1, NMI, OAS1, PHGDH, PLEK, PSMA2, PTGES, PTX3, RAB38, RCVRN, RSAD2, SDC4, SERPING1, SERPINH1, SNAI2, THEMIS2, TNFAIP6, TNFRSF10A, TNFRSF1A, TSC22D3, USP18, VSNL1, XCL1
MAPK1	Inhibited	-2.331	< 0.0001	*APOE, C1S, EGR1, FOS, HLA-B, IFI6, IFIT5, IRF9, ISG15, LGALS3BP, LIG1, NMI, OAS1, PROS1, RBM45, SPSB1, TRANK1, TTC27, USP18*
Interferon alpha	Activated	3.209	< 0.0001	*CCND2, FOS, HERC6, HLA-B, Ifi27, IFI6, IRF9, ISG15, LGALS9, MCRS1, MX1, NMI, OAS1, PARP10, PTGES, RNASEL, RSAD2, TNFSF18, TRANK1, UBA7, USP18*
IL1RN	Inhibited	-2.714	< 0.0001	*HERC6, IFI6, IFIT5, IRF9, KCTD7, LGALS9, MX1, OAS1, RSAD2, THEMIS2, USP18*
TLR3	Activated	3.246	< 0.0001	*CLU, CSRP3, FOS, IFI6, IFNW1, IL33, ISG15, MX1, NMI, OAS1, PTGES, PTX3, RSAD2, TNFRSF1A, USP18*
IFNB1	Activated	2.400	< 0.0001	*FOS, IFI6, IRF9, ISG15, MX1, NMI, OAS1, PDK4, RNASEL, RND3, RSAD2, THBS2, TNFRSF10A, UBA7, USP18, ZDHHC14*
PPARA	Activated	2.912	< 0.0001	*ACADM, ANAPC5, APOE, C1S, CYP27A1, EHHADH, FADS2, FOS, G0S2, H2AFZ, KRT8, LPL, OXT, PDK4, PON1, RTN4, SAA1, SCP2, TDG, VNN1*
TRIM24	Inhibited	-2.975	< 0.0001	*HERC6, IRF9, ISG15, LGALS3BP, NMI, OAS1, PCLAF, UBA7, USP18*
estrogen	Activated	2.136	< 0.0001	*APOE, CLU, COL1A1, CYP19A1, EGR1, ERBB3,*

				FOS, Ifi27, LDLR, LIG1, LPL, OXT, OXTR, PSMA2, PTX3
IRF7	Activated	3.226	< 0.0001	*IFI6, IFNW1, IL33, IRF9, ISG15, MX1, NMI, OAS1, RSAD2, UBA7, USP18*
IL1B	Activated	3.722	< 0.0001	*APOE, COL1A1, CYP19A1, EGR1, FOS, G0S2, IL33, ISG15, KLF10, LDLR, LGALS9, MX1, NMI, OXTR, POSTN, PTGES, PTX3, RASA2, RSAD2, SAA1, SDC4, SERPINH1, TCIM, TGM2, TNFAIP6, TNFRSF10A, TSC22D3, UAP1, USP18, VSNL1*
USP18	Inhibited	-2.219	< 0.0001	*IFI6, IRF9, ISG15, MX1, OAS1*
TMEM173	Activated	2.425	< 0.0001	*HOPX, IL33, ISG15, OAS1, RSAD2, USP18*
decitabine	Activated	2.057	< 0.0001	*APOE, CCND2, CD58, CDH13, CLU, COL1A1, CYP19A1, FBN2, HLA-B, HMGN5, HOXC8, HPSE, ISG15, ITGA2, KRT8, LDLR, MX1, NR3C1, OAS1, RTN4, SERPINH1, SLC4A10, SRSF6, ST14, TNFRSF10A, XCL1*
tretinoin	Activated	4.279	0.0001	*APOE, BTC, CCNC, CCND2, COL1A1, DDAH2, EGR1, FBN2, FOS, FZD10, G0S2, HLA-B, IFI6, IFIT5, INSL3, IRF9, ISG15, ITGA2, LGALS3BP, LGALS9, NMI, NR3C1, OAS1, OXT, PLEK, PNP, POSTN, PTGES, PTPRC, PTX3, RARRES2, SERPING1, SLC38A2, STRA6, TGM2, TNFAIP6, TNFRSF10A, TNFRSF1A, TSC22D3, TTC27, UBA7, USP18*
Growth hormone	Activated	2.557	0.0001	*APOE, CCND2, CLU, COL1A1, EGR1, FOS, KLF10, LDLR, LPL, PDK4, PON1, TUBA1A*
IL4	Activated	3.147	0.0002	*APOE, CCND2, CD58, COL1A1, FOS, HSD3B2,*

				IL27RA, IL33, IPO8, IRF9, ISG15, ITGA2, LGALS3BP, LPL, LTA4H, MAOA, MYOF, NR3C1, PLOD2, PNP, POSTN, PROS1, PSMA2, RASGRP3, ST14, SYMPK, TGM2, TNFSF18, XCL1
RELA	Activated	2.015	0.0002	*APOE, COL1A1, CYP19A1, EGR1, FOS, HLA-B, ISG15, KLF10, KRT8, NMI, PTGES, PTX3, SAA1, SDC4, SMOC2, TGM2, TNFRSF10A*
CREBBP	Activated	2.216	0.0002	*CCND2, EGR1, EHHADH, FOS, HLA-B, HSD3B2, ISG15, LDLR, LGALS3BP, LPL, NR3C1, RNASEL, RSAD2, SDC4, SPSB1, USP18*
TGFB1	Activated	3.197	0.0002	*APOE, C1S, CCND2, CCT6A, CLU, COL1A1, CYP19A1, EGR1, FOS, GABRD, GPRC5B, GTPBP1, HSD3B2, IL33, ITGA2, KLF10, KRT8, LDLR, LPL, LTA4H, MAOA, MYOF, PARP3, PHGDH, PLOD2, PNP, POSTN, PTGES, PTPRC, PTX3, RASGRP3, RSAD2, S100A11, SDC4, SERPINH1, SMOC2, SNAI2, SPARCL1, SRSF6, TGM2, THBS2, TNFAIP6, TNFRSF10A, TSC22D3, TUBA1A*
IFN Beta	Activated	2.774	0.0002	*HLA-B, IFI6, IRF9, ISG15, MX1, OAS1, RSAD2, TNFSF18, USP18*
ACOX1	Inhibited	-2.345	0.0002	*ACADM, EHHADH, KRT8, LGALS3BP, LPL, MFGE8, PALMD, PDK4, TNFRSF10A*
ribavirin	Activated	2.219	0.0003	*IFI6, ISG15, MX1, RSAD2, USP18*
KRAS	Inhibited	-2.010	0.0004	*BTC, CCDC80, CLU, COL1A1, EGR1, FOS, GBA, HOPX, IFI6, IL33, IRF9, ISG15, MLC1, MX1, NR3C1, OAS1, SDC4, SNAI2, TNFRSF10A*

rosiglitazone	Activated	2.167	0.0005	*ACADM, APOE, COL1A1, CYP19A1, EPHX2, FOS, IDH3B, LPL, MAN1A2, OAS1, PDK4, PLPP3, POSTN, PTGES, RARRES2, UPK1B*
INSIG1	Inhibited	-2.611	0.0007	*APOE, FADS2, LDLR, LPL, PCYT2, PTGES, VNN1*
IRF3	Activated	2.394	0.0007	*CD58, IFI6, IFNW1, ISG15, NR3C1, OAS1, PNP, RSAD2, SNAI2, USP18*
PTGS2	Activated	2.715	0.0007	*CLU, CYP19A1, EGR1, ERBB3, FOS, LPL, TGM2, TNFAIP6, VNN1*
ERK	Activated	3.217	0.0008	*CLU, COL1A1, EGR1, FOS, KLF10, LDLR, PDK4, PTGES, SNAI2, TGM2, TUBA1A*
SOCS1	Inhibited	-2.773	0.0008	*CCND2, FOS, ISG15, MX1, OAS1, RSAD2, TGM2, USP18*
STAT1	Activated	2.618	0.0008	*APOE, C1S, CCND2, EGR1, FOS, HERC6, IFI6, IRF9, ISG15, MX1, OAS1, RSAD2, SERPING1, USP18*
MAPK8	Activated	2.377	0.0008	*APOE, COL1A1, CYP19A1, CYP27A1, EGR1, FOS, PTGES, PTX3, TSC22D3*
NFkB (complex)	Activated	3.355	0.0009	*ADAMTS9, APOE, CCND2, CD99, CLU, EGR1, FOS, G0S2, IFNW1, ISG15, PTGES, PTX3, RSAD2, SAA1, SDC4, SNAI2, TGM2, TNFAIP6, TNFRSF1A, VSNL1*
MTPN	Activated	2.433	0.0012	*CCNC, CCND2, COL1A1, FOS, TNFRSF1A, TUBA1A*
IFNA1/IFNA13	Activated	2.191	0.0012	*IFI6, ISG15, MX1, OAS1, RSAD2*
MAP2K1	Activated	2.376	0.0015	*APOE, COL1A1, FOS, GPRC5B, LDLR, RAB38, SNAI2, TGM2, TNFRSF10A*
Alpha catenin	Inhibited	-2.027	0.0017	*COL1A1, IL2RG, PTX3, SAA1, TGM2, THBS2, TNFAIP6*
Ifn	Activated	2.378	0.0018	*CD58, FOS, IL33, ISG15, MX1, RNASEL, RSAD2*
IRF4	Inhibited	-2.425	0.0021	*CCNC, ERBB3, IL33, IRF9, ISG15, OAS1, PSMA2, XCL1*
IL6	Activated	3.038	0.0021	*APOE, BTC, CLU, COL1A1, CYP19A1, EGR1, FOS, IRF9,*

				KRT8, LDLR, LIG1, LPL, NR3C1, OXTR, PCLAF, PON1, PTGES, PTPRC, SAA1, SMIM3, TGM2, TNFRSF1A
MED1	Activated	2.433	0.0021	*ANAPC5, CCND2, EHHADH, FADS2, Foxp1, NR3C1, PDK4, SCP2*
NEDD9	Activated	2.000	0.0023	*FOS, ITGA2, PLOD2, PPP1R3B*
LEP	Activated	2.144	0.0024	*ACADM, COL1A1, CYP19A1, EGR1, EHHADH, EPHX2, FADS2, FOS, INSL3, LDLR, LPL, NR3C1, SCP2, SLC27A5, TNFRSF1A*
JAK1	Activated	2.213	0.0029	*FOS, IRF9, MX1, SAA1, USP18*
F3	Activated	2.236	0.0029	*EGR1, FOS, ITGA2, PLPP3, TGM2*
JAK	Activated	2.000	0.0033	*IFI6, IL33, ISG15, RSAD2*
ACKR2	Inhibited	-2.000	0.0033	*ISG15, OAS1, RSAD2, USP18*
miR-199a-5p (and other miRNAs w/seed CCAGUGU)	Inhibited	-2.236	0.0033	*BCAM, COL1A1, ISG15, MPP5, RSAD2*
SMARCB1	Activated	2.646	0.0035	*COL1A1, F11, FOS, HOXC8, MX1, OAS1, POSTN, RND3*
ERK1/2	Activated	2.298	0.0050	*CCND2, COL1A1, CYP19A1, EGR1, FOS, ITGA2, LDLR, POSTN, PTGES, SNAI2, VNN1*
TGFB3	Activated	2.2000	0.0053	*COL1A1, CYP19A1, FOS, PLOD2, SNAI2, TNS1*
NCOA1	Inhibited	-2.236	0.0054	*ADAM22, CHD9, EGR1, ERBB3, FOS, HSD3B2, OXT*
TGM2	Activated	2.333	0.0055	*HLA-B, IFI6, IFIT5, IRF9, LGALS9, OAS1, PTGES, TTC27, UBA7*
F2	Activated	2.591	0.0055	*ADAMTS9, CCND2, CLU, COL1A1, EGR1, FOS, GABRD, PROS1, RGS3, SDC4*
IL27	Activated	2.020	0.0056	*CCND2, EGR1, FOS, HLA-B, MX1, OAS1, TNFSF18*
EGR1	Activated	2.528	0.0057	*CCND2, CLU, COL1A1, EGR1, HPSE, LDLR, PTGES, SNAI2*
tetradecanoylphorbol acetate	Activated	3.285	0.0062	*BTC, CCND2, CYP19A1, EGR1, Folh1, FOS, HPSE,*

				IL2RG, ISG15, ITGA2, LDLR, LPL, OAS1, PALMD, PDK4, PHGDH, PNP, PON1, PTGES, PTPRC, SDC4, SNAI2, TCIM, TNFAIP6, TNFRSF10A, TPBG, TSC22D3, XCL1
KITLG	Activated	2.201	0.0065	*EGR1, ETF1, FOS, FRK, LDLR, NR3C1, SNAI2, TNFRSF1A, TSC22D3*
inosine	Activated	2.000	0.0065	*C1S, LGALS3BP, SERPING1, TUBA1A*
NRAS	Inhibited	-2.128	0.0069	*ACADM, EGR1, Ifi27, ISG15, ITGA2, PTX3, USP18, VNN1*
ANGPT2	Activated	2.177	0.0077	*COL1A1, EPHX2, MFGE8, POSTN, PROS1, PTGES, SERPING1, THBS2*
MYCN	Inhibited	-2.178	0.0080	*CCND2, CLU, COL1A1, ITGA2, MSRB3, PHGDH, SLC38A2, TGM2, TM6SF1, TNFRSF1A*
EGF	Activated	2.822	0.0085	*CCND2, CLU, COL1A1, EGR1, FOS, HSD3B2, INSL3, ITGA2, KLF10, PDK4, PLPP3, PTGES, S100A11, SNAI2, TGM2*
bisphenol A	Activated	2.404	0.0087	*CLU, CYP19A1, EGR1, FOS, MAOA, OXT, POSTN*
TLR4	Activated	2.596	0.0093	*CCND2, HPSE, IFNW1, ISG15, MX1, NMI, OXTR, PLEK, PTGES, PTX3, RSAD2, TNFRSF10A*
FGF1	Activated	2.404	0.0094	*APOE, EGR1, FOS, POSTN, SNAI2, TGM2*
IL2	Activated	2.78	0.0098	*CCND2, CD58, CYP27A1, FOS, IL27RA, IL2RG, ITGA2, LDLR, MX1, NMI, PNP, PROS1, PTPRC, SCP2, TNFRSF10A, TNFRSF1A, XCL1*
EP300	Activated	2.200	0.0110	*ACADM, CYP19A1, EGR1, FOS, HLA-B, LGALS3BP, NR3C1, PDK4, RNASEL, RSAD2, SDC4, SPSB1, USP18*
VEGFA	Activated	2.390	0.0112	*ACADM, COL1A1, EGR1, FOS, ITGA2, PLPP3, RASGRP3, RHO, SNAI2, XCL1*

Ifnar	Activated	2.200	0.0114	*IRF9, ISG15, OAS1, RSAD2, USP18*
RAS	Activated	2.133	0.0116	*EGR1, FOS, LDLR, PTGES, SNAI2, TGM2*
IRF1	Activated	2.610	0.0126	*IFI6, IFIT5, IRF9, ISG15, MX1, OAS1, RSAD2*
IL21	Activated	2.646	0.0141	*CCND2, EGR1, HERC6, ISG15, PTPRC, RSAD2, USP18*
AGT	Activated	2.677	0.0142	*CCND2, CDH13, COL1A1, CYP19A1, DDAH2, EGR1, FOS, HSD3B2, ITGA2, LDLR, MATN2, OXT, POSTN*
testosterone	Activated	2.456	0.0150	*CCNC, CLU, CYP19A1, EGR1, FOS, GPC1, HSD3B2, INSL3, MAOA, MX1, PSMA2*
HNF4A	Activated	2.301	0.0170	*ACTR5, APOE, C1S, CIAO1, CYP27A1, EGR1, EMX2, F11, FRK, FUT10, FUT11, G0S2, HLA-B, HOXC8, IPO8, KLHL28, KRT8, LDLR, LTA4H, MAOA, PALMD, PDK4, PEPD, PNP, PON1, PPP1R3B, PSMA2, QRSL1, RASGRP3, RSAD2, SAA1, SCP2, SERTAD3, SLC22A3, SNAI2, SYMPK, TCIM, TNFAIP6, TTC25, VEGFD, VSNL1, ZFP37, ZNF189*
DOCK8	Activated	2.000	0.0173	*CCND2, ISG15, NMI, RSAD2*
cisplatin	Activated	2.158	0.0189	*ACADM, CLU, COL1A1, EGR1, ERBB3, FOS, G0S2, HEXD, IFI6, MAOA, MX1, NMI, PDK4, PHGDH, PTGES, S100A11, TCIM, TNFRSF1A, ZFP37*
SASH1	Activated	2.000	0.0190	*CCND2, ISG15, NMI, RSAD2*
TRAF3IP2	Activated	2.000	0.0208	*COL1A1, IL33, ISG15, POSTN*
MITF	Activated	2.828	0.0214	*APOE, CD151, COL1A1, FOS, LIG1, NR3C1, RHOQ, TDG*
REL	Activated	2.592	0.0219	*CCND2, FADS2, FOS, IFT74, PHGDH, RSAD2, SNAI2, TNFRSF10A*
miR-30c-5p (and other miRNAs	Inhibited	-2.236	0.0219	*ITGA2, PNP, SLC38A2, SLC4A10, UAP1*

w/seed GUAAACA)				
sirolimus	Inhibited	-2.882	0.0223	*ACADM, CCND2, CD151, COL1A1, EGR1, ERBB3, FOS, HLA-B, IRF9, ISG15, NR3C1, POSTN, RHOQ, VEGFD*
IL17A	Activated	2.630	0.0224	*BTC, COL1A1, FOS, IL33, ISG15, LPL, SPSB1, VEGFD*
cytokine	Activated	2.236	0.0232	*CLU, EGR1, FOS, PON1, SAA1, TNFAIP6*
IFI16	Activated	2.000	0.0237	*ISG15, LDLR, LPL, OAS1*
EPO	Activated	2.189	0.0246	*CCND2, EGR1, ETF1, FOS, GBA, H2AFZ, LDLR, NR3C1, TSC22D3*
PI3K (complex)	Activated	2.578	0.0266	*CCND2, CCT6A, COL1A1, FOS, IFNW1, IL33, POSTN, RND3, TGM2*
ATF4	Activated	2.177	0.0288	*CSRP3, CYP27A1, PHGDH, PTX3, SLC38A2, SNAI2*
HGF	Activated	2.066	0.0291	*COL1A1, EGR1, FOS, GBA, IL27RA, ISG15, ITGA2, LDLR, PLPP3, RARRES2, ST14, TNFAIP6, TNFRSF1A*
FN1	Activated	2.200	0.0301	*APOE, COL1A1, FOS, HLA-B, SDC4, SNAI2, TGM2*
camptothecin	Activated	2.828	0.0303	*APOE, FOS, HLA-B, LDLR, NR3C1, PTGES, PTX3, RGS3, RSAD2, SAA1, SDC4, STK38L, TNFAIP6, TNFRSF10A, TPBG*
Vegf	Activated	2.415	0.0315	*EGR1, ERBB3, GPRC5B, HOPX, HPSE, IL27RA, ITGA2, JAM2, LDLR, OXTR, PLOD2, PLPP3, ST8SIA4*
hydrogen peroxide	Activated	2.917	0.0328	*CDH13, CLU, COL1A1, DDAH2, EGR1, FOS, IFI6, ITGA2, LDLR, PTX3, SERPINH1, SNAI2, TGM2*
5-O-mycolyl-beta-araf-(1->2)-5-O-mycolyl-alpha-araf-(1->1')-glycerol	Activated	2.000	0.0339	*CYP19A1, IFIT5, RND3, SDC4*
TGFBR1	Activated	2.000	0.0339	*COL1A1, PDK4, RGS3, SNAI2*
FEV	Activated	2.000	0.0352	*FOS, JAM2, NR3C1, SLC22A3*
SP1	Activated	3.093	0.0354	*APOE, CCND2, CD99, COL1A1, CYP19A1, EGR1,*

				FOS, HPSE, HSD17B7, ITGA2, JAM2, LDLR, LPL, MAOA
TLR7	Activated	2.592	0.0374	*HIVEP2, IFNW1, IRF9, ISG15, MX1, PTX3, RSAD2*
CSF1	Activated	2.533	0.0382	*APOE, CCND2, EGR1, FOS, HSD17B7, PTPRC, TNFRSF1A*
IGF1R	Activated	2.393	0.0408	*ACADM, Cabp1, CLU, COL1A1, EGR1, FOS, SCP2, SDC4*
MYD88	Activated	2.592	0.0408	*EGR1, FOS, IFNW1, ISG15, PTGES, RSAD2, SAA1, USP18*
GDF2	Activated	2.000	0.0420	*COL1A1, FOS, POSTN, SPARCL1*
SAMSN1	Activated	2.000	0.0420	*CCND2, ISG15, NMI, RSAD2*
N-acetyl-L-cysteine	Inhibited	-2.588	0.0449	*CCND2, CHRNA9, COL1A1, EGR1, FOS, LPL, TNFRSF10A*
IGF1	Activated	2.903	0.0450	*CCND2, CLU, COL1A1, CYP19A1, EGR1, FOS, INSL3, LPL, NR3C1, OXT, PTPRC, SNAI2*
Tgf beta	Activated	2.095	0.0462	*CCND2, CLU, COL1A1, FOS, KLF10, PLPP3, POSTN, SNAI2*
thyroid hormone	Activated	2.236	0.0509	*APOE, EGR1, FOS, LPL, OXT, ST14*
methapyrilene	Inhibited	-2.000	0.1400	*ACADM, APOE, EHHADH, SCP2*
D-glucose	Activated	2.177	0.3360	*ACADM, CCND2, CLU, COL1A1, EGR1, FOS, KLF10, LDLR, LPL, NR3C1, TGM2*
TCF4	Activated	2.236	0.5080	*ADAMTS9, CCT6A, DNAJC12, FOS, Ifi27, SNAI2*

Supplemental Table 16. Selected upstream regulators of differentially expressed genes in oocytes from control heifers comparing day 4 to day 60.

**Upstream regulator**	**Predicted activation state**	**Activation z-score**	***P*-value of overlap**	**Target molecules in dataset**
Beta-estradiol	Inhibited	-3.947	< 0.0001	*A4GALT, ABCG2, ACADM, ACKR3, ACTA2, ACTB, ACTC1, ADM, AES, AGRN, AIF1, ALDH18A1, ALDOA, ANGPT1, ANKH, ANKRD12, ANXA1, ANXA4, AP1G1, APBB1, APCDD1, APOA1, APOE, AR, ARMT1, ASB9, ASH1L, ATF3, ATP5F1B, BAK1, BEX2, BHLHE40, BIRC3, BLNK, BMP2, BMP4, BNIP2, BNIP3L, BTG1, BTG2, C1QTNF6, CAD, CARTPT, CCDC80, CCL2, CCL25, CCL5, CCN1, CCN2, CCND2, CCND3, CCNT2, CCR5, CD14, CD44, CDH2, CEBPD, CLEC1B, CLU, COL1A1, COL1A2, COL6A6, CPXM1, CRABP2, CRHR2, CTNNB1, CTSD, CTSH, CTSK, CXCL10, CXCL12, CXCL13, CXCL2, CYP11A1, CYP19A1, DCN, DDOST, DDR1, DDX17, DFFB, DUSP1, ECE2, EGR1, EIF4EBP1, ENC1, ENO1, EPO, ERBB2, ESR1, FABP4, FADS1, FAS, FASN, FBLN1, FCGRT, FHL1, FHL2, FMO5, FMOD, FNBP1, FOS, FOSB, FST, FSTL1, FUCA1, GAL, GART, GBP2, GCLC, GCSH, GJA1, GLRB, GM2A, GMFB, GPC1, GPX1, GPX3, GREB1, GSTA1, GSTP1, GUCY1B1, HERPUD1, HIF1A, HLA-DQA1, HMOX1, HS1BP3, HSD17B1, HSD17B11, HSD17B3, HSD17B7, HSPA5, HSPB1, HSPD1, HTRA1, ID1, ID2, IDH2, IER3, IFNGR2,*

				IGFBP4, IGFBP5, IGFBP6, IL1A, IL1B, IL4R, IL6R, INHA, INHBA, INHBB, INSL3, ISG15, ITGA2, ITGAV, JUN, JUNB, KLF10, KLF6, KLK1, KMT2E, KPNA4, KRT13, KRT18, KRT8, KYNU, LAMB1, LAPTM5, LGALS3BP, LITAF, LOXL4, LPL, LRP8, LTF, LUM, LYZ, MAOA, MATN2, MBTPS1, MCM7, MET, MMD, MMP9, MX1, MYC, MYCN, MYLPF, MYOF, NDRG1, NDRG2, NECTIN1, NET1, NFE2L2, NME1, NME3, NOP58, NPR3, NPTX1, NPY1R, NR5A1, NRG1, NRTN, NTRK1, NTS, NUDT1, NUPR1, OSBPL3, OSTF1, OXT, OXTR, P4HB, PAM, PAPSS2, PBX1, PCYOX1, PDGFRA, PDK4, PDZK1IP1, PEMT, PFKL, PGK1, PGR, PKIB, PKM, PLAT, PNP, PNRC2, PPIB, PPM1K, PPP1R14A, PPP3CA, PRKCB, PROS1, PRPS1, PRSS35, PSMA1, PSMA2, PTEN, PTGDS, PTGES, PTPRN, PTX3, PYCR1, PYGL, RARA, RBM14, RBM39, Rcan1, REN, RERG, RGS1, RGS3, RGS7, RHOQ, RND3, RPS11, RPS9, RTP4, RYR2, S100A13, S100A2, SCARB1, SDC1, SDC2, SDC4, SERPINA5, SERTAD4, SETD7, SFRP4, SGCB, SGK1, SLC2A1, SLC2A3, SLC2A4RG, SLC38A2, SLK, SMPD1, SNAI2, SPARC, SPRY1, SRSF1, SRSF2, SSBP2, SSR2, SSR3, SSTR2, STC1, SYK, TAC3, TAP2, TCIM, TGFB1, THBS1, THRA, TIMP1, TIMP2, TIMP3, TM4SF1,

				TMPO, TNFAIP3, TNFAIP6, TNFRSF1A, TNFRSF1B, TNFSF11, TP53, TP53I11, TPD52L1, TPI1, TPM1, TRA2B, TRIB2, TRIM23, TSC22D3, TSPO, TXN, VCAN, VEGFA, VIM, WFDC2, WNT3, WNT5B, XBP1, XPO1, YARS, ZBTB18, ZFP36L2
CG	Inhibited	-5.626	< 0.0001	*ACTA2, ACTB, ADM, APCDD1, AR, ARNT, B2M, BHLHE40, BMP2, BTC, BTG1, BTG2, BTG3, CCL2, CCND2, CCND3, CDH2, CDK4, CEBPD, CLU, CNPY4, CTSV, CXCL10, CYP11A1, CYP19A1, DBI, DPT, DUSP1, EDN3, EGR1, FABP4, FAS, FGL2, FHL1, FOS, FST, FZD1, G0S2, GAL, GAS1, GJA1, GM2A, GPC1, HIF1A, HK2, HLA-DQA1, HPSE, HSD17B3, HSD17B7, HSD3B2, IER3, IGFBP4, IL1B, IL33, IL4R, INHA, INHBA, INHBB, ITGAV, JUN, LGALS3BP, LRRN3, MARCH3, MCL1, MMP19, MMP9, NPR3, NR2F6, OXT, OXTR, PARD6G, PFKFB3, PGR, PKIA, PLAT, PLPP3, PPP1R14A, PRSS2, PTX3, RGS20, RGS4, S100A10, SCARB1, SDC1, SDC4, SFRP4, SMAD4, SPP1, ST8SIA4, STC1, TIMP1, TIMP4, TM4SF1, TNFAIP6, TNFRSF12A, TP53, VCAN, VEGFA, VEGFC*
FSH	Inhibited	-3.107	< 0.0001	*ACTA2, ACTB, ACTG2, ADM, AMH, AR, ARHGAP1, ARL4C, ARL6IP5, BNIP3L, BTC, BTG2, CCN2, CCND2, CDH2, CDK6, CITED1, CREBZF, CSNK1A1, CTNNB1, CTSV, CYP11A1, CYP19A1, DHH,*

				DHRS3, DNAJB9, DUSP1, ELL2, ENC1, ESR1, FDXR, FOS, FOSB, FSHR, FST, GBP1, GCLC, GPRC5B, HK2, HSD17B1, HSD3B2, HSF2, ID1, IGFBP4, IL6R, ILK, ING2, INHA, INHBA, INHBB, ISG20, ITGA5, JUN, JUNB, KLF10, KRT18, LGALS1, LYPD3, LZTR1, MAP3K5, MCL1, MMP9, MYC, NOL3, NPY1R, NR5A1, P4HA2, PGK1, PGR, PKM, PLAT, PTEN, PTPRF, PTPRN, RAB14, RARA, RGS4, RGS7, RHOB, SCARB1, SERPINE1, SGK1, SHBG, SLC2A1, SMAD4, SSTR2, STX4, THBS1, TIMP1, TIMP2, TLN1, TNFAIP3, TNFAIP6, TP53, TP53I11, TPM1, TPM2, TUBA1A, VEGFA, VEGFC, YBX3, ZNF331
IGF1	Inhibited	-4.354	< 0.0001	*ACTA2, ACTR1B, ADM, ANGPT2, BAK1, BHLHE40, BIRC3, BMP2, BMP4, BTG2, C1QA, CAMP, CCL3L3, CCL5, CCN1, CCN2, CCND2, CCND3, CD44, CDK4, CEBPD, CLU, COL1A1, CTNNB1, CTSD, CYBA, CYP11A1, CYP19A1, DDX5, DUSP1, EGR1, EIF4B, ESR1, FABP4, FASN, FGF23, FOS, FOSB, FSHR, GAPDH, GCK, GPD1, HIF1A, HMOX1, HSD17B11, HSD17B3, HSPA5, ID1, ID2, IER3, IFNGR2, IGFBP4, IGFBP5, IGFBP6, IHH, IL1B, IL4R, INHA, INSL3, ITGA5, ITGAV, JUN, JUNB, KLF6, LPL, MCL1, MIF, MMP14, MYC, MYCN, MYH11, NFE2L2, NOG, NR5A1, NTRK1, NUPR1, OXT, PDGFRA, PGR,*

				PKM, *POLD4*, *PPP3CA*, *PSMB8*, *PSMB9*, *PTEN*, *PTPRC*, *PYY*, *Rcan1*, *SCD*, *SERPINE1*, *SGK1*, *SHBG*, *SLC12A6*, *SLC2A1*, *SNAI2*, *SPP1*, *SRSF3*, *TAP1*, *TAP2*, *TG*, *TGFB1*, *THBS1*, *TMED1*, *TNFRSF12A*, *TNNI3*, *TP53*, *UGCG*, *VEGFA*, *VEGFC*, *VIM*, *WDR45*, *XBP1*, *YBX3*, *ZFP36L2*
Dihydrotestosterone	Inhibited	-3.092	< 0.0001	*ACKR3*, *ACTB*, *ADM*, *AES*, *AIG1*, *ALDOA*, *APOE*, *APOM*, *AR*, *ARFGAP3*, *ATP13A2*, *B2M*, *BTG2*, *CCN2*, *CCND2*, *CCND3*, *CD36*, *CDH2*, *CLU*, *COL4A1*, *COL6A1*, *CTNNB1*, *CYP11A1*, *CYP19A1*, *DBI*, *DCN*, *DCTPP1*, *DDX5*, *EGR1*, *EIF4EBP1*, *ENO1*, *ERBB2*, *ESR1*, *FABP4*, *FASN*, *FOS*, *Foxp1*, *FSHR*, *FST*, *FTH1*, *G0S2*, *GAPDH*, *GPD1*, *GPX3*, *GREB1*, *GRN*, *GSN*, *GSTP1*, *HECTD2*, *HK2*, *HMGN1*, *HSD3B2*, *HSPB1*, *ID2*, *IGFBP4*, *IGFBP5*, *IGFBP7*, *IL1A*, *INHBB*, *ITGA2*, *JUN*, *KLK1*, *KLK4*, *KRT1*, *LGALS1*, *LPL*, *LYZ*, *MARK1*, *MMP9*, *MYC*, *NDRG1*, *NNAT*, *NUCB1*, *OXT*, *OXTR*, *PAM*, *PC*, *PHGDH*, *PMEPA1*, *POSTN*, *POU2F1*, *PROS1*, *RARRES2*, *RHOB*, *RPL12*, *RPL3*, *RPL30*, *RPL37A*, *RPL8*, *RPS12*, *RPS17*, *RPS20*, *RPS24*, *RPS26*, *RPS29*, *SCD*, *SCP2*, *SERPINE2*, *SFRP4*, *SGK1*, *SLC16A7*, *SLC22A16*, *SLC2A1*, *SLC2A3*, *SNAI2*, *SNAI3*, *SOD3*, *SPARC*, *SPP1*, *SRGAP2*, *ST6GALNAC5*, *STBD1*, *TGM2*, *THBS1*, *TIMP2*, *TIMP3*, *TKT*, *TNNI3*,

				TP53, UAP1, VCAN, VEGFA, VIM, VLDLR, VPS54
FOS	Inhibited	-2.341	< 0.0001	*ACTG1, ANXA4, ARNT, ARPC5, ATF3, CADM1, CAMP, CAPN2, CCL2, CCL5, CCN2, CD14, CD44, CD68, CLU, COL1A1, COL6A1, CRABP2, CTSH, CTSV, CXCL16, DAP, EGR1, ENO1, EPHX2, FABP4, FASN, FOLR2, FOS, FOSB, FSTL1, FTH1, GAS1, GAS2, GBA, GJA1, GM2A, GREB1, GSTP1, HDAC6, HLA-B, HMOX1, HSD3B2, HSPA5, IGFBP5, IGFBP6, IL1A, ITGA6, JUN, JUNB, KIRREL3, KLF6, KRT13, KRT8, LGALS3BP, LPL, LRP8, MAOA, MAP3K20, MET, MMP9, MYC, NET1, NFE2L2, NGF, NPTX1, NTS, OXTR, PCDHGC3, PCYT2, PGR, PKIB, PKN1, PLAT, PMEPA1, PSMA2, PTGDS, PTPRN, RARA, RGS4, RNH1, RPLP0, RPS18, RPS24, RPS6, RPS9, S100A10, S100A8, SCD, SDC1, SERPINE1, SERPINE2, SFRP4, SFTPD, SIRPA, SNAI2, SPHK1, SPP1, ST8SIA5, STMN1, SYT1, TGFB1, TIMP1, TLL2, TNFSF11, TP53, TPP1, TUBB, TXN, UGCG, UTRN, VEGFA, VIM, VSX1, XAF1, XYLT1*
Prostaglandin E2	Inhibited	-3.506	< 0.0001	*ACTA2, ANGPT1, ANGPT2, ANGPTL4, ANXA2, ATF3, BAK1, BIRC3, BMP2, BTG1, CAMP, CASP7, CCL2, CCL22, CCL3L3, CCL5, CCL8, CCN2, CCR5, CD14, CD44, CD83, CEBPD, COL1A1, CXCL10, CXCL12, CYP19A1, DCN, DUSP1, EGR1, ERBB2, FAS, FASN, FMOD, FOS, FOSB,*

				FST, FSTL3, GJA1, HIF1A, HMOX1, HSD3B2, ID1, IER3, IGFBP4, IGFBP5, IGFBP7, IL15RA, IL1A, IL1B, IL2RG, ITGA5, JUN, MBTPS1, MCL1, MMP14, MMP19, MMP9, MYC, PGF, PGR, PKLR, PKN1, PTGES, S100A8, SCN9A, SPHK1, SPP1, ST3GAL4, TGFB1, THBS1, TNFAIP6, TNFSF11, TP53, TSC22D3, UCP2, VEGFA, VEGFC, VIM
EGFR	Inhibited	-4.022	< 0.0001	*ABCG2, ACTA2, ACTB, ANXA1, AR, ATAD3A, BTC, BTG1, CAD, CAPZA2, CBX5, CCL2, CCL5, CCN2, CD47, CDK4, CDK6, COL1A1, COL6A1, CTNNB1, CTSK, CXCL10, CXCL12, CYP19A1, DUSP1, EGR1, ERBB2, ESR1, FASN, FKBP11, FOS, GBP1, GJA1, GPC1, GRN, HERPUD1, HIF1A, HK2, HNRNPA2B1, HNRNPDL, HSPA5, HSPB1, ID1, IER3, IGFBP4, IGFBP5, IL1B, IL33, ITGA2, ITGA6, IVNS1ABP, JUN, JUNB, KPNB1, LAMA4, LRP8, MACF1, MARCKS, MATN2, MCL1, MET, MMP14, MMP9, MTA2, MYC, PABPC1, PBX1, PKM, PLAT, POSTN, PRF1, PTEN, PTGES, RHOB, RRAS, S100A2, SERPINE1, SERPINE2, SLC2A1, SLC2A3, SNAI2, SPHK1, SRSF1, ST3GAL2, TGM2, THBS1, TNFAIP6, TNFSF11, VCAN, VEGFA, VIM*
Progesterone	Inhibited	-2.393	< 0.0001	*ABCG2, ACAT1, ACTA2, ACTB, ADM, AMY2A, ANKH, AP1G1, APOE, AR, ATF3, BHLHE40, BIRC3, BMP2, CADM1, CCL2, CCL25,*

				CCND3, CCR5, CD36, CDH2, CEBPD, CES1, CNDP2, CTSF, CTSV, CXCL10, CXCL12, CYP19A1, DBP, DLC1, ECE2, EDN3, EGR1, ELL2, ENC1, ERBB2, ESR1, FAM49A, FKBP3, FMO5, FOS, FSHR, FST, FTH1, GABARAPL1, GABRD, GAL, GJA1, GREB1, GSTA3, HIF1A, HLA-DQB1, HOPX, ID1, IDH2, IGFBP5, IHH, IL1A, IL6R, INHA, INHBA, ISG20, ITGA2, ITGA5, JAM2, JUN, KLF10, KLK1, LGALS1, LITAF, LPL, LTF, LXN, MAOA, MCM7, MMP9, MYC, MYCN, NDRG1, NDRG2, NET1, NOP16, NPY1R, NTRK1, OXT, PARM1, PDZK1IP1, PFKFB3, PGR, PIP5K1A, PKM, PLA2G7, PLPP3, POSTN, PPP2R5A, PROS1, PRSS35, PTGDS, PTX3, RTN4, SCD, SFRP4, SGK1, SLC2A1, SLC2A3, SPHK1, SPP1, SPSB1, ST3GAL4, TGFB1, TIMP1, TIMP3, TNFRSF1A, TNFRSF21, TNFSF11, TP53, UGCG, VEGFA, VEGFB, VIM, WFDC2, WNT5B
Lh	Inhibited	-2.634	< 0.0001	*ACTA2, ACTB, ACTG2, AMH, AR, ARHGAP1, ARL4C, ARL6IP5, BNIP3L, BTC, CCND2, CSNK1A1, CTNNB1, CYP11A1, CYP19A1, DHRS3, DUSP1, FDXR, FOS, FST, GBP1, GJA1, GPRC5B, HK2, HSD17B1, HSD17B3, HSD3B2, ILK, INHA, INHBB, INSL3, KRT18, MAP3K5, MCL1, MMP19, MMP9, NOL3, NR5A1, P4HA2, PGK1, PGR, PLAT, PTPRF, PTPRN, PTX3, RAB14, RGS4, RGS7,*

				RHOB, SFRP4, SGK1, THBS1, THBS2, TLN1, TNFAIP3, TNFAIP6, TNFRSF12A, TP53, TP53I11, TPM1, TPM2, TUBA1A, VEGFA, VEGFC, YBX3
ESR2	Inhibited	-2.151	< 0.0001	*A4GALT, ACTC1, AES, AK7, ANGPTL2, ANXA4, APOE, AR, ARRDC4, ATF3, BMP2, BMP4, CAPN2, CCL2, CCN2, CDH2, CEBPD, CES1, CLEC1B, COL1A1, CTNNB1, CTSD, CXCL12, CYP11A1, CYP19A1, DDAH1, EGR1, ESR1, FASN, FBLN1, FHL1, FOS, GCLC, GCSH, GLB1, GM2A, GREB1, GSTP1, HEBP1, HSD17B3, HSD17B7, IGFBP4, IL1B, ITGA2, JUNB, KCNK15, KRT8, LAPTM5, LOXL4, LPL, LRRN3, MAOA, MMP14, MYC, NDRG1, NDRG2, NTRK1, OXT, PCYOX1, PDK4, PGR, PKIB, POLD4, PTGDS, PTX3, PYCR1, PYGL, RARA, RHOQ, SCARB1, SERTAD4, SFRP4, SGK1, SLC2A4RG, SNAI2, SPP1, ST8SIA4, SUSD4, TGM2, TIGD3, TIMP1, TIMP2, TNFSF11, TP53I11, TPM1, TRIM23, TSPO, VEGFA, ZBTB18*
